# Exploring the Influence of Carbon Nanoparticles on the Formation of β-Sheet-Rich Oligomers of IAPP_22–28_ Peptide by Molecular Dynamics Simulation

**DOI:** 10.1371/journal.pone.0065579

**Published:** 2013-06-05

**Authors:** Jingjing Guo, Jiazhong Li, Yan Zhang, Xiaojie Jin, Huanxiang Liu, Xiaojun Yao

**Affiliations:** 1 School of Pharmacy, Lanzhou University, Lanzhou, China; 2 State Key Laboratory of Applied Organic Chemistry and Department of Chemistry, Lanzhou University, Lanzhou, China; University of Akron, United States of America

## Abstract

Recent advances in nanotechnologies have led to wide use of nanomaterials in biomedical field. However, nanoparticles are found to interfere with protein misfolding and aggregation associated with many human diseases. It is still a controversial issue whether nanoparticles inhibit or promote protein aggregation. In this study, we used molecular dynamics simulations to explore the effects of three kinds of carbon nanomaterials including graphene, carbon nanotube and C_60_ on the aggregation behavior of islet amyloid polypeptide fragment 22–28 (IAPP_22–28_). The diverse behaviors of IAPP_22–28_ peptides on the surfaces of carbon nanomaterials were studied. The results suggest these nanomaterials can prevent β-sheet formation in differing degrees and further affect the aggregation of IAPP_22–28_. The *π–π* stacking and hydrophobic interactions are different in the interactions between peptides and different nanoparticles. The subtle differences in the interaction are due to the difference in surface curvature and area. The results demonstrate the adsorption interaction has competitive advantages over the interactions between peptides. Therefore, the fibrillation of IAPP_22–28_ may be inhibited at its early stage by graphene or SWCNT. Our study can not only enhance the understanding about potential effects of nanomaterials to amyloid formation, but also provide valuable information to develop potential β-sheet formation inhibitors against type II diabetes.

## Introduction

Nanoparticles are highly promising candidates for various important biological applications, such as gene delivery [1], cellular imaging [2], and tumor therapy [3]. Meanwhile, the interaction between nanoparticles and the biological systems has received great attention since this may bring some biosafety concerns [4]–[7]. Among numerous types of nanomaterials, carbon nanomaterials have attracted particular interests, such as typical sp^2^-carbon nanomaterials with hydrophobic surfaces: zero-dimensional (0D) fullerene, one-dimensional (1D) carbon nanotubes (CNTs) and two-dimensional (2D) graphene. NPs are small enough to enter almost all compartments of the organism, including cells and organelles, which will complicate the pattern of protein interactions. When NPs are introduced in a living organism, their surfaces may perturb the native structure of proteins [8] as well as self-assembly pathway of peptides or proteins [9], [10].

A lot of researches indicate NPs can interfere with amyloid formation [11]–[17]. However, whether nanomaterials inhibit or promote amyloid formation is still a controversial issue. Experimental studies indicate that the diverse effects of fullerene [11]–[14], carbon nanotube [15], [16], graphite [17], [18] and mica [17] on amyloid formation depend on the intrinsic property of the peptide and the surface, and the way they interact with each other. Catalysis of the process may occur by increasing local protein concentration and accelerating the rate of nucleation on the NP surface, whereas tight binding or a large particle/protein surface area may lead to inhibition of protein aggregation [19]. Despite these observations, the detailed processes underlying the association of peptides or proteins on surfaces of NPs have so far remained elusive.

It is well known that amyloidosis is a class of disease defined by the misfolding and aggregation of functional protein precursors into fibrillar states. Amyloid fibers contribute to the pathology of many diseases, including type II diabetes, Alzheimer’s disease, and Parkinson’s disease [20]–[24]. In these disorders, amyloid fibers are present in affected tissues. However, it has become clear that intermediate states, rather than mature fibers, represent the cytotoxic species [25], [26]. Islet amyloid polypeptide (IAPP, or amylin) is a hormone coexpressed with insulin by pancreatic islet β-cells and its abnormal aggregation into amyloid fibrils is a hallmark of type II diabetes [27]. As for type II diabetes, although the molecular mechanism of its pathogenesis remains elusive, there is also evident that the key pathological species are transient β-sheet-rich oligomers of IAPP, which therefore represent therapeutic targets for treatment of type II diabetes [26], [28]–[30].

Due to the wide use of NPs in biomedicinal field, it is interesting and necessary to evaluate whether NPs affect the structure and function of the proteins in human body, especially those proteins which are easier to misfold and aggregate, and further leading to the occurrence of related disease. Such information is not only valuable for design safe and effective nanoparticles, but also investigating the mechanism of protein misfolding disease. If NPs can inhibit the process of the formation of amyloid fibrils, they will have great potential to be used as valuable therapeutic materials to control amyloid diseases like Alzheimer’s disease [31]–[34]. However, if NPs promote the aggregation of peptides or proteins, it will cause toxicity. Therefore, in this work, we will present a systematic study to investigate how the oligomer of hIAPP_22–28_ forms and the effects of different carbon NPs including graphene, single-wall carbon nanotube (SWCNT) and fullerene (C_60_) on the oligomer formation pathway. Our findings will give valuable information for further understanding the interaction between IAPP_22–28_ and carbon NPs, and provide insights into the safety of carbon nanomaterials when they enter human body.

## Materials and Methods

### Model Built and Molecular Dynamics Simulations

In our simulations, the IAPP_22–28_ (NFGAILS) peptides were capped with ACE and NME at two ends. The initial structure of the peptide was generated by a 10 ns molecular dynamics (MD) simulation at 500 K. Three classes of carbon NPs were used to explore their effects on the oligomerization process of disordered IAPP_22–28_ peptides: graphene (with dimensions of 4.92 nm×5.40 nm and 7.13 nm×5.40 nm for the tetramer and octamer, respectively), capped (5, 5)-SWCNT (3.69 nm in length and 0.68 nm in diameter), and C_60_. The atomic coordinates of NPs were provided in the Supporting Information (Text S1, S2, S3, S4). The disordered IAPP_22–28_ tetramer and octamer in the absence of NP were also simulated. The initial minimum distance between peptides and the NP surfaces is more than 5 Å, and we also ensure that the peptides are well separated not contacting with each other at the beginning of the simulations. The detailed setup information including the initial place of NP and peptides together with PBC information for each system can be found in the Supporting Information (Figure S1 and Table S1). Initially, the NPs and peptides were well separated, and the complex systems were then solvated in a rectangular box with periodic boundary conditions, and the minimum distance between the solutes and the box boundary was chosen to be about 0.8 nm as reference [35].

All MD simulations were carried out using the AMBER 10.0 package together with the ff99SB force field [36]. The TIP3P [37] solvent model was used to describe water. 2 fs time step was used to integrate the equations of motion. The long-range electrostatic interactions were treated with the particle mesh Ewald method [38]. A nonbond pair list cutoff of 1.0 nm was used. All bond lengths were constrained by using the SHAKE algorithm [39]. Temperature (310 K) and pressure (1 atm) were controlled by the Berendsen thermostat and barostat [40] with coupling constants of 0.1 and 1.0 ps, respectively. Initial configurations were minimized in three steps, first keeping the peptides fixed, and then only keeping the backbones fixed, and finally keeping all of the molecules free. The systems were warmed up from 0 t o 310 K. Equilibration and subsequent MD stages were carried out without any restrictions on pepetides in the isothermal isobaric (NPT) ensemble. However, a weak force of 1.0 kcal mol^−1^ Å^−2^ was put on the carbon NPs to keep them in the similar position and do not rotate “outside” of the solvent box during the simulation [41]. For all simulations, the atomic coordinates were saved every 2 ps for analysis.

### MD Trajectory Analysis

The trajectories of molecular dynamics simulations were analyzed using AMBER [42] and VMD [43] programs. To see the dynamics process of peptide adsorption, the contact number of atoms between peptides and NPs with a criterion of 3.5 Å over the whole simulation time was calculated. To further probe the interaction between NP and peptides, we determined the probability distribution of the minimum distance between the side chain of each residue and the nanomaterial surface for the last 50 ns simulation. The STRIDE algorithm [44] was used to compare the impacts of NPs on the secondary structure of IAPP_22–28_ peptides. Here, the β-sheet size is defined as the number of strands in an n-strands β-sheet, e.g., the β-sheet size of four-strands β-sheet is four. Two chains are considered to form a β-sheet if (i) at least two consecutive residues in each chain visit the β-strand state; (ii) they have at least two inter-peptide H-bonds. One H-bond is taken as formed if the Donor… Acceptor distance is less than 0.35 nm and the Donor-H… Acceptor angle is less than 30° in VMD [43].

## Results and Discussion

### The Dynamics Association of Oligomers Formed without Nanoparticle

The central region of amylin, residues 20–29, has been proved to be a key section of amyloid formation. Previous experimental results indicate that residues 22, 24, and 26–28 play a key role in formation of amyloid by amylin [45]. And most attention has been paid to the hexapeptide NFGAIL (human IAPP_22–27_), which is an ideal model system for theoretical studies of early oligomerization, fibril formation, and aggregates of IAPP for its small size via various techniques, including experimental and computational methods [46]–[52]. In this work, we focus on another fragment of the central region of amylin, hIAPP_22–28_, the fibril structure of which was reported belonging to the antiparallel hetero zipper class [53], to investigate the effects of different carbon NPs on its aggregation. As a comparison, we performed control runs for IAPP_22–28_ peptides in solvent only (without NPs).

**Figure 1:**
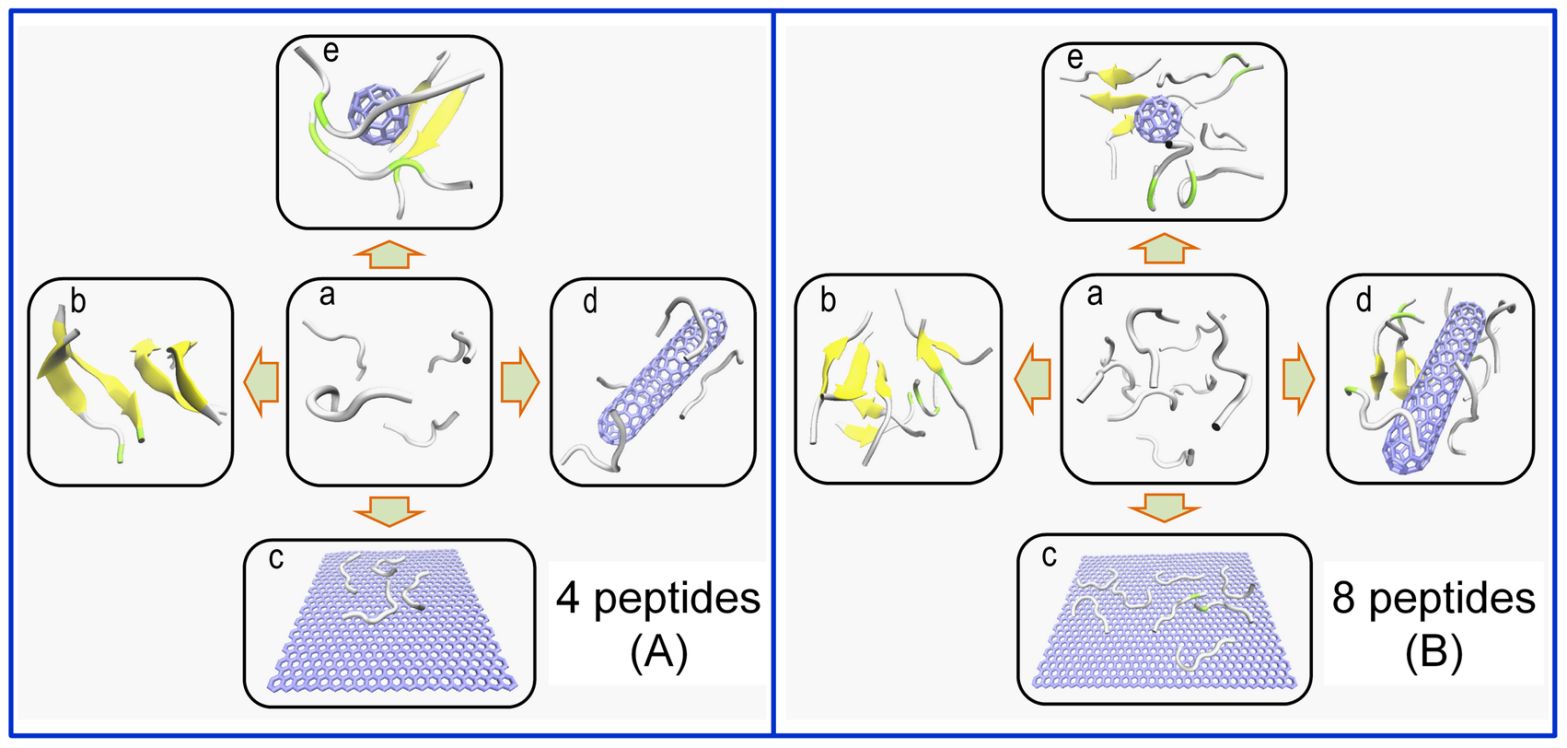
Schematic diagram of the effects of carbon NPs on the oligomerizations of initial disordered IAPP_22–28_ peptides: (A) for four peptides; (B) for eight peptides. Peptides are shown as cartoon, with β-sheet in yellow, β-bridge in lime, and others in white. The NPs are shown as sticks in ice blue. In the two sets, a presents the initial structure of 4-/8-peptide system without NP, and b presents their conformations after 200 ns simulations. In addition, c, d, and e present the corresponding conformations of the peptides interacting with graphene, SWCNT, or C60 after 200 ns simulations, respectively.

**Figure 2:**
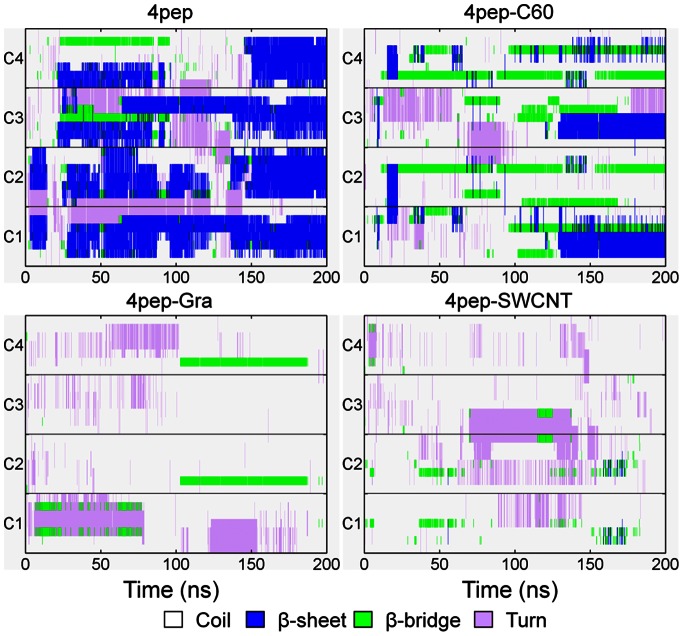
Secondary structure profile for four IAPP_22–28_ peptides in the absence or presence of carbon NPs. The four peptides are labeled from C1 to C4, respectively.

**Figure 3:**
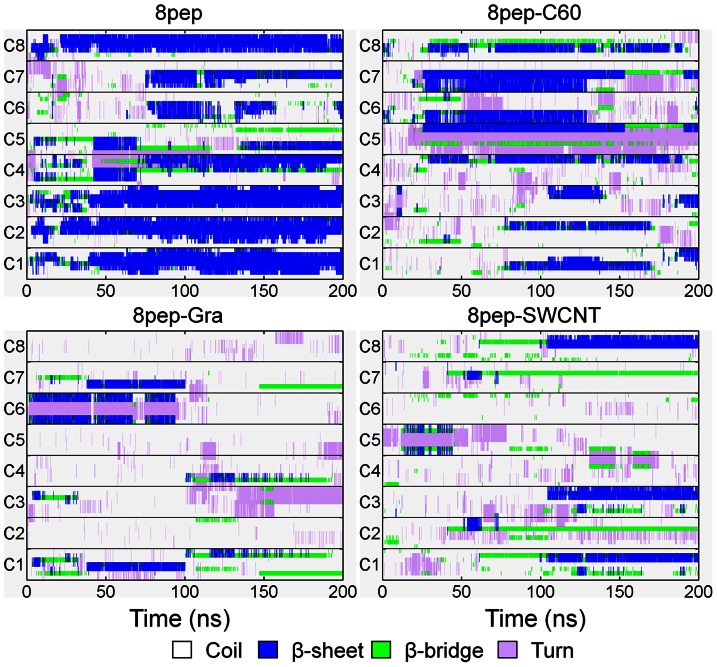
Secondary structure profile for eight IAPP_22–28_ peptides in the absence or presence of carbon NPs. The eight peptides are labeled from C1 to C8, respectively.

**Figure 4:**
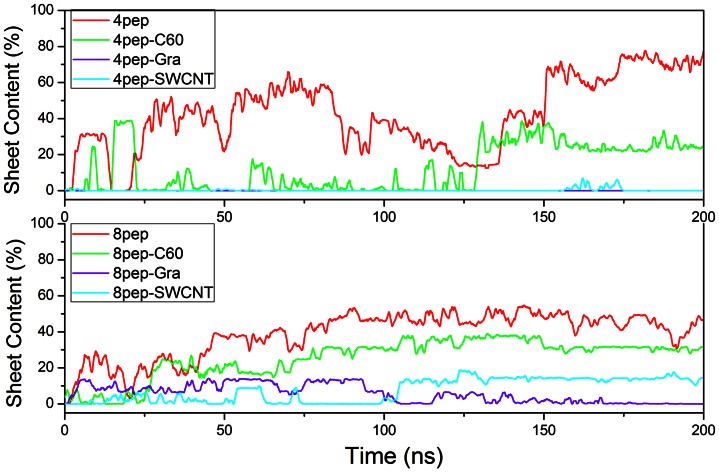
Time series of β-sheet contents for IAPP_22–28_ peptides in the absence or presence of NPs.

**Figure 5:**
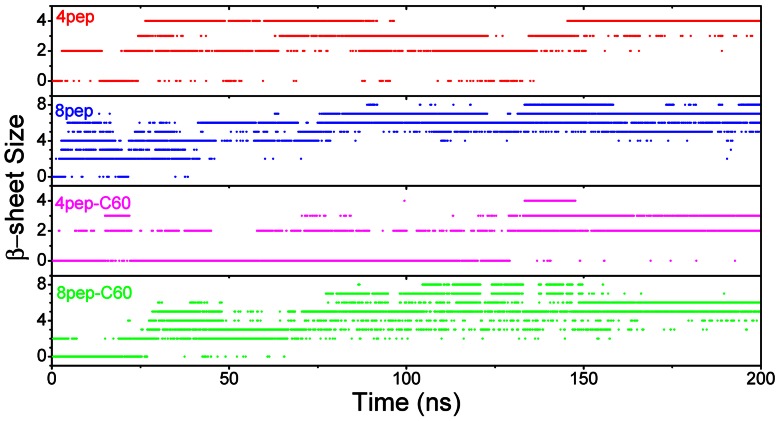
The distribution of different β-sheet size for IAPP_22–28_ peptides with or without C_60_.

**Figure 6:**
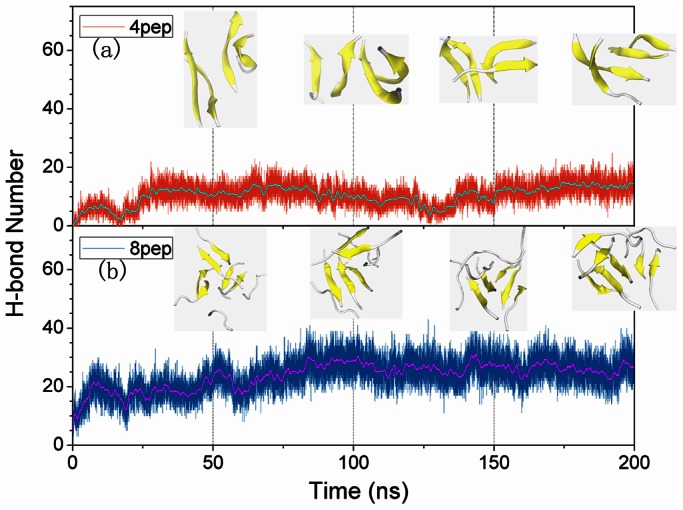
The number of backbone hydrogen bonds and structural evolution: a) four peptides without NPs; b) eight peptides without NPs. Peptides are shown as cartoon: β-sheet in yellow, and others in white.

After 200 ns simulations, we observed that without NPs, the systems with 4 or 8 well separated and disordered peptides have turned into β-sheet-rich tetramer and octamer, respectively (Figure 1). More specifically, in the absence of NPs, the conversions of initial random peptides to β-sheet-rich ones are very quick (Figure 2 and 3). As can be seen, both systems with 4 peptides and 8 peptides have high β-sheet contents around 75% and 50% in the last 50 ns simulations (Figure 4), respectively. In order to investigate how the oligomers form, we monitored the largest β-sheet size in each frame over the simulation time. As Figure 5 shows, both these two systems have high proportion of large β-sheet size relative to their peptide number, and this suggests that hIAPP_22–28_ itself has a strong ability for self-assembly. To explore the role of hydrogen bonds in the aggregation of hIAPP_22–28,_ the number of hydrogen bonds between peptides were monitored and given in Figure 6. It can be seen that the hydrogen-bond (H-bond) number of four peptides increased and achieved balance after the first 25 ns, and in the eight peptides it increased more quickly in the initial phase. In the beginning, all of the peptides are in disordered structure state, and the numbers of H-bonds are very small, while later, most of the peptides are in extended conformations (Figure 2 and 3). Our results suggest the H-bond number for eight peptides is more stable than that for four peptides. In addition, the H-bond numbers and β-sheet contents have the same change tendency, which indicates H-bond interaction plays an important role in the formation of hIAPP_22–28_ β-sheet-rich oligomers.

In summary, without the effects of carbon NPs, hIAPP_22–28_ peptides are inclined to form partially ordered β-sheet-rich oligomers with high β-sheet contents for both four and eight peptides. At the same time, the aggregation process was very quick. It’s well known that amyloid fibrils are generally formed by peptides in extended conformations (β-strands) into β-sheets through parallel or antiparallel hydrogen bonding bridges, which further stack tightly through steric effects at a completely dry interface, called a zipper [54]. Hence, the hydrogen bonds are considered to play an important role in the β-sheet formation, and this is also confirmed in our present work.

### Effective Adsorption as the First Step of the Interaction of IAPP_22–28_ and Carbon Nanomaterials

In all six trajectories for the carbon NP and IAPP_22–28_ systems, the peptides were adsorbed to the surfaces firstly, especially the surfaces of graphene and SWCNT. As Table S1 and Figure 1 shows, IAPP_22–28_ peptides and NPs were well separated initially, however, after 200 ns simulations, they were lying flat on the graphene surface or surrounding the SWCNT due to their strong interactions with the surfaces.

**Figure 7:**
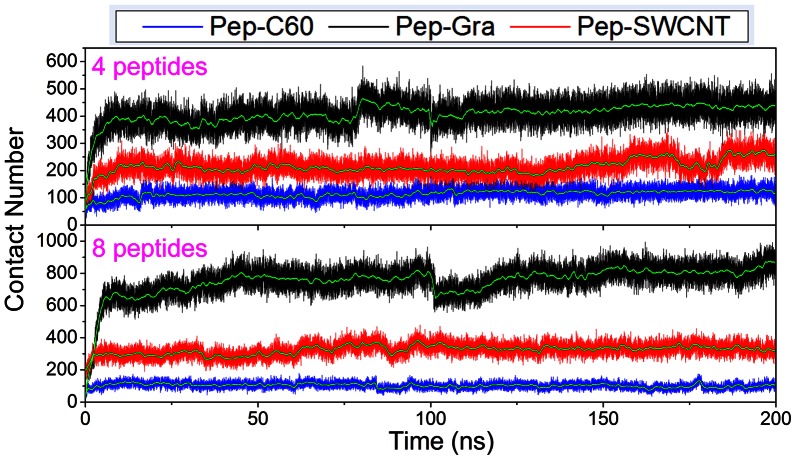
Contact numbers between peptides and nanoparticles over the whole simulation time. For clarity, a windowed average is shown as a solid green line for each system.

In order to investigate the adsorptive behaviors of the studied peptide, we counted the contact number between atoms of peptides and the different NPs over the 200 ns simulation time with a criterion of 3.5 Å (Figure 7). As can be seen, the peptides experienced initial fast structural relaxation, and were adsorbed on the surface quickly at the first 5 ns, and then the contact number of atoms was relatively up to a stable state, suggesting the interaction is steady after a rapid adsorption. For systems with four peptides, the contact number for graphene is around 400, and that with SWCNT and C_60_ are around 200 and 100, respectively. As for eight peptides, the contact numbers are around 800, 300 and 100 for graphene, SWCNT and C_60,_ respectively. It is obviously that the adsorption capacity of graphene is the strongest, and that of C_60_ is the weakest. Accordingly, graphene shows higher binding affinity with peptides than the other two carbon NPs.

**Figure 8:**
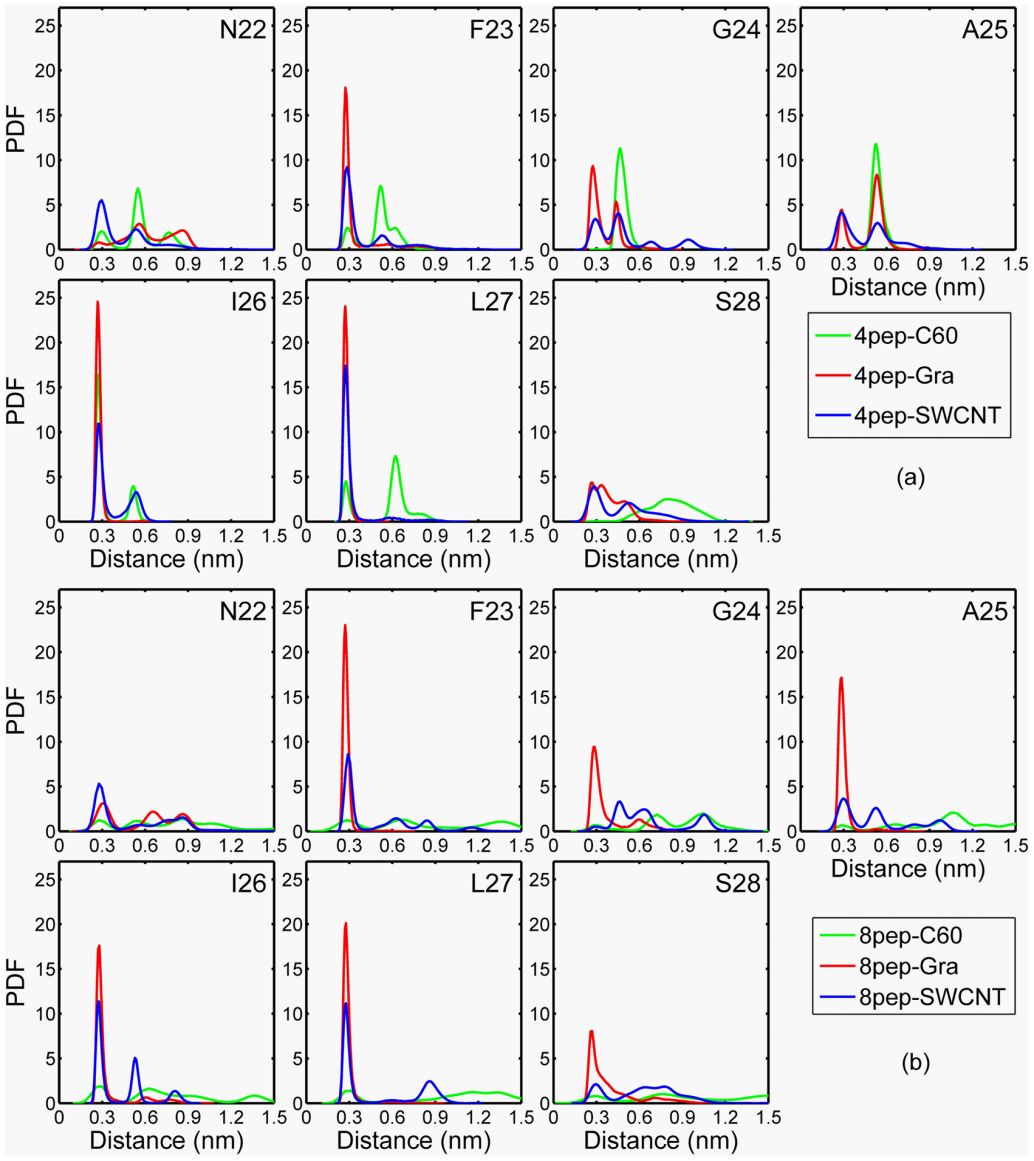
Probability distribution of the minimum distance between the side chain of each residue and the NP surface. Only the last 50 ns simulations are considered.

**Figure 9:**
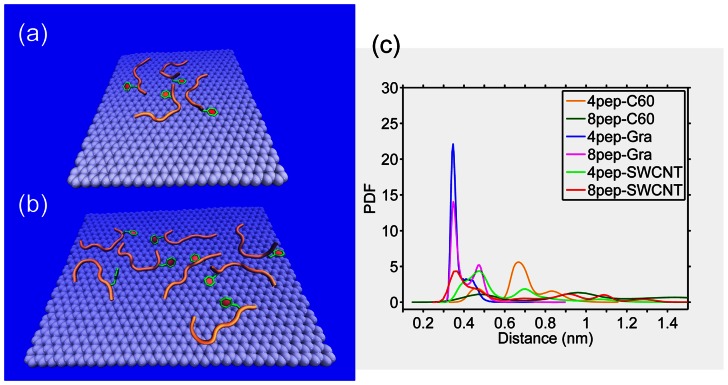
Detailed information of π–π stacking interaction between F23 residues and NPs: a). Representative structure of graphene interacting with 4 IAPP_22–28_ peptides; b) Representative structure of graphene interacting with 8 IAPP_22–28_ peptides; c) Probability distribution of the average distance between heavy atoms of F23 side chain and NPs. The heavy atoms of F23 side chain are shown as sticks, and peptides are shown as cartoon.

To further understand the adsorption mechanism and the preference of amino acid, we plotted the probability distribution of the minimum distance between the side chain of each residue and NP surface for the last 50 ns simulation in Figure 8. From Figure 8, it can be seen that there are more than one peak for most residues, but the dominant one is centered at about 0.30 nm, especially for hydrophobic residues, F23, I26, and L27. Interestingly, at about 0.30 nm almost every residue has the highest probability to interact with graphene compared with SWCNT and C_60_, which also indicates that the graphene sheets have the strongest adsorption ability compared with that of SWCNT and C_60_. This is consistent with the results of contact number. Figure 9a and 9b showed the peptides were firmly adsorbed on the graphene surface. From the representative structures of the peptides and graphene shown in Figure 9a and 9b, we can see that the aromatic residues are very close to the graphene surface. To further understand the role of the *π–π* stacking interaction in the adsorption process, we calculated the distances between the side chains of aromatic residues and the NP surfaces for the last 50 ns. The probability distributions were shown in Figure 9c. Here, the distance of a residue is defined as the average distance of its side chain non-hydrogen atoms from the surfaces. Generally, when a benzene or indole ring is adsorbed onto the graphene in the flat mode (i.e., the *π–π* stacking mode), the distance between them is ∼4.0 Å. As can be seen, the probability distribution of the distances is highest at 0.35 nm in both graphene systems. However, for the rest systems, their F23 side-chains have very small probabilities within 4.0 Å of the NP surfaces. This finding also indicates that the aromatic residue of IAPP_22–28_ fragment plays an important role on its strong adsorption to graphene surface.

The contact numbers for C60 are only around 100 in both systems due to its small surface area. The maximum probability distribution of the minimum distance between each side chain of IAPP and C60 are very small around 0.3 nm except I26 in four peptides. In addition, the probability distributions around 0.3 nm are all very low except I26 in four peptides and the probability distribution is decentralized in the 8pep-Gra system. These indicate C60 has a weaker interaction with IAPP_22–28_ peptides.

### The Presence of NP Reduces β-sheet Content in Oligomers and Affects the Aggregation of IAPP_22–28_

For the initial disordered four-peptide systems, via interacting with graphene or SWCNT, only a few β-sheets are observed, and almost all peptides adopt coil structures (Figure 2, 3 and 4). It is remarkable both 4-peptide systems with SWCNT and graphene have almost no β-sheet structure. When increasing the number of peptides from four to eight, we found the β-sheet content for SWCNT increased from around 0 to around 20% while that for graphene decreased to 0.0%. However, the C_60_ systems had much higher β-sheet contents compared with the other NP systems but lower than the systems without NPs. Obviously, the presence of NPs reduces the β-sheet contents of IAPP_22–28_ peptides. With the interaction of graphene or SWCNT, few residues present extended conformation and almost all of them are adsorbed on the surface.

In order to study the effects of C_60_ on the aggregation of IAPP_22–28_ more clearly, we monitored the largest β-sheet size over simulation time for systems with or without C_60_ (Figure 5 and Table 1). As can be seen, for the two systems without NPs, during the simulation time, initial disordered structures formed partly ordered β-sheet oligomers, and in the last 50 ns the β-sheet size with the largest content are 97.47% (size 4) and 67.5% (size 7), respectively, suggesting that the IAPP_22–28_ peptide has an obvious tendency for self-assembly and forming β-sheet-rich oligomers. When C_60_ was added, the size of dominant oligomers was reduced to two (73.74%) and six (81.04%) for four and eight IAPP peptides, respectively. Therefore, the addition of C_60_ is also bad for the β-sheet-rich aggregation of IAPP_22–28_, but in a much smaller degree compared with graphene and SWCNT.

**Table 1:** Contents of different β-sheet sizes for 4 or 8 peptides with or without C_60_ in the last 50 ns simulations.

β-sheetsize	Tetramer(%)	4 Pep+C_60_(%)	Octamer(%)	8 Pep+C_60_(%)
1	0	0.06	0	0
2	0.44	**73.74**	0.01	0.06
3	2.09	26.20	0.01	3.17
4	**97.47**	0	0.12	0.42
5	/	/	6.41	15.02
6	/	/	8.19	**81.04**
7	/	/	**67.50**	0.15
8	/	/	17.77	0.14

The largest percentage of each system is shown in bold.

**Figure 10:**
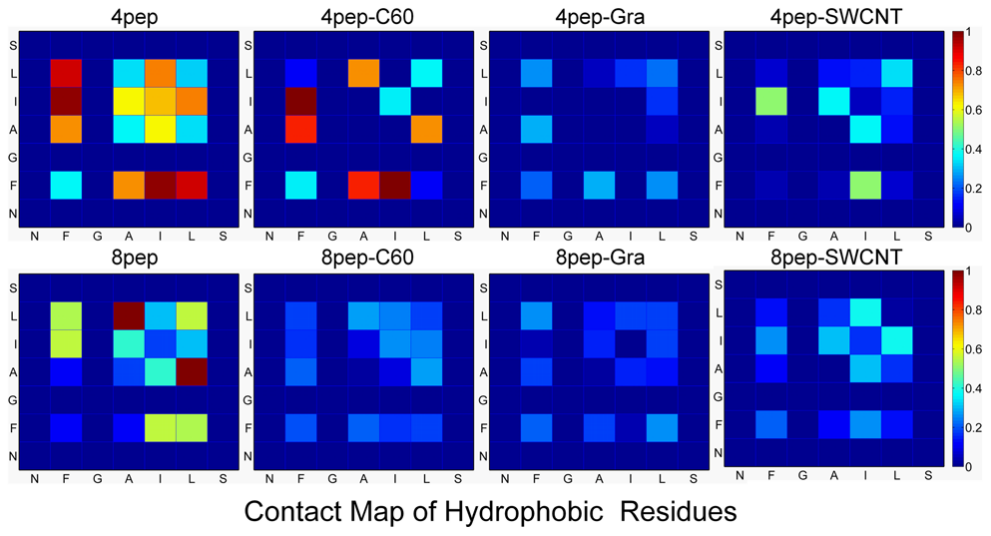
Contact map between the side chains of hydrophobic residues in different chains for each system. Only the last 50 ns trajectories are considered.

In addition, most residues of hIAPP_22–28_ are hydrophobic, and it is well established that the hydrophobic interaction is a major driving force for the β-sheet aggregation of NFGAIL (IAPP_22–27_) [47], [50], [52], [55]. Therefore, in order to confirm this point in IAPP_22–28_ aggragation, we investigated the side-chain contacts of hydrophobic residues between different chains. In the contact map of hydrophobic reisdues (Figure 10), the different color means the different contact probability during the last 50 ns simulation. For each system with 4 or 8 peptides, the largest contact number of 4 and 8 peptides without NPs is considered as 1.0 for reference, respectively. As can be seen, there are obvious contacts between hydrophobic residues in each system, especially F23 in systems with 4 peptides. Furthermore, in both two sets, the hydrophobic side-chains have the largest probability to contact with each other if there is no NP, which indicates that hydrophobic interactions play key roles in the oligomerization of hIAPP_22–28_ and the carbon NPs can weaken the hydrophobic interactions between peptides. In other words, as hydrophobic interactions can benefit hIAPP_22–28_ oligomerization, the NPs especially graphene and SWCNT can inhibit the aggregation of hIAPP_22–28_ peptides by blocking these beneficial interactions.

The results from our work show that the adsorption of graphene or SWCNT for peptides has a competitive advantage compared with the interaction between peptides. Furthermore, graphene and SWCNT can inhibit the formation of ordered β-sheet-rich oligomers of IAPP_22–28_ and make peptides prefer to adopt random structure, while due to its small size C60 has a much weaker effects. Therefore, the fibrillation of IAPP_22–28_ fragment may be inhibited at its early stage by graphene or SWCNT.

However, so far there exist two opposite opinions on the question―whether carbon nanoparticles accelerate or inhibit the formation of amyloid. Zheng et al. [56] support the view that the graphite surface can accelerate the aggregation of Aβ peptides into fibrils. Their results showed that hydrophobic graphite induced the quick adsorption of Aβ peptides regardless of their initial conformations and sizes, and Aβ peptides prefered to adopt random structure for monomers and remained β-rich-structure for small oligomers, but not helical structures. They also found that hydrophobic C-terminal residues of Aβ formed preferential interactions with the graphite surface to facilitate Aβ fibril formation and fibril growth. On the contrary, a recent experimental study [18] showed that graphene oxide strongly inhibited Aβ fibrillation by delaying the Aβ fibrillation process via adsorption of Aβ monomers. In addition, another research indicated carbon nanotube inhibited the formation of β-sheet-rich oligomers of the Aβ(16–22) peptide [16], and fullerene also strongly inhibited the Aβ peptide aggregation at the early stage, specifically the central hydrophobic motif, KLVFF, of Aβ(16–20) peptides [14]. These controversial conclusions suggest that further research needs to be performed to explore the effect of carbon nanoparticles on the formation of amyloid by considering the inherent structure of the studied peptides and different external conditions.

### The Surface Curvature and Area Play Significant Roles in the Interaction between Carbon Nanomaterials and IAPP_22–28_

From the above analysis, it can be seen that all the three kinds of carbon nanomaterials we investigated can reduce the content of β-sheet structure and affect the formation of β-sheet-rich IAPP_22–28_ oligomers in different degrees. In the following section, we will discuss the driving forces for that.

In this work, the simulated peptide is ACE-NFGAILS-NME, and the minimum distances between side chains of peptides and graphene almost all appear at 0.3 ns (Figure 8), especially hydrophobic ones including aromatic residue F23. This indicates graphene has a strong adsorption for IAPP_22–28_ and hydrophobic residues are easier to interact with graphene. F23 has large probabilities at around 0.3 nm for the graphene systems as Figure 8 shows. Consequently, the F23 residue in the peptide has a very strong interaction with graphitic material by the *π–π* stacking interaction. In accordance with this deduction, the Phe residue behaves like an “anchor”, which is “thrown” by the peptide to the graphene to lock itself onto the surface of graphene. Therefore, in Figure 9, the aromatic residue F23 with the largest peak values has a strong *π–π* stacking interaction with the graphene surface, and it may play an important role in the inhibition process. Interestingly, *π–π* stacking interactions don’t seem to be the dominant driving force in the interaction the IAPP_22–28_ peptides with SWCNT and C_60_. The *π–π* stacking interactions between the peptide and graphene, SWCNT, and C_60_ strongly depend on the probability of the aromatic residue F23 forming a stable and flat conformation with the nanomaterial surface. For the curved NPs such as SWCNT and C_60_, in our simulations, the lower probability of forming flat *π–π* stacking with the benzene ring of F23 reduced the role of *π–π* stacking in their overall binding affinity with peptides. Obviously, the total number of carbon atoms from the carbon NPs contacting with the F23 side chain decreases from graphene, to SWCNT, to C_60_.

In addition, most residues in IAPP_22–28_ peptide are hydrophobic, and the side chains of two middle ones, G24 and A25, are very small. The flexible hydrophobic aliphatic side chains can adapt to the curved carbon surfaces and form favorable interaction with SWCNT with smaller steric effects in the middle of peptide. Therefore, for SWCNT, the other hydrophobic residues with aliphatic side chain such as I26 and L27 also have a significant role as Figure 8 shows. In a recent work [16], Li et al also observed that carbon nanotube could inhibit the formation of β-sheet-rich oligomers of the Alzheimer’s amyloid-β(16–22) peptide through the hydrophobic and *π–π* stacking interactions.

However, the binding affinity of C_60_ for IAPP_22–28_ peptides is much lower, and both aromatic and other hydrophobic residues have smaller contribution than that in graphene and SWCNT systems. This may be due to the small size of C_60_, whose limited surface area makes it can only contact with a few residues and the contact numbers are nearly equal (about 100) in both systems (Figure 7).

It is well known that the surfaces of three kinds of carbon nanomaterials, graphene/SWCNT/C_60_, are hydrophobic. Then the hydrophobic residues of peptides should be much easier to be adsorbed than the hydrophilic ones. In our study, most residues in IAPP_22–28_ fragment are hydrophobic, so the interactions between these hydrophobic residues and NPs including hydrophobic interactions and *π–π* stacking interactions may be important for the inhibition of IAPP_22–28_ aggregation by weakening the hydrophobic interactions between peptides (Figure 10). It has been reported that the *π–π* stacking interactions between the aromatic residues and carbon-based NP play an important role in the interaction between proteins and the nanomaterials both from the results of simulation [57]–[61] and experiments [62], [63]. However, our results show that the three NPs have different hydrophobic and *π–π* stacking interactions, further lead to differing effects on the formation of β-sheet-rich oligomers. Obviously, the different surface curvatures of these carbon NPs may play a significant role in the different results, and the difference of surface areas is also an important factor. Therefore, although graphene, SWCNT, and C_60_ have similar chemical composition, the different surface curvature and area will affect their interaction with proteins or peptides, especially the interactions with aromatic residues.

### Conclusions

In this work, we simulated disordered tetramer and octamer of hIAPP_22–28_ without or with different carbon NPs including graphene/SWCNT/C_60_ to investigate the effects of these carbon nanomaterials on the aggregation behaviors of IAPP_22–28_. The obtained results indicate that IAPP_22–28_ peptides can be strongly adsorbed onto graphene and SWCNT. This adsorption interaction has competitive advantage over the aggregation ability between peptides. Hence, the presence of graphene or SWCNT can reduce the β-sheet content of peptides and inhibits the formation of the ordered β-sheets. As for C_60_, it prevents the aggregate in a small degree due to its small size and limited area. Our work suggests that the driving forces for the interaction between the studied carbon nanomaterials and peptide are both hydrophobic and *π–π* stacking interactions, and the surface curvature and area in these different graphitic nanomaterials are responsible for their different effects in the peptide aggregation. Overall, our findings provide significant insights into the inhibition mechanism of carbon nanomaterials (graphene, SWCNT and C_60_) against the aggregation of IAPP_22–28_ peptides. Our work is useful for further understanding the interaction between IAPP_22–28_ and carbon NPs, and suggests a potential role for these carbon NPs in the development of therapies against type II diabetes. MD simulation method can be regarded as an effective approach to explore the toxicity and safety of nanomaterials when they enter human body.

## Supporting Information

Figure S1 **The initial configuration of each system.** Each model is shown in two different viewpoints, and the periodic boundary is shown as a solid box in blue. The NPs and peptides are shown as sticks (green) and cartoon (white represents coil), respectively.
(TIF)

Table S1 Detailed information for the initial configuration of each system.
(PDF)

Text S1 Coordinates of C60.
(DOC)

Text S2 Coordinates of graphene interacting with 4 peptides.
(DOC)

Text S3 Coordinates of graphene interacting with 8 peptides.
(DOC)

Text S4 Coordinates of SWCNT.
(DOC)
