# Supplementary figures and images for: Exploring the Influence of Carbon Nanoparticles on the Formation of β-Sheet-Rich Oligomers of IAPP22–28 Peptide by Molecular Dynamics Simulation
Source: PLoS One. 2013 Jun 5;8(6):e65579. doi: 10.1371/journal.pone.0065579 (PMC3674003; doi:10.1371/journal.pone.0065579)

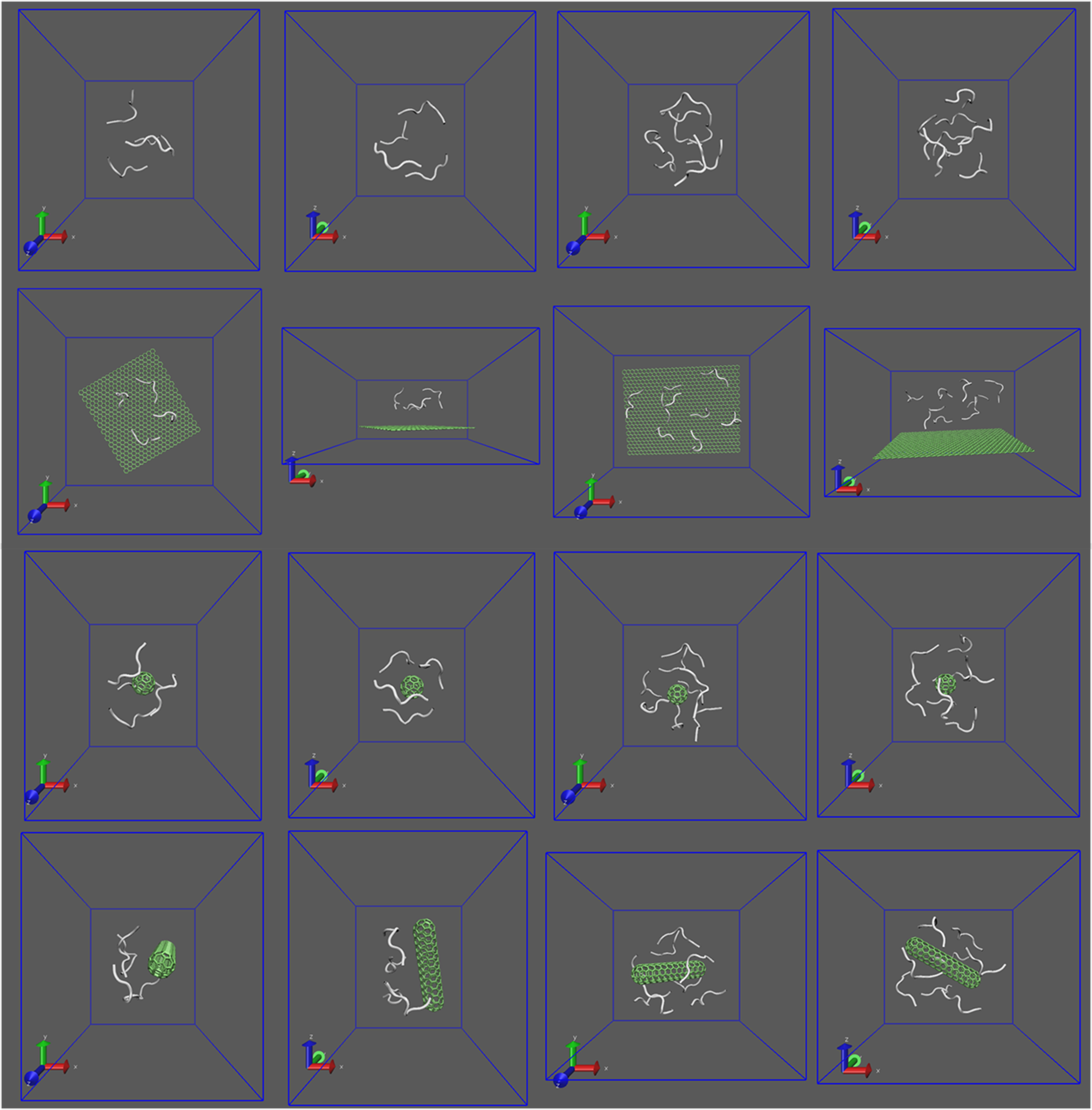

Supplement: Figure S1 — The initial configuration of each system. Each model is shown in two different viewpoints, and the periodic boundary is shown as a solid box in blue. The NPs and peptides are shown as sticks (green) and cartoon (white represents coil), respectively. (TIF) [file pone.0065579.s001.tif]
