# Supplementary material for: Exploring the Influence of Carbon Nanoparticles on the Formation of β-Sheet-Rich Oligomers of IAPP22–28 Peptide by Molecular Dynamics Simulation
Source: PLoS One. 2013 Jun 5;8(6):e65579. doi: 10.1371/journal.pone.0065579 (PMC3674003; doi:10.1371/journal.pone.0065579)
Supplement: Table S1 — Detailed information for the initial configuration of each system. (PDF) [file pone.0065579.s002.pdf]

Table S1. Detailed information for the initial configuration of each system.

System	Terms	4 peptides	8 peptides
Pep	PBC (Å^3)	51.04×55.23×53.00	52.03×52.87×55.97
	General view	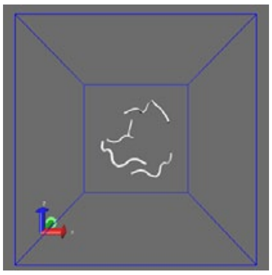	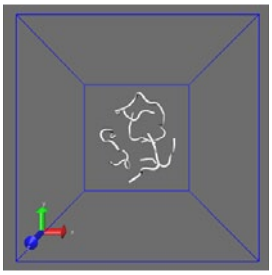
Pep+C_{60}	PBC (Å^3)	46.64×53.03×50.12	58.58×62.24×58.91
	General view	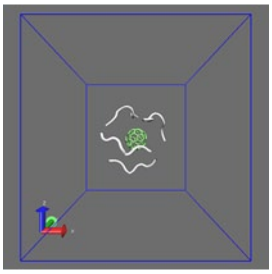	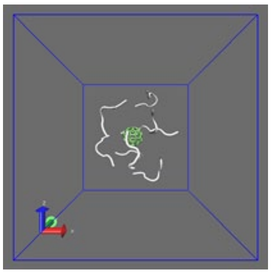
Pep+Gra	PBC (Å^3)	92.21×92.77×49.00	96.13×78.98×63.09
	General view	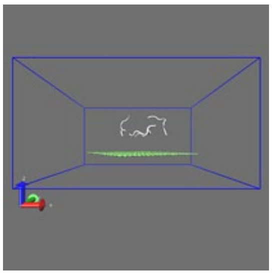	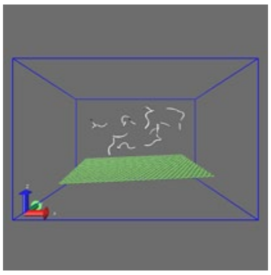
Pep+SWCNT	PBC (Å^3)	46.11×51.07×53.00	66.46×58.08×58.91
	General view	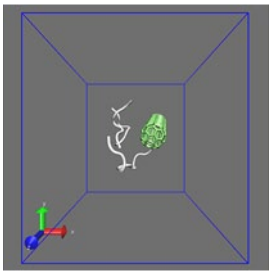	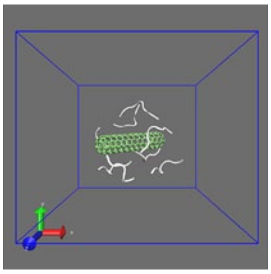
