# Supplementary material for: Exploring the Influence of Carbon Nanoparticles on the Formation of β-Sheet-Rich Oligomers of IAPP22–28 Peptide by Molecular Dynamics Simulation
Source: PLoS One. 2013 Jun 5;8(6):e65579. doi: 10.1371/journal.pone.0065579 (PMC3674003; doi:10.1371/journal.pone.0065579)
Supplement: Text S1 — Coordinates of C60. (DOC) [file pone.0065579.s003.doc]

**Text S1. Coordinates of C60.**

ATOM 1 C1 C60 1 0.000 0.000 0.000

ATOM 2 C2 C60 1 1.464 0.000 0.000

ATOM 3 C3 C60 1 1.916 1.392 0.000

ATOM 4 C4 C60 1 0.732 2.253 0.000

ATOM 5 C5 C60 1 -0.452 1.392 0.000

ATOM 6 C6 C60 1 -1.573 1.756 0.728

ATOM 7 C7 C60 1 -0.692 -0.953 0.728

ATOM 8 C8 C60 1 2.156 -0.953 0.728

ATOM 9 C9 C60 1 3.036 1.756 0.728

ATOM 10 C10 C60 1 0.732 3.431 0.728

ATOM 11 C11 C60 1 3.341 -0.568 1.498

ATOM 12 C12 C60 1 3.768 0.749 1.498

ATOM 13 C13 C60 1 3.037 3.002 1.498

ATOM 14 C14 C60 1 1.916 3.815 1.498

ATOM 15 C15 C60 1 -0.452 3.815 1.498

ATOM 16 C16 C60 1 -1.573 3.002 1.498

ATOM 17 C17 C60 1 -2.305 0.749 1.498

ATOM 18 C18 C60 1 -1.877 -0.568 1.498

ATOM 19 C19 C60 1 0.040 -1.960 1.498

ATOM 20 C20 C60 1 1.424 -1.960 1.498

ATOM 21 C21 C60 1 -0.692 -2.198 2.743

ATOM 22 C22 C60 1 -1.877 -1.338 2.743

ATOM 23 C23 C60 1 2.156 -2.198 2.743

ATOM 24 C24 C60 1 3.341 -1.338 2.743

ATOM 25 C25 C60 1 4.221 1.371 2.743

ATOM 26 C26 C60 1 3.768 2.764 2.743

ATOM 27 C27 C60 1 1.464 4.438 2.743

ATOM 28 C28 C60 1 0.000 4.438 2.743

ATOM 29 C29 C60 1 -2.305 2.764 2.743

ATOM 30 C30 C60 1 -2.757 1.371 2.743

ATOM 31 C31 C60 1 0.000 -2.423 3.921

ATOM 32 C32 C60 1 1.464 -2.423 3.921

ATOM 33 C33 C60 1 3.768 -0.749 3.921

ATOM 34 C34 C60 1 4.221 0.643 3.921

ATOM 35 C35 C60 1 3.341 3.353 3.921

ATOM 36 C36 C60 1 2.156 4.213 3.921

ATOM 1 C37 C60 1 -1.877 3.353 3.921

ATOM 38 C38 C60 1 -0.692 4.213 3.921

ATOM 39 C39 C60 1 -2.305 -0.749 3.921

ATOM 40 C40 C60 1 -2.757 0.643 3.921

ATOM 41 C41 C60 1 1.916 -1.801 5.166

ATOM 42 C42 C60 1 3.037 -0.987 5.166

ATOM 43 C43 C60 1 -1.573 -0.987 5.166

ATOM 44 C44 C60 1 -0.452 -1.801 5.166

ATOM 45 C45 C60 1 -2.305 1.266 5.166

ATOM 46 C46 C60 1 -1.877 2.583 5.166

ATOM 47 C47 C60 1 0.040 3.975 5.166

ATOM 48 C48 C60 1 1.424 3.975 5.166

ATOM 49 C49 C60 1 3.768 1.266 5.166

ATOM 50 C50 C60 1 3.341 2.583 5.166

ATOM 51 C51 C60 1 0.732 -1.416 5.936

ATOM 52 C52 C60 1 -1.573 0.259 5.936

ATOM 53 C53 C60 1 -0.692 2.968 5.936

ATOM 54 C54 C60 1 2.156 2.968 5.936

ATOM 55 C55 C60 1 3.036 0.259 5.936

ATOM 56 C56 C60 1 0.000 2.015 6.664

ATOM 57 C57 C60 1 1.464 2.015 6.664

ATOM 58 C58 C60 1 1.916 0.623 6.664

ATOM 59 C59 C60 1 0.732 -0.238 6.664

ATOM 60 C60 C60 1 -0.452 0.623 6.664

TER

END
