# Supplementary material for: Exploring the Influence of Carbon Nanoparticles on the Formation of β-Sheet-Rich Oligomers of IAPP22–28 Peptide by Molecular Dynamics Simulation
Source: PLoS One. 2013 Jun 5;8(6):e65579. doi: 10.1371/journal.pone.0065579 (PMC3674003; doi:10.1371/journal.pone.0065579)
Supplement: Text S2 — Coordinates of graphene interacting with 4 peptides. (DOC) [file pone.0065579.s004.doc]

**Text S2. Coordinates of graphene interacting with 4 peptides.**

ATOM 1 C1 GRA 1 0.097 49.108 3.481

ATOM 2 C2 GRA 1 4.358 41.728 3.481

ATOM 3 C3 GRA 1 4.358 46.648 3.481

ATOM 4 C4 GRA 1 4.358 51.568 3.481

ATOM 5 C5 GRA 1 8.618 34.348 3.481

ATOM 6 C6 GRA 1 8.618 39.268 3.481

ATOM 7 C7 GRA 1 8.618 44.188 3.481

ATOM 8 C8 GRA 1 8.618 49.108 3.481

ATOM 9 C9 GRA 1 8.618 54.028 3.481

ATOM 10 C10 GRA 1 12.879 26.968 3.481

ATOM 11 C11 GRA 1 12.879 31.888 3.481

ATOM 12 C12 GRA 1 12.879 36.808 3.481

ATOM 13 C13 GRA 1 12.879 41.728 3.481

ATOM 14 C14 GRA 1 12.879 46.648 3.481

ATOM 15 C15 GRA 1 12.879 51.568 3.481

ATOM 16 C16 GRA 1 12.879 56.488 3.481

ATOM 17 C17 GRA 1 17.140 19.588 3.481

ATOM 18 C18 GRA 1 17.140 24.508 3.481

ATOM 19 C19 GRA 1 17.140 29.428 3.481

ATOM 20 C20 GRA 1 17.140 34.348 3.481

ATOM 21 C21 GRA 1 17.140 39.268 3.481

ATOM 22 C22 GRA 1 17.140 44.188 3.481

ATOM 23 C23 GRA 1 17.140 49.108 3.481

ATOM 24 C24 GRA 1 17.140 54.028 3.481

ATOM 25 C25 GRA 1 17.140 58.948 3.481

ATOM 26 C26 GRA 1 21.401 12.208 3.481

ATOM 27 C27 GRA 1 21.401 17.128 3.481

ATOM 28 C28 GRA 1 21.401 22.048 3.481

ATOM 29 C29 GRA 1 21.401 26.968 3.481

ATOM 30 C30 GRA 1 21.401 31.888 3.481

ATOM 31 C31 GRA 1 21.401 36.808 3.481

ATOM 32 C32 GRA 1 21.401 41.728 3.481

ATOM 33 C33 GRA 1 21.401 46.648 3.481

ATOM 34 C34 GRA 1 21.401 51.568 3.481

ATOM 35 C35 GRA 1 21.401 56.488 3.481

ATOM 36 C36 GRA 1 21.401 61.408 3.481

ATOM 1 C37 GRA 1 25.662 4.828 3.481

ATOM 38 C38 GRA 1 25.662 9.748 3.481

ATOM 39 C39 GRA 1 25.662 14.668 3.481

ATOM 40 C40 GRA 1 25.662 19.588 3.481

ATOM 41 C41 GRA 1 25.662 24.508 3.481

ATOM 42 C42 GRA 1 25.662 29.428 3.481

ATOM 43 C43 GRA 1 25.662 34.348 3.481

ATOM 44 C44 GRA 1 25.662 39.268 3.481

ATOM 45 C45 GRA 1 25.662 44.188 3.481

ATOM 46 C46 GRA 1 25.662 49.108 3.481

ATOM 47 C47 GRA 1 25.662 54.028 3.481

ATOM 48 C48 GRA 1 25.662 58.948 3.481

ATOM 49 C49 GRA 1 25.662 63.868 3.481

ATOM 50 C50 GRA 1 29.923 7.288 3.481

ATOM 51 C51 GRA 1 29.923 12.208 3.481

ATOM 52 C52 GRA 1 29.923 17.128 3.481

ATOM 53 C53 GRA 1 29.923 22.048 3.481

ATOM 54 C54 GRA 1 29.923 26.968 3.481

ATOM 55 C55 GRA 1 29.923 31.888 3.481

ATOM 56 C56 GRA 1 29.923 36.808 3.481

ATOM 57 C57 GRA 1 29.923 41.728 3.481

ATOM 58 C58 GRA 1 29.923 46.648 3.481

ATOM 59 C59 GRA 1 29.923 51.568 3.481

ATOM 60 C60 GRA 1 29.923 56.488 3.481

ATOM 61 C61 GRA 1 29.923 61.408 3.481

ATOM 62 C62 GRA 1 29.923 66.328 3.481

ATOM 63 C63 GRA 1 34.183 9.748 3.481

ATOM 64 C64 GRA 1 34.183 14.668 3.481

ATOM 65 C65 GRA 1 34.183 19.588 3.481

ATOM 66 C66 GRA 1 34.183 24.508 3.481

ATOM 67 C67 GRA 1 34.183 29.428 3.481

ATOM 68 C68 GRA 1 34.183 34.348 3.481

ATOM 69 C69 GRA 1 34.183 39.268 3.481

ATOM 70 C70 GRA 1 34.183 44.188 3.481

ATOM 71 C71 GRA 1 34.183 49.108 3.481

ATOM 72 C72 GRA 1 34.183 54.028 3.481

ATOM 73 C73 GRA 1 34.183 58.948 3.481

ATOM 74 C74 GRA 1 34.183 63.868 3.481

ATOM 75 C75 GRA 1 34.183 68.788 3.481

ATOM 76 C76 GRA 1 38.444 12.208 3.481

ATOM 77 C77 GRA 1 38.444 17.128 3.481

ATOM 78 C78 GRA 1 38.444 22.048 3.481

ATOM 79 C79 GRA 1 38.444 26.968 3.481

ATOM 80 C80 GRA 1 38.444 31.888 3.481

ATOM 81 C81 GRA 1 38.444 36.808 3.481

ATOM 82 C82 GRA 1 38.444 41.728 3.481

ATOM 83 C83 GRA 1 38.444 46.648 3.481

ATOM 84 C84 GRA 1 38.444 51.568 3.481

ATOM 85 C85 GRA 1 38.444 56.488 3.481

ATOM 86 C86 GRA 1 38.444 61.408 3.481

ATOM 87 C87 GRA 1 38.444 66.328 3.481

ATOM 88 C88 GRA 1 38.444 71.248 3.481

ATOM 89 C89 GRA 1 42.705 14.668 3.481

ATOM 90 C90 GRA 1 42.705 19.588 3.481

ATOM 91 C91 GRA 1 42.705 24.508 3.481

ATOM 92 C92 GRA 1 42.705 29.428 3.481

ATOM 93 C93 GRA 1 42.705 34.348 3.481

ATOM 94 C94 GRA 1 42.705 39.268 3.481

ATOM 95 C95 GRA 1 42.705 44.188 3.481

ATOM 96 C96 GRA 1 42.705 49.108 3.481

ATOM 97 C97 GRA 1 42.705 54.028 3.481

ATOM 98 C98 GRA 1 42.705 58.948 3.481

ATOM 99 C99 GRA 1 42.705 63.868 3.481

ATOM 100 0C10 GRA 1 42.705 68.788 3.481

ATOM 101 1C10 GRA 1 42.705 73.708 3.481

ATOM 102 2C10 GRA 1 46.966 17.128 3.481

ATOM 103 3C10 GRA 1 46.966 22.048 3.481

ATOM 104 4C10 GRA 1 46.966 26.968 3.481

ATOM 105 5C10 GRA 1 46.966 31.888 3.481

ATOM 106 6C10 GRA 1 46.966 36.808 3.481

ATOM 107 7C10 GRA 1 46.966 41.728 3.481

ATOM 108 8C10 GRA 1 46.966 46.648 3.481

ATOM 109 9C10 GRA 1 46.966 51.568 3.481

ATOM 110 0C11 GRA 1 46.966 56.488 3.481

ATOM 111 1C11 GRA 1 46.966 61.408 3.481

ATOM 112 2C11 GRA 1 46.966 66.328 3.481

ATOM 113 3C11 GRA 1 51.227 19.588 3.481

ATOM 114 4C11 GRA 1 51.227 24.508 3.481

ATOM 115 5C11 GRA 1 51.227 29.428 3.481

ATOM 116 6C11 GRA 1 51.227 34.348 3.481

ATOM 117 7C11 GRA 1 51.227 39.268 3.481

ATOM 118 8C11 GRA 1 51.227 44.188 3.481

ATOM 119 9C11 GRA 1 51.227 49.108 3.481

ATOM 120 0C12 GRA 1 51.227 54.028 3.481

ATOM 121 1C12 GRA 1 51.227 58.948 3.481

ATOM 122 2C12 GRA 1 55.488 22.048 3.481

ATOM 123 3C12 GRA 1 55.488 26.968 3.481

ATOM 124 4C12 GRA 1 55.488 31.888 3.481

ATOM 125 5C12 GRA 1 55.488 36.808 3.481

ATOM 126 6C12 GRA 1 55.488 41.728 3.481

ATOM 127 7C12 GRA 1 55.488 46.648 3.481

ATOM 128 8C12 GRA 1 55.488 51.568 3.481

ATOM 129 9C12 GRA 1 59.749 24.508 3.481

ATOM 130 0C13 GRA 1 59.749 29.428 3.481

ATOM 131 1C13 GRA 1 59.749 34.348 3.481

ATOM 132 2C13 GRA 1 59.749 39.268 3.481

ATOM 133 3C13 GRA 1 59.749 44.188 3.481

ATOM 134 4C13 GRA 1 64.009 26.968 3.481

ATOM 135 5C13 GRA 1 64.009 31.888 3.481

ATOM 136 6C13 GRA 1 64.009 36.808 3.481

ATOM 137 7C13 GRA 1 68.270 29.428 3.481

ATOM 138 8C13 GRA 1 4.358 44.188 3.481

ATOM 139 9C13 GRA 1 4.358 49.108 3.481

ATOM 140 0C14 GRA 1 8.618 36.808 3.481

ATOM 141 1C14 GRA 1 8.618 41.728 3.481

ATOM 142 2C14 GRA 1 8.618 46.648 3.481

ATOM 143 3C14 GRA 1 8.618 51.568 3.481

ATOM 144 4C14 GRA 1 12.879 29.428 3.481

ATOM 145 5C14 GRA 1 12.879 34.348 3.481

ATOM 146 6C14 GRA 1 12.879 39.268 3.481

ATOM 147 7C14 GRA 1 12.879 44.188 3.481

ATOM 148 8C14 GRA 1 12.879 49.108 3.481

ATOM 149 9C14 GRA 1 12.879 54.028 3.481

ATOM 150 0C15 GRA 1 17.140 22.048 3.481

ATOM 151 1C15 GRA 1 17.140 26.968 3.481

ATOM 152 2C15 GRA 1 17.140 31.888 3.481

ATOM 153 3C15 GRA 1 17.140 36.808 3.481

ATOM 154 4C15 GRA 1 17.140 41.728 3.481

ATOM 155 5C15 GRA 1 17.140 46.648 3.481

ATOM 156 6C15 GRA 1 17.140 51.568 3.481

ATOM 157 7C15 GRA 1 17.140 56.488 3.481

ATOM 158 8C15 GRA 1 21.401 14.668 3.481

ATOM 159 9C15 GRA 1 21.401 19.588 3.481

ATOM 160 0C16 GRA 1 21.401 24.508 3.481

ATOM 161 1C16 GRA 1 21.401 29.428 3.481

ATOM 162 2C16 GRA 1 21.401 34.348 3.481

ATOM 163 3C16 GRA 1 21.401 39.268 3.481

ATOM 164 4C16 GRA 1 21.401 44.188 3.481

ATOM 165 5C16 GRA 1 21.401 49.108 3.481

ATOM 166 6C16 GRA 1 21.401 54.028 3.481

ATOM 167 7C16 GRA 1 21.401 58.948 3.481

ATOM 168 8C16 GRA 1 25.662 7.288 3.481

ATOM 169 9C16 GRA 1 25.662 12.208 3.481

ATOM 170 0C17 GRA 1 25.662 17.128 3.481

ATOM 171 1C17 GRA 1 25.662 22.048 3.481

ATOM 172 2C17 GRA 1 25.662 26.968 3.481

ATOM 173 3C17 GRA 1 25.662 31.888 3.481

ATOM 174 4C17 GRA 1 25.662 36.808 3.481

ATOM 175 5C17 GRA 1 25.662 41.728 3.481

ATOM 176 6C17 GRA 1 25.662 46.648 3.481

ATOM 177 7C17 GRA 1 25.662 51.568 3.481

ATOM 178 8C17 GRA 1 25.662 56.488 3.481

ATOM 179 9C17 GRA 1 25.662 61.408 3.481

ATOM 180 0C18 GRA 1 29.923 4.828 3.481

ATOM 181 1C18 GRA 1 29.923 9.748 3.481

ATOM 182 2C18 GRA 1 29.923 14.668 3.481

ATOM 183 3C18 GRA 1 29.923 19.588 3.481

ATOM 184 4C18 GRA 1 29.923 24.508 3.481

ATOM 185 5C18 GRA 1 29.923 29.428 3.481

ATOM 186 6C18 GRA 1 29.923 34.348 3.481

ATOM 187 7C18 GRA 1 29.923 39.268 3.481

ATOM 188 8C18 GRA 1 29.923 44.188 3.481

ATOM 189 9C18 GRA 1 29.923 49.108 3.481

ATOM 190 0C19 GRA 1 29.923 54.028 3.481

ATOM 191 1C19 GRA 1 29.923 58.948 3.481

ATOM 192 2C19 GRA 1 29.923 63.868 3.481

ATOM 193 3C19 GRA 1 34.183 7.288 3.481

ATOM 194 4C19 GRA 1 34.183 12.208 3.481

ATOM 195 5C19 GRA 1 34.183 17.128 3.481

ATOM 196 6C19 GRA 1 34.183 22.048 3.481

ATOM 197 7C19 GRA 1 34.183 26.968 3.481

ATOM 198 8C19 GRA 1 34.183 31.888 3.481

ATOM 199 9C19 GRA 1 34.183 36.808 3.481

ATOM 200 0C20 GRA 1 34.183 41.728 3.481

ATOM 201 1C20 GRA 1 34.183 46.648 3.481

ATOM 202 2C20 GRA 1 34.183 51.568 3.481

ATOM 203 3C20 GRA 1 34.183 56.488 3.481

ATOM 204 4C20 GRA 1 34.183 61.408 3.481

ATOM 205 5C20 GRA 1 34.183 66.328 3.481

ATOM 206 6C20 GRA 1 38.444 9.748 3.481

ATOM 207 7C20 GRA 1 38.444 14.668 3.481

ATOM 208 8C20 GRA 1 38.444 19.588 3.481

ATOM 209 9C20 GRA 1 38.444 24.508 3.481

ATOM 210 0C21 GRA 1 38.444 29.428 3.481

ATOM 211 1C21 GRA 1 38.444 34.348 3.481

ATOM 212 2C21 GRA 1 38.444 39.268 3.481

ATOM 213 3C21 GRA 1 38.444 44.188 3.481

ATOM 214 4C21 GRA 1 38.444 49.108 3.481

ATOM 215 5C21 GRA 1 38.444 54.028 3.481

ATOM 216 6C21 GRA 1 38.444 58.948 3.481

ATOM 217 7C21 GRA 1 38.444 63.868 3.481

ATOM 218 8C21 GRA 1 38.444 68.788 3.481

ATOM 219 9C21 GRA 1 42.705 12.208 3.481

ATOM 220 0C22 GRA 1 42.705 17.128 3.481

ATOM 221 1C22 GRA 1 42.705 22.048 3.481

ATOM 222 2C22 GRA 1 42.705 26.968 3.481

ATOM 223 3C22 GRA 1 42.705 31.888 3.481

ATOM 224 4C22 GRA 1 42.705 36.808 3.481

ATOM 225 5C22 GRA 1 42.705 41.728 3.481

ATOM 226 6C22 GRA 1 42.705 46.648 3.481

ATOM 227 7C22 GRA 1 42.705 51.568 3.481

ATOM 228 8C22 GRA 1 42.705 56.488 3.481

ATOM 229 9C22 GRA 1 42.705 61.408 3.481

ATOM 230 0C23 GRA 1 42.705 66.328 3.481

ATOM 231 1C23 GRA 1 42.705 71.248 3.481

ATOM 232 2C23 GRA 1 46.966 14.668 3.481

ATOM 233 3C23 GRA 1 46.966 19.588 3.481

ATOM 234 4C23 GRA 1 46.966 24.508 3.481

ATOM 235 5C23 GRA 1 46.966 29.428 3.481

ATOM 236 6C23 GRA 1 46.966 34.348 3.481

ATOM 237 7C23 GRA 1 46.966 39.268 3.481

ATOM 238 8C23 GRA 1 46.966 44.188 3.481

ATOM 239 9C23 GRA 1 46.966 49.108 3.481

ATOM 240 0C24 GRA 1 46.966 54.028 3.481

ATOM 241 1C24 GRA 1 46.966 58.948 3.481

ATOM 242 2C24 GRA 1 46.966 63.868 3.481

ATOM 243 3C24 GRA 1 51.227 17.128 3.481

ATOM 244 4C24 GRA 1 51.227 22.048 3.481

ATOM 245 5C24 GRA 1 51.227 26.968 3.481

ATOM 246 6C24 GRA 1 51.227 31.888 3.481

ATOM 247 7C24 GRA 1 51.227 36.808 3.481

ATOM 248 8C24 GRA 1 51.227 41.728 3.481

ATOM 249 9C24 GRA 1 51.227 46.648 3.481

ATOM 250 0C25 GRA 1 51.227 51.568 3.481

ATOM 251 1C25 GRA 1 51.227 56.488 3.481

ATOM 252 2C25 GRA 1 55.488 19.588 3.481

ATOM 253 3C25 GRA 1 55.488 24.508 3.481

ATOM 254 4C25 GRA 1 55.488 29.428 3.481

ATOM 255 5C25 GRA 1 55.488 34.348 3.481

ATOM 256 6C25 GRA 1 55.488 39.268 3.481

ATOM 257 7C25 GRA 1 55.488 44.188 3.481

ATOM 258 8C25 GRA 1 55.488 49.108 3.481

ATOM 259 9C25 GRA 1 59.749 22.048 3.481

ATOM 260 0C26 GRA 1 59.749 26.968 3.481

ATOM 261 1C26 GRA 1 59.749 31.888 3.481

ATOM 262 2C26 GRA 1 59.749 36.808 3.481

ATOM 263 3C26 GRA 1 59.749 41.728 3.481

ATOM 264 4C26 GRA 1 64.009 24.508 3.481

ATOM 265 5C26 GRA 1 64.009 29.428 3.481

ATOM 266 6C26 GRA 1 64.009 34.348 3.481

ATOM 267 7C26 GRA 1 68.270 26.968 3.481

ATOM 268 8C26 GRA 1 2.227 47.878 3.481

ATOM 269 9C26 GRA 1 6.488 40.498 3.481

ATOM 270 0C27 GRA 1 6.488 45.418 3.481

ATOM 271 1C27 GRA 1 6.488 50.338 3.481

ATOM 272 2C27 GRA 1 10.749 33.118 3.481

ATOM 273 3C27 GRA 1 10.749 38.038 3.481

ATOM 274 4C27 GRA 1 10.749 42.958 3.481

ATOM 275 5C27 GRA 1 10.749 47.878 3.481

ATOM 276 6C27 GRA 1 10.749 52.798 3.481

ATOM 277 7C27 GRA 1 15.010 25.738 3.481

ATOM 278 8C27 GRA 1 15.010 30.658 3.481

ATOM 279 9C27 GRA 1 15.010 35.578 3.481

ATOM 280 0C28 GRA 1 15.010 40.498 3.481

ATOM 281 1C28 GRA 1 15.010 45.418 3.481

ATOM 282 2C28 GRA 1 15.010 50.338 3.481

ATOM 283 3C28 GRA 1 15.010 55.258 3.481

ATOM 284 4C28 GRA 1 19.271 18.358 3.481

ATOM 285 5C28 GRA 1 19.271 23.278 3.481

ATOM 286 6C28 GRA 1 19.271 28.198 3.481

ATOM 287 7C28 GRA 1 19.271 33.118 3.481

ATOM 288 8C28 GRA 1 19.271 38.038 3.481

ATOM 289 9C28 GRA 1 19.271 42.958 3.481

ATOM 290 0C29 GRA 1 19.271 47.878 3.481

ATOM 291 1C29 GRA 1 19.271 52.798 3.481

ATOM 292 2C29 GRA 1 19.271 57.718 3.481

ATOM 293 3C29 GRA 1 23.531 10.978 3.481

ATOM 294 4C29 GRA 1 23.531 15.898 3.481

ATOM 295 5C29 GRA 1 23.531 20.818 3.481

ATOM 296 6C29 GRA 1 23.531 25.738 3.481

ATOM 297 7C29 GRA 1 23.531 30.658 3.481

ATOM 298 8C29 GRA 1 23.531 35.578 3.481

ATOM 299 9C29 GRA 1 23.531 40.498 3.481

ATOM 300 0C30 GRA 1 23.531 45.418 3.481

ATOM 301 1C30 GRA 1 23.531 50.338 3.481

ATOM 302 2C30 GRA 1 23.531 55.258 3.481

ATOM 303 3C30 GRA 1 23.531 60.178 3.481

ATOM 304 4C30 GRA 1 27.792 3.598 3.481

ATOM 305 5C30 GRA 1 27.792 8.518 3.481

ATOM 306 6C30 GRA 1 27.792 13.438 3.481

ATOM 307 7C30 GRA 1 27.792 18.358 3.481

ATOM 308 8C30 GRA 1 27.792 23.278 3.481

ATOM 309 9C30 GRA 1 27.792 28.198 3.481

ATOM 310 0C31 GRA 1 27.792 33.118 3.481

ATOM 311 1C31 GRA 1 27.792 38.038 3.481

ATOM 312 2C31 GRA 1 27.792 42.958 3.481

ATOM 313 3C31 GRA 1 27.792 47.878 3.481

ATOM 314 4C31 GRA 1 27.792 52.798 3.481

ATOM 315 5C31 GRA 1 27.792 57.718 3.481

ATOM 316 6C31 GRA 1 27.792 62.638 3.481

ATOM 317 7C31 GRA 1 32.053 6.058 3.481

ATOM 318 8C31 GRA 1 32.053 10.978 3.481

ATOM 319 9C31 GRA 1 32.053 15.898 3.481

ATOM 320 0C32 GRA 1 32.053 20.818 3.481

ATOM 321 1C32 GRA 1 32.053 25.738 3.481

ATOM 322 2C32 GRA 1 32.053 30.658 3.481

ATOM 323 3C32 GRA 1 32.053 35.578 3.481

ATOM 324 4C32 GRA 1 32.053 40.498 3.481

ATOM 325 5C32 GRA 1 32.053 45.418 3.481

ATOM 326 6C32 GRA 1 32.053 50.338 3.481

ATOM 327 7C32 GRA 1 32.053 55.258 3.481

ATOM 328 8C32 GRA 1 32.053 60.178 3.481

ATOM 329 9C32 GRA 1 32.053 65.098 3.481

ATOM 330 0C33 GRA 1 36.314 8.518 3.481

ATOM 331 1C33 GRA 1 36.314 13.438 3.481

ATOM 332 2C33 GRA 1 36.314 18.358 3.481

ATOM 333 3C33 GRA 1 36.314 23.278 3.481

ATOM 334 4C33 GRA 1 36.314 28.198 3.481

ATOM 335 5C33 GRA 1 36.314 33.118 3.481

ATOM 336 6C33 GRA 1 36.314 38.038 3.481

ATOM 337 7C33 GRA 1 36.314 42.958 3.481

ATOM 338 8C33 GRA 1 36.314 47.878 3.481

ATOM 339 9C33 GRA 1 36.314 52.798 3.481

ATOM 340 0C34 GRA 1 36.314 57.718 3.481

ATOM 341 1C34 GRA 1 36.314 62.638 3.481

ATOM 342 2C34 GRA 1 36.314 67.558 3.481

ATOM 343 3C34 GRA 1 40.575 10.978 3.481

ATOM 344 4C34 GRA 1 40.575 15.898 3.481

ATOM 345 5C34 GRA 1 40.575 20.818 3.481

ATOM 346 6C34 GRA 1 40.575 25.738 3.481

ATOM 347 7C34 GRA 1 40.575 30.658 3.481

ATOM 348 8C34 GRA 1 40.575 35.578 3.481

ATOM 349 9C34 GRA 1 40.575 40.498 3.481

ATOM 350 0C35 GRA 1 40.575 45.418 3.481

ATOM 351 1C35 GRA 1 40.575 50.338 3.481

ATOM 352 2C35 GRA 1 40.575 55.258 3.481

ATOM 353 3C35 GRA 1 40.575 60.178 3.481

ATOM 354 4C35 GRA 1 40.575 65.098 3.481

ATOM 355 5C35 GRA 1 40.575 70.018 3.481

ATOM 356 6C35 GRA 1 44.836 13.438 3.481

ATOM 357 7C35 GRA 1 44.836 18.358 3.481

ATOM 358 8C35 GRA 1 44.836 23.278 3.481

ATOM 359 9C35 GRA 1 44.836 28.198 3.481

ATOM 360 0C36 GRA 1 44.836 33.118 3.481

ATOM 361 1C36 GRA 1 44.836 38.038 3.481

ATOM 362 2C36 GRA 1 44.836 42.958 3.481

ATOM 363 3C36 GRA 1 44.836 47.878 3.481

ATOM 364 4C36 GRA 1 44.836 52.798 3.481

ATOM 365 5C36 GRA 1 44.836 57.718 3.481

ATOM 366 6C36 GRA 1 44.836 62.638 3.481

ATOM 367 7C36 GRA 1 44.836 67.558 3.481

ATOM 368 8C36 GRA 1 49.096 15.898 3.481

ATOM 369 9C36 GRA 1 49.096 20.818 3.481

ATOM 370 0C37 GRA 1 49.096 25.738 3.481

ATOM 371 1C37 GRA 1 49.096 30.658 3.481

ATOM 372 2C37 GRA 1 49.096 35.578 3.481

ATOM 373 3C37 GRA 1 49.096 40.498 3.481

ATOM 374 4C37 GRA 1 49.096 45.418 3.481

ATOM 375 5C37 GRA 1 49.096 50.338 3.481

ATOM 376 6C37 GRA 1 49.096 55.258 3.481

ATOM 377 7C37 GRA 1 49.096 60.178 3.481

ATOM 378 8C37 GRA 1 53.357 18.358 3.481

ATOM 379 9C37 GRA 1 53.357 23.278 3.481

ATOM 380 0C38 GRA 1 53.357 28.198 3.481

ATOM 381 1C38 GRA 1 53.357 33.118 3.481

ATOM 382 2C38 GRA 1 53.357 38.038 3.481

ATOM 383 3C38 GRA 1 53.357 42.958 3.481

ATOM 384 4C38 GRA 1 53.357 47.878 3.481

ATOM 385 5C38 GRA 1 53.357 52.798 3.481

ATOM 386 6C38 GRA 1 57.618 20.818 3.481

ATOM 387 7C38 GRA 1 57.618 25.738 3.481

ATOM 388 8C38 GRA 1 57.618 30.658 3.481

ATOM 389 9C38 GRA 1 57.618 35.578 3.481

ATOM 390 0C39 GRA 1 57.618 40.498 3.481

ATOM 391 1C39 GRA 1 57.618 45.418 3.481

ATOM 392 2C39 GRA 1 61.879 23.278 3.481

ATOM 393 3C39 GRA 1 61.879 28.198 3.481

ATOM 394 4C39 GRA 1 61.879 33.118 3.481

ATOM 395 5C39 GRA 1 61.879 38.038 3.481

ATOM 396 6C39 GRA 1 66.140 25.738 3.481

ATOM 397 7C39 GRA 1 66.140 30.658 3.481

ATOM 398 8C39 GRA 1 2.227 45.418 3.481

ATOM 399 9C39 GRA 1 2.227 50.338 3.481

ATOM 400 0C40 GRA 1 6.488 38.038 3.481

ATOM 401 1C40 GRA 1 6.488 42.958 3.481

ATOM 402 2C40 GRA 1 6.488 47.878 3.481

ATOM 403 3C40 GRA 1 6.488 52.798 3.481

ATOM 404 4C40 GRA 1 10.749 30.658 3.481

ATOM 405 5C40 GRA 1 10.749 35.578 3.481

ATOM 406 6C40 GRA 1 10.749 40.498 3.481

ATOM 407 7C40 GRA 1 10.749 45.418 3.481

ATOM 408 8C40 GRA 1 10.749 50.338 3.481

ATOM 409 9C40 GRA 1 10.749 55.258 3.481

ATOM 410 0C41 GRA 1 15.010 23.278 3.481

ATOM 411 1C41 GRA 1 15.010 28.198 3.481

ATOM 412 2C41 GRA 1 15.010 33.118 3.481

ATOM 413 3C41 GRA 1 15.010 38.038 3.481

ATOM 414 4C41 GRA 1 15.010 42.958 3.481

ATOM 415 5C41 GRA 1 15.010 47.878 3.481

ATOM 416 6C41 GRA 1 15.010 52.798 3.481

ATOM 417 7C41 GRA 1 15.010 57.718 3.481

ATOM 418 8C41 GRA 1 19.271 15.898 3.481

ATOM 419 9C41 GRA 1 19.271 20.818 3.481

ATOM 420 0C42 GRA 1 19.271 25.738 3.481

ATOM 421 1C42 GRA 1 19.271 30.658 3.481

ATOM 422 2C42 GRA 1 19.271 35.578 3.481

ATOM 423 3C42 GRA 1 19.271 40.498 3.481

ATOM 424 4C42 GRA 1 19.271 45.418 3.481

ATOM 425 5C42 GRA 1 19.271 50.338 3.481

ATOM 426 6C42 GRA 1 19.271 55.258 3.481

ATOM 427 7C42 GRA 1 19.271 60.178 3.481

ATOM 428 8C42 GRA 1 23.531 8.518 3.481

ATOM 429 9C42 GRA 1 23.531 13.438 3.481

ATOM 430 0C43 GRA 1 23.531 18.358 3.481

ATOM 431 1C43 GRA 1 23.531 23.278 3.481

ATOM 432 2C43 GRA 1 23.531 28.198 3.481

ATOM 433 3C43 GRA 1 23.531 33.118 3.481

ATOM 434 4C43 GRA 1 23.531 38.038 3.481

ATOM 435 5C43 GRA 1 23.531 42.958 3.481

ATOM 436 6C43 GRA 1 23.531 47.878 3.481

ATOM 437 7C43 GRA 1 23.531 52.798 3.481

ATOM 438 8C43 GRA 1 23.531 57.718 3.481

ATOM 439 9C43 GRA 1 23.531 62.638 3.481

ATOM 440 0C44 GRA 1 27.792 6.058 3.481

ATOM 441 1C44 GRA 1 27.792 10.978 3.481

ATOM 442 2C44 GRA 1 27.792 15.898 3.481

ATOM 443 3C44 GRA 1 27.792 20.818 3.481

ATOM 444 4C44 GRA 1 27.792 25.738 3.481

ATOM 445 5C44 GRA 1 27.792 30.658 3.481

ATOM 446 6C44 GRA 1 27.792 35.578 3.481

ATOM 447 7C44 GRA 1 27.792 40.498 3.481

ATOM 448 8C44 GRA 1 27.792 45.418 3.481

ATOM 449 9C44 GRA 1 27.792 50.338 3.481

ATOM 450 0C45 GRA 1 27.792 55.258 3.481

ATOM 451 1C45 GRA 1 27.792 60.178 3.481

ATOM 452 2C45 GRA 1 27.792 65.098 3.481

ATOM 453 3C45 GRA 1 32.053 8.518 3.481

ATOM 454 4C45 GRA 1 32.053 13.438 3.481

ATOM 455 5C45 GRA 1 32.053 18.358 3.481

ATOM 456 6C45 GRA 1 32.053 23.278 3.481

ATOM 457 7C45 GRA 1 32.053 28.198 3.481

ATOM 458 8C45 GRA 1 32.053 33.118 3.481

ATOM 459 9C45 GRA 1 32.053 38.038 3.481

ATOM 460 0C46 GRA 1 32.053 42.958 3.481

ATOM 461 1C46 GRA 1 32.053 47.878 3.481

ATOM 462 2C46 GRA 1 32.053 52.798 3.481

ATOM 463 3C46 GRA 1 32.053 57.718 3.481

ATOM 464 4C46 GRA 1 32.053 62.638 3.481

ATOM 465 5C46 GRA 1 32.053 67.558 3.481

ATOM 466 6C46 GRA 1 36.314 10.978 3.481

ATOM 467 7C46 GRA 1 36.314 15.898 3.481

ATOM 468 8C46 GRA 1 36.314 20.818 3.481

ATOM 469 9C46 GRA 1 36.314 25.738 3.481

ATOM 470 0C47 GRA 1 36.314 30.658 3.481

ATOM 471 1C47 GRA 1 36.314 35.578 3.481

ATOM 472 2C47 GRA 1 36.314 40.498 3.481

ATOM 473 3C47 GRA 1 36.314 45.418 3.481

ATOM 474 4C47 GRA 1 36.314 50.338 3.481

ATOM 475 5C47 GRA 1 36.314 55.258 3.481

ATOM 476 6C47 GRA 1 36.314 60.178 3.481

ATOM 477 7C47 GRA 1 36.314 65.098 3.481

ATOM 478 8C47 GRA 1 36.314 70.018 3.481

ATOM 479 9C47 GRA 1 40.575 13.438 3.481

ATOM 480 0C48 GRA 1 40.575 18.358 3.481

ATOM 481 1C48 GRA 1 40.575 23.278 3.481

ATOM 482 2C48 GRA 1 40.575 28.198 3.481

ATOM 483 3C48 GRA 1 40.575 33.118 3.481

ATOM 484 4C48 GRA 1 40.575 38.038 3.481

ATOM 485 5C48 GRA 1 40.575 42.958 3.481

ATOM 486 6C48 GRA 1 40.575 47.878 3.481

ATOM 487 7C48 GRA 1 40.575 52.798 3.481

ATOM 488 8C48 GRA 1 40.575 57.718 3.481

ATOM 489 9C48 GRA 1 40.575 62.638 3.481

ATOM 490 0C49 GRA 1 40.575 67.558 3.481

ATOM 491 1C49 GRA 1 40.575 72.478 3.481

ATOM 492 2C49 GRA 1 44.836 15.898 3.481

ATOM 493 3C49 GRA 1 44.836 20.818 3.481

ATOM 494 4C49 GRA 1 44.836 25.738 3.481

ATOM 495 5C49 GRA 1 44.836 30.658 3.481

ATOM 496 6C49 GRA 1 44.836 35.578 3.481

ATOM 497 7C49 GRA 1 44.836 40.498 3.481

ATOM 498 8C49 GRA 1 44.836 45.418 3.481

ATOM 499 9C49 GRA 1 44.836 50.338 3.481

ATOM 500 0C50 GRA 1 44.836 55.258 3.481

ATOM 501 1C50 GRA 1 44.836 60.178 3.481

ATOM 502 2C50 GRA 1 44.836 65.098 3.481

ATOM 503 3C50 GRA 1 44.836 70.018 3.481

ATOM 504 4C50 GRA 1 49.096 18.358 3.481

ATOM 505 5C50 GRA 1 49.096 23.278 3.481

ATOM 506 6C50 GRA 1 49.096 28.198 3.481

ATOM 507 7C50 GRA 1 49.096 33.118 3.481

ATOM 508 8C50 GRA 1 49.096 38.038 3.481

ATOM 509 9C50 GRA 1 49.096 42.958 3.481

ATOM 510 0C51 GRA 1 49.096 47.878 3.481

ATOM 511 1C51 GRA 1 49.096 52.798 3.481

ATOM 512 2C51 GRA 1 49.096 57.718 3.481

ATOM 513 3C51 GRA 1 49.096 62.638 3.481

ATOM 514 4C51 GRA 1 53.357 20.818 3.481

ATOM 515 5C51 GRA 1 53.357 25.738 3.481

ATOM 516 6C51 GRA 1 53.357 30.658 3.481

ATOM 517 7C51 GRA 1 53.357 35.578 3.481

ATOM 518 8C51 GRA 1 53.357 40.498 3.481

ATOM 519 9C51 GRA 1 53.357 45.418 3.481

ATOM 520 0C52 GRA 1 53.357 50.338 3.481

ATOM 521 1C52 GRA 1 53.357 55.258 3.481

ATOM 522 2C52 GRA 1 57.618 23.278 3.481

ATOM 523 3C52 GRA 1 57.618 28.198 3.481

ATOM 524 4C52 GRA 1 57.618 33.118 3.481

ATOM 525 5C52 GRA 1 57.618 38.038 3.481

ATOM 526 6C52 GRA 1 57.618 42.958 3.481

ATOM 527 7C52 GRA 1 57.618 47.878 3.481

ATOM 528 8C52 GRA 1 61.879 25.738 3.481

ATOM 529 9C52 GRA 1 61.879 30.658 3.481

ATOM 530 0C53 GRA 1 61.879 35.578 3.481

ATOM 531 1C53 GRA 1 61.879 40.498 3.481

ATOM 532 2C53 GRA 1 66.140 28.198 3.481

ATOM 533 3C53 GRA 1 66.140 33.118 3.481

ATOM 534 4C53 GRA 1 0.807 47.878 3.481

ATOM 535 5C53 GRA 1 5.068 40.498 3.481

ATOM 536 6C53 GRA 1 5.068 45.418 3.481

ATOM 537 7C53 GRA 1 5.068 50.338 3.481

ATOM 538 8C53 GRA 1 9.329 33.118 3.481

ATOM 539 9C53 GRA 1 9.329 38.038 3.481

ATOM 540 0C54 GRA 1 9.329 42.958 3.481

ATOM 541 1C54 GRA 1 9.329 47.878 3.481

ATOM 542 2C54 GRA 1 9.329 52.798 3.481

ATOM 543 3C54 GRA 1 13.589 25.738 3.481

ATOM 544 4C54 GRA 1 13.589 30.658 3.481

ATOM 545 5C54 GRA 1 13.589 35.578 3.481

ATOM 546 6C54 GRA 1 13.589 40.498 3.481

ATOM 547 7C54 GRA 1 13.589 45.418 3.481

ATOM 548 8C54 GRA 1 13.589 50.338 3.481

ATOM 549 9C54 GRA 1 13.589 55.258 3.481

ATOM 550 0C55 GRA 1 17.850 18.358 3.481

ATOM 551 1C55 GRA 1 17.850 23.278 3.481

ATOM 552 2C55 GRA 1 17.850 28.198 3.481

ATOM 553 3C55 GRA 1 17.850 33.118 3.481

ATOM 554 4C55 GRA 1 17.850 38.038 3.481

ATOM 555 5C55 GRA 1 17.850 42.958 3.481

ATOM 556 6C55 GRA 1 17.850 47.878 3.481

ATOM 557 7C55 GRA 1 17.850 52.798 3.481

ATOM 558 8C55 GRA 1 17.850 57.718 3.481

ATOM 559 9C55 GRA 1 22.111 10.978 3.481

ATOM 560 0C56 GRA 1 22.111 15.898 3.481

ATOM 561 1C56 GRA 1 22.111 20.818 3.481

ATOM 562 2C56 GRA 1 22.111 25.738 3.481

ATOM 563 3C56 GRA 1 22.111 30.658 3.481

ATOM 564 4C56 GRA 1 22.111 35.578 3.481

ATOM 565 5C56 GRA 1 22.111 40.498 3.481

ATOM 566 6C56 GRA 1 22.111 45.418 3.481

ATOM 567 7C56 GRA 1 22.111 50.338 3.481

ATOM 568 8C56 GRA 1 22.111 55.258 3.481

ATOM 569 9C56 GRA 1 22.111 60.178 3.481

ATOM 570 0C57 GRA 1 26.372 3.598 3.481

ATOM 571 1C57 GRA 1 26.372 8.518 3.481

ATOM 572 2C57 GRA 1 26.372 13.438 3.481

ATOM 573 3C57 GRA 1 26.372 18.358 3.481

ATOM 574 4C57 GRA 1 26.372 23.278 3.481

ATOM 575 5C57 GRA 1 26.372 28.198 3.481

ATOM 576 6C57 GRA 1 26.372 33.118 3.481

ATOM 577 7C57 GRA 1 26.372 38.038 3.481

ATOM 578 8C57 GRA 1 26.372 42.958 3.481

ATOM 579 9C57 GRA 1 26.372 47.878 3.481

ATOM 580 0C58 GRA 1 26.372 52.798 3.481

ATOM 581 1C58 GRA 1 26.372 57.718 3.481

ATOM 582 2C58 GRA 1 26.372 62.638 3.481

ATOM 583 3C58 GRA 1 30.633 6.058 3.481

ATOM 584 4C58 GRA 1 30.633 10.978 3.481

ATOM 585 5C58 GRA 1 30.633 15.898 3.481

ATOM 586 6C58 GRA 1 30.633 20.818 3.481

ATOM 587 7C58 GRA 1 30.633 25.738 3.481

ATOM 588 8C58 GRA 1 30.633 30.658 3.481

ATOM 589 9C58 GRA 1 30.633 35.578 3.481

ATOM 590 0C59 GRA 1 30.633 40.498 3.481

ATOM 591 1C59 GRA 1 30.633 45.418 3.481

ATOM 592 2C59 GRA 1 30.633 50.338 3.481

ATOM 593 3C59 GRA 1 30.633 55.258 3.481

ATOM 594 4C59 GRA 1 30.633 60.178 3.481

ATOM 595 5C59 GRA 1 30.633 65.098 3.481

ATOM 596 6C59 GRA 1 34.894 8.518 3.481

ATOM 597 7C59 GRA 1 34.894 13.438 3.481

ATOM 598 8C59 GRA 1 34.894 18.358 3.481

ATOM 599 9C59 GRA 1 34.894 23.278 3.481

ATOM 600 0C60 GRA 1 34.894 28.198 3.481

ATOM 601 1C60 GRA 1 34.894 33.118 3.481

ATOM 602 2C60 GRA 1 34.894 38.038 3.481

ATOM 603 3C60 GRA 1 34.894 42.958 3.481

ATOM 604 4C60 GRA 1 34.894 47.878 3.481

ATOM 605 5C60 GRA 1 34.894 52.798 3.481

ATOM 606 6C60 GRA 1 34.894 57.718 3.481

ATOM 607 7C60 GRA 1 34.894 62.638 3.481

ATOM 608 8C60 GRA 1 34.894 67.558 3.481

ATOM 609 9C60 GRA 1 39.154 10.978 3.481

ATOM 610 0C61 GRA 1 39.154 15.898 3.481

ATOM 611 1C61 GRA 1 39.154 20.818 3.481

ATOM 612 2C61 GRA 1 39.154 25.738 3.481

ATOM 613 3C61 GRA 1 39.154 30.658 3.481

ATOM 614 4C61 GRA 1 39.154 35.578 3.481

ATOM 615 5C61 GRA 1 39.154 40.498 3.481

ATOM 616 6C61 GRA 1 39.154 45.418 3.481

ATOM 617 7C61 GRA 1 39.154 50.338 3.481

ATOM 618 8C61 GRA 1 39.154 55.258 3.481

ATOM 619 9C61 GRA 1 39.154 60.178 3.481

ATOM 620 0C62 GRA 1 39.154 65.098 3.481

ATOM 621 1C62 GRA 1 39.154 70.018 3.481

ATOM 622 2C62 GRA 1 43.415 13.438 3.481

ATOM 623 3C62 GRA 1 43.415 18.358 3.481

ATOM 624 4C62 GRA 1 43.415 23.278 3.481

ATOM 625 5C62 GRA 1 43.415 28.198 3.481

ATOM 626 6C62 GRA 1 43.415 33.118 3.481

ATOM 627 7C62 GRA 1 43.415 38.038 3.481

ATOM 628 8C62 GRA 1 43.415 42.958 3.481

ATOM 629 9C62 GRA 1 43.415 47.878 3.481

ATOM 630 0C63 GRA 1 43.415 52.798 3.481

ATOM 631 1C63 GRA 1 43.415 57.718 3.481

ATOM 632 2C63 GRA 1 43.415 62.638 3.481

ATOM 633 3C63 GRA 1 43.415 67.558 3.481

ATOM 634 4C63 GRA 1 43.415 72.478 3.481

ATOM 635 5C63 GRA 1 47.676 15.898 3.481

ATOM 636 6C63 GRA 1 47.676 20.818 3.481

ATOM 637 7C63 GRA 1 47.676 25.738 3.481

ATOM 638 8C63 GRA 1 47.676 30.658 3.481

ATOM 639 9C63 GRA 1 47.676 35.578 3.481

ATOM 640 0C64 GRA 1 47.676 40.498 3.481

ATOM 641 1C64 GRA 1 47.676 45.418 3.481

ATOM 642 2C64 GRA 1 47.676 50.338 3.481

ATOM 643 3C64 GRA 1 47.676 55.258 3.481

ATOM 644 4C64 GRA 1 47.676 60.178 3.481

ATOM 645 5C64 GRA 1 47.676 65.098 3.481

ATOM 646 6C64 GRA 1 51.937 18.358 3.481

ATOM 647 7C64 GRA 1 51.937 23.278 3.481

ATOM 648 8C64 GRA 1 51.937 28.198 3.481

ATOM 649 9C64 GRA 1 51.937 33.118 3.481

ATOM 650 0C65 GRA 1 51.937 38.038 3.481

ATOM 651 1C65 GRA 1 51.937 42.958 3.481

ATOM 652 2C65 GRA 1 51.937 47.878 3.481

ATOM 653 3C65 GRA 1 51.937 52.798 3.481

ATOM 654 4C65 GRA 1 51.937 57.718 3.481

ATOM 655 5C65 GRA 1 56.198 20.818 3.481

ATOM 656 6C65 GRA 1 56.198 25.738 3.481

ATOM 657 7C65 GRA 1 56.198 30.658 3.481

ATOM 658 8C65 GRA 1 56.198 35.578 3.481

ATOM 659 9C65 GRA 1 56.198 40.498 3.481

ATOM 660 0C66 GRA 1 56.198 45.418 3.481

ATOM 661 1C66 GRA 1 56.198 50.338 3.481

ATOM 662 2C66 GRA 1 60.459 23.278 3.481

ATOM 663 3C66 GRA 1 60.459 28.198 3.481

ATOM 664 4C66 GRA 1 60.459 33.118 3.481

ATOM 665 5C66 GRA 1 60.459 38.038 3.481

ATOM 666 6C66 GRA 1 60.459 42.958 3.481

ATOM 667 7C66 GRA 1 64.720 25.738 3.481

ATOM 668 8C66 GRA 1 64.720 30.658 3.481

ATOM 669 9C66 GRA 1 64.720 35.578 3.481

ATOM 670 0C67 GRA 1 68.980 28.198 3.481

ATOM 671 1C67 GRA 1 0.807 50.338 3.481

ATOM 672 2C67 GRA 1 5.068 42.958 3.481

ATOM 673 3C67 GRA 1 5.068 47.878 3.481

ATOM 674 4C67 GRA 1 5.068 52.798 3.481

ATOM 675 5C67 GRA 1 9.329 35.578 3.481

ATOM 676 6C67 GRA 1 9.329 40.498 3.481

ATOM 677 7C67 GRA 1 9.329 45.418 3.481

ATOM 678 8C67 GRA 1 9.329 50.338 3.481

ATOM 679 9C67 GRA 1 9.329 55.258 3.481

ATOM 680 0C68 GRA 1 13.589 28.198 3.481

ATOM 681 1C68 GRA 1 13.589 33.118 3.481

ATOM 682 2C68 GRA 1 13.589 38.038 3.481

ATOM 683 3C68 GRA 1 13.589 42.958 3.481

ATOM 684 4C68 GRA 1 13.589 47.878 3.481

ATOM 685 5C68 GRA 1 13.589 52.798 3.481

ATOM 686 6C68 GRA 1 13.589 57.718 3.481

ATOM 687 7C68 GRA 1 17.850 20.818 3.481

ATOM 688 8C68 GRA 1 17.850 25.738 3.481

ATOM 689 9C68 GRA 1 17.850 30.658 3.481

ATOM 690 0C69 GRA 1 17.850 35.578 3.481

ATOM 691 1C69 GRA 1 17.850 40.498 3.481

ATOM 692 2C69 GRA 1 17.850 45.418 3.481

ATOM 693 3C69 GRA 1 17.850 50.338 3.481

ATOM 694 4C69 GRA 1 17.850 55.258 3.481

ATOM 695 5C69 GRA 1 17.850 60.178 3.481

ATOM 696 6C69 GRA 1 22.111 13.438 3.481

ATOM 697 7C69 GRA 1 22.111 18.358 3.481

ATOM 698 8C69 GRA 1 22.111 23.278 3.481

ATOM 699 9C69 GRA 1 22.111 28.198 3.481

ATOM 700 0C70 GRA 1 22.111 33.118 3.481

ATOM 701 1C70 GRA 1 22.111 38.038 3.481

ATOM 702 2C70 GRA 1 22.111 42.958 3.481

ATOM 703 3C70 GRA 1 22.111 47.878 3.481

ATOM 704 4C70 GRA 1 22.111 52.798 3.481

ATOM 705 5C70 GRA 1 22.111 57.718 3.481

ATOM 706 6C70 GRA 1 22.111 62.638 3.481

ATOM 707 7C70 GRA 1 26.372 6.058 3.481

ATOM 708 8C70 GRA 1 26.372 10.978 3.481

ATOM 709 9C70 GRA 1 26.372 15.898 3.481

ATOM 710 0C71 GRA 1 26.372 20.818 3.481

ATOM 711 1C71 GRA 1 26.372 25.738 3.481

ATOM 712 2C71 GRA 1 26.372 30.658 3.481

ATOM 713 3C71 GRA 1 26.372 35.578 3.481

ATOM 714 4C71 GRA 1 26.372 40.498 3.481

ATOM 715 5C71 GRA 1 26.372 45.418 3.481

ATOM 716 6C71 GRA 1 26.372 50.338 3.481

ATOM 717 7C71 GRA 1 26.372 55.258 3.481

ATOM 718 8C71 GRA 1 26.372 60.178 3.481

ATOM 719 9C71 GRA 1 26.372 65.098 3.481

ATOM 720 0C72 GRA 1 30.633 8.518 3.481

ATOM 721 1C72 GRA 1 30.633 13.438 3.481

ATOM 722 2C72 GRA 1 30.633 18.358 3.481

ATOM 723 3C72 GRA 1 30.633 23.278 3.481

ATOM 724 4C72 GRA 1 30.633 28.198 3.481

ATOM 725 5C72 GRA 1 30.633 33.118 3.481

ATOM 726 6C72 GRA 1 30.633 38.038 3.481

ATOM 727 7C72 GRA 1 30.633 42.958 3.481

ATOM 728 8C72 GRA 1 30.633 47.878 3.481

ATOM 729 9C72 GRA 1 30.633 52.798 3.481

ATOM 730 0C73 GRA 1 30.633 57.718 3.481

ATOM 731 1C73 GRA 1 30.633 62.638 3.481

ATOM 732 2C73 GRA 1 30.633 67.558 3.481

ATOM 733 3C73 GRA 1 34.894 10.978 3.481

ATOM 734 4C73 GRA 1 34.894 15.898 3.481

ATOM 735 5C73 GRA 1 34.894 20.818 3.481

ATOM 736 6C73 GRA 1 34.894 25.738 3.481

ATOM 737 7C73 GRA 1 34.894 30.658 3.481

ATOM 738 8C73 GRA 1 34.894 35.578 3.481

ATOM 739 9C73 GRA 1 34.894 40.498 3.481

ATOM 740 0C74 GRA 1 34.894 45.418 3.481

ATOM 741 1C74 GRA 1 34.894 50.338 3.481

ATOM 742 2C74 GRA 1 34.894 55.258 3.481

ATOM 743 3C74 GRA 1 34.894 60.178 3.481

ATOM 744 4C74 GRA 1 34.894 65.098 3.481

ATOM 745 5C74 GRA 1 34.894 70.018 3.481

ATOM 746 6C74 GRA 1 39.154 13.438 3.481

ATOM 747 7C74 GRA 1 39.154 18.358 3.481

ATOM 748 8C74 GRA 1 39.154 23.278 3.481

ATOM 749 9C74 GRA 1 39.154 28.198 3.481

ATOM 750 0C75 GRA 1 39.154 33.118 3.481

ATOM 751 1C75 GRA 1 39.154 38.038 3.481

ATOM 752 2C75 GRA 1 39.154 42.958 3.481

ATOM 753 3C75 GRA 1 39.154 47.878 3.481

ATOM 754 4C75 GRA 1 39.154 52.798 3.481

ATOM 755 5C75 GRA 1 39.154 57.718 3.481

ATOM 756 6C75 GRA 1 39.154 62.638 3.481

ATOM 757 7C75 GRA 1 39.154 67.558 3.481

ATOM 758 8C75 GRA 1 39.154 72.478 3.481

ATOM 759 9C75 GRA 1 43.415 15.898 3.481

ATOM 760 0C76 GRA 1 43.415 20.818 3.481

ATOM 761 1C76 GRA 1 43.415 25.738 3.481

ATOM 762 2C76 GRA 1 43.415 30.658 3.481

ATOM 763 3C76 GRA 1 43.415 35.578 3.481

ATOM 764 4C76 GRA 1 43.415 40.498 3.481

ATOM 765 5C76 GRA 1 43.415 45.418 3.481

ATOM 766 6C76 GRA 1 43.415 50.338 3.481

ATOM 767 7C76 GRA 1 43.415 55.258 3.481

ATOM 768 8C76 GRA 1 43.415 60.178 3.481

ATOM 769 9C76 GRA 1 43.415 65.098 3.481

ATOM 770 0C77 GRA 1 43.415 70.018 3.481

ATOM 771 1C77 GRA 1 47.676 18.358 3.481

ATOM 772 2C77 GRA 1 47.676 23.278 3.481

ATOM 773 3C77 GRA 1 47.676 28.198 3.481

ATOM 774 4C77 GRA 1 47.676 33.118 3.481

ATOM 775 5C77 GRA 1 47.676 38.038 3.481

ATOM 776 6C77 GRA 1 47.676 42.958 3.481

ATOM 777 7C77 GRA 1 47.676 47.878 3.481

ATOM 778 8C77 GRA 1 47.676 52.798 3.481

ATOM 779 9C77 GRA 1 47.676 57.718 3.481

ATOM 780 0C78 GRA 1 47.676 62.638 3.481

ATOM 781 1C78 GRA 1 51.937 20.818 3.481

ATOM 782 2C78 GRA 1 51.937 25.738 3.481

ATOM 783 3C78 GRA 1 51.937 30.658 3.481

ATOM 784 4C78 GRA 1 51.937 35.578 3.481

ATOM 785 5C78 GRA 1 51.937 40.498 3.481

ATOM 786 6C78 GRA 1 51.937 45.418 3.481

ATOM 787 7C78 GRA 1 51.937 50.338 3.481

ATOM 788 8C78 GRA 1 51.937 55.258 3.481

ATOM 789 9C78 GRA 1 56.198 23.278 3.481

ATOM 790 0C79 GRA 1 56.198 28.198 3.481

ATOM 791 1C79 GRA 1 56.198 33.118 3.481

ATOM 792 2C79 GRA 1 56.198 38.038 3.481

ATOM 793 3C79 GRA 1 56.198 42.958 3.481

ATOM 794 4C79 GRA 1 56.198 47.878 3.481

ATOM 795 5C79 GRA 1 60.459 25.738 3.481

ATOM 796 6C79 GRA 1 60.459 30.658 3.481

ATOM 797 7C79 GRA 1 60.459 35.578 3.481

ATOM 798 8C79 GRA 1 60.459 40.498 3.481

ATOM 799 9C79 GRA 1 64.720 28.198 3.481

ATOM 800 0C80 GRA 1 64.720 33.118 3.481

ATOM 801 1C80 GRA 1 2.937 46.648 3.481

ATOM 802 2C80 GRA 1 2.937 51.568 3.481

ATOM 803 3C80 GRA 1 7.198 39.268 3.481

ATOM 804 4C80 GRA 1 7.198 44.188 3.481

ATOM 805 5C80 GRA 1 7.198 49.108 3.481

ATOM 806 6C80 GRA 1 7.198 54.028 3.481

ATOM 807 7C80 GRA 1 11.459 31.888 3.481

ATOM 808 8C80 GRA 1 11.459 36.808 3.481

ATOM 809 9C80 GRA 1 11.459 41.728 3.481

ATOM 810 0C81 GRA 1 11.459 46.648 3.481

ATOM 811 1C81 GRA 1 11.459 51.568 3.481

ATOM 812 2C81 GRA 1 11.459 56.488 3.481

ATOM 813 3C81 GRA 1 15.720 24.508 3.481

ATOM 814 4C81 GRA 1 15.720 29.428 3.481

ATOM 815 5C81 GRA 1 15.720 34.348 3.481

ATOM 816 6C81 GRA 1 15.720 39.268 3.481

ATOM 817 7C81 GRA 1 15.720 44.188 3.481

ATOM 818 8C81 GRA 1 15.720 49.108 3.481

ATOM 819 9C81 GRA 1 15.720 54.028 3.481

ATOM 820 0C82 GRA 1 15.720 58.948 3.481

ATOM 821 1C82 GRA 1 19.981 17.128 3.481

ATOM 822 2C82 GRA 1 19.981 22.048 3.481

ATOM 823 3C82 GRA 1 19.981 26.968 3.481

ATOM 824 4C82 GRA 1 19.981 31.888 3.481

ATOM 825 5C82 GRA 1 19.981 36.808 3.481

ATOM 826 6C82 GRA 1 19.981 41.728 3.481

ATOM 827 7C82 GRA 1 19.981 46.648 3.481

ATOM 828 8C82 GRA 1 19.981 51.568 3.481

ATOM 829 9C82 GRA 1 19.981 56.488 3.481

ATOM 830 0C83 GRA 1 19.981 61.408 3.481

ATOM 831 1C83 GRA 1 24.242 9.748 3.481

ATOM 832 2C83 GRA 1 24.242 14.668 3.481

ATOM 833 3C83 GRA 1 24.242 19.588 3.481

ATOM 834 4C83 GRA 1 24.242 24.508 3.481

ATOM 835 5C83 GRA 1 24.242 29.428 3.481

ATOM 836 6C83 GRA 1 24.242 34.348 3.481

ATOM 837 7C83 GRA 1 24.242 39.268 3.481

ATOM 838 8C83 GRA 1 24.242 44.188 3.481

ATOM 839 9C83 GRA 1 24.242 49.108 3.481

ATOM 840 0C84 GRA 1 24.242 54.028 3.481

ATOM 841 1C84 GRA 1 24.242 58.948 3.481

ATOM 842 2C84 GRA 1 24.242 63.868 3.481

ATOM 843 3C84 GRA 1 28.502 7.288 3.481

ATOM 844 4C84 GRA 1 28.502 12.208 3.481

ATOM 845 5C84 GRA 1 28.502 17.128 3.481

ATOM 846 6C84 GRA 1 28.502 22.048 3.481

ATOM 847 7C84 GRA 1 28.502 26.968 3.481

ATOM 848 8C84 GRA 1 28.502 31.888 3.481

ATOM 849 9C84 GRA 1 28.502 36.808 3.481

ATOM 850 0C85 GRA 1 28.502 41.728 3.481

ATOM 851 1C85 GRA 1 28.502 46.648 3.481

ATOM 852 2C85 GRA 1 28.502 51.568 3.481

ATOM 853 3C85 GRA 1 28.502 56.488 3.481

ATOM 854 4C85 GRA 1 28.502 61.408 3.481

ATOM 855 5C85 GRA 1 28.502 66.328 3.481

ATOM 856 6C85 GRA 1 32.763 9.748 3.481

ATOM 857 7C85 GRA 1 32.763 14.668 3.481

ATOM 858 8C85 GRA 1 32.763 19.588 3.481

ATOM 859 9C85 GRA 1 32.763 24.508 3.481

ATOM 860 0C86 GRA 1 32.763 29.428 3.481

ATOM 861 1C86 GRA 1 32.763 34.348 3.481

ATOM 862 2C86 GRA 1 32.763 39.268 3.481

ATOM 863 3C86 GRA 1 32.763 44.188 3.481

ATOM 864 4C86 GRA 1 32.763 49.108 3.481

ATOM 865 5C86 GRA 1 32.763 54.028 3.481

ATOM 866 6C86 GRA 1 32.763 58.948 3.481

ATOM 867 7C86 GRA 1 32.763 63.868 3.481

ATOM 868 8C86 GRA 1 32.763 68.788 3.481

ATOM 869 9C86 GRA 1 37.024 12.208 3.481

ATOM 870 0C87 GRA 1 37.024 17.128 3.481

ATOM 871 1C87 GRA 1 37.024 22.048 3.481

ATOM 872 2C87 GRA 1 37.024 26.968 3.481

ATOM 873 3C87 GRA 1 37.024 31.888 3.481

ATOM 874 4C87 GRA 1 37.024 36.808 3.481

ATOM 875 5C87 GRA 1 37.024 41.728 3.481

ATOM 876 6C87 GRA 1 37.024 46.648 3.481

ATOM 877 7C87 GRA 1 37.024 51.568 3.481

ATOM 878 8C87 GRA 1 37.024 56.488 3.481

ATOM 879 9C87 GRA 1 37.024 61.408 3.481

ATOM 880 0C88 GRA 1 37.024 66.328 3.481

ATOM 881 1C88 GRA 1 37.024 71.248 3.481

ATOM 882 2C88 GRA 1 41.285 14.668 3.481

ATOM 883 3C88 GRA 1 41.285 19.588 3.481

ATOM 884 4C88 GRA 1 41.285 24.508 3.481

ATOM 885 5C88 GRA 1 41.285 29.428 3.481

ATOM 886 6C88 GRA 1 41.285 34.348 3.481

ATOM 887 7C88 GRA 1 41.285 39.268 3.481

ATOM 888 8C88 GRA 1 41.285 44.188 3.481

ATOM 889 9C88 GRA 1 41.285 49.108 3.481

ATOM 890 0C89 GRA 1 41.285 54.028 3.481

ATOM 891 1C89 GRA 1 41.285 58.948 3.481

ATOM 892 2C89 GRA 1 41.285 63.868 3.481

ATOM 893 3C89 GRA 1 41.285 68.788 3.481

ATOM 894 4C89 GRA 1 41.285 73.708 3.481

ATOM 895 5C89 GRA 1 45.546 17.128 3.481

ATOM 896 6C89 GRA 1 45.546 22.048 3.481

ATOM 897 7C89 GRA 1 45.546 26.968 3.481

ATOM 898 8C89 GRA 1 45.546 31.888 3.481

ATOM 899 9C89 GRA 1 45.546 36.808 3.481

ATOM 900 0C90 GRA 1 45.546 41.728 3.481

ATOM 901 1C90 GRA 1 45.546 46.648 3.481

ATOM 902 2C90 GRA 1 45.546 51.568 3.481

ATOM 903 3C90 GRA 1 45.546 56.488 3.481

ATOM 904 4C90 GRA 1 45.546 61.408 3.481

ATOM 905 5C90 GRA 1 45.546 66.328 3.481

ATOM 906 6C90 GRA 1 49.807 19.588 3.481

ATOM 907 7C90 GRA 1 49.807 24.508 3.481

ATOM 908 8C90 GRA 1 49.807 29.428 3.481

ATOM 909 9C90 GRA 1 49.807 34.348 3.481

ATOM 910 0C91 GRA 1 49.807 39.268 3.481

ATOM 911 1C91 GRA 1 49.807 44.188 3.481

ATOM 912 2C91 GRA 1 49.807 49.108 3.481

ATOM 913 3C91 GRA 1 49.807 54.028 3.481

ATOM 914 4C91 GRA 1 49.807 58.948 3.481

ATOM 915 5C91 GRA 1 54.067 22.048 3.481

ATOM 916 6C91 GRA 1 54.067 26.968 3.481

ATOM 917 7C91 GRA 1 54.067 31.888 3.481

ATOM 918 8C91 GRA 1 54.067 36.808 3.481

ATOM 919 9C91 GRA 1 54.067 41.728 3.481

ATOM 920 0C92 GRA 1 54.067 46.648 3.481

ATOM 921 1C92 GRA 1 54.067 51.568 3.481

ATOM 922 2C92 GRA 1 58.328 24.508 3.481

ATOM 923 3C92 GRA 1 58.328 29.428 3.481

ATOM 924 4C92 GRA 1 58.328 34.348 3.481

ATOM 925 5C92 GRA 1 58.328 39.268 3.481

ATOM 926 6C92 GRA 1 58.328 44.188 3.481

ATOM 927 7C92 GRA 1 62.589 26.968 3.481

ATOM 928 8C92 GRA 1 62.589 31.888 3.481

ATOM 929 9C92 GRA 1 62.589 36.808 3.481

ATOM 930 0C93 GRA 1 66.850 29.428 3.481

ATOM 931 1C93 GRA 1 2.937 44.188 3.481

ATOM 932 2C93 GRA 1 2.937 49.108 3.481

ATOM 933 3C93 GRA 1 7.198 36.808 3.481

ATOM 934 4C93 GRA 1 7.198 41.728 3.481

ATOM 935 5C93 GRA 1 7.198 46.648 3.481

ATOM 936 6C93 GRA 1 7.198 51.568 3.481

ATOM 937 7C93 GRA 1 11.459 29.428 3.481

ATOM 938 8C93 GRA 1 11.459 34.348 3.481

ATOM 939 9C93 GRA 1 11.459 39.268 3.481

ATOM 940 0C94 GRA 1 11.459 44.188 3.481

ATOM 941 1C94 GRA 1 11.459 49.108 3.481

ATOM 942 2C94 GRA 1 11.459 54.028 3.481

ATOM 943 3C94 GRA 1 15.720 22.048 3.481

ATOM 944 4C94 GRA 1 15.720 26.968 3.481

ATOM 945 5C94 GRA 1 15.720 31.888 3.481

ATOM 946 6C94 GRA 1 15.720 36.808 3.481

ATOM 947 7C94 GRA 1 15.720 41.728 3.481

ATOM 948 8C94 GRA 1 15.720 46.648 3.481

ATOM 949 9C94 GRA 1 15.720 51.568 3.481

ATOM 950 0C95 GRA 1 15.720 56.488 3.481

ATOM 951 1C95 GRA 1 19.981 14.668 3.481

ATOM 952 2C95 GRA 1 19.981 19.588 3.481

ATOM 953 3C95 GRA 1 19.981 24.508 3.481

ATOM 954 4C95 GRA 1 19.981 29.428 3.481

ATOM 955 5C95 GRA 1 19.981 34.348 3.481

ATOM 956 6C95 GRA 1 19.981 39.268 3.481

ATOM 957 7C95 GRA 1 19.981 44.188 3.481

ATOM 958 8C95 GRA 1 19.981 49.108 3.481

ATOM 959 9C95 GRA 1 19.981 54.028 3.481

ATOM 960 0C96 GRA 1 19.981 58.948 3.481

ATOM 961 1C96 GRA 1 24.242 7.288 3.481

ATOM 962 2C96 GRA 1 24.242 12.208 3.481

ATOM 963 3C96 GRA 1 24.242 17.128 3.481

ATOM 964 4C96 GRA 1 24.242 22.048 3.481

ATOM 965 5C96 GRA 1 24.242 26.968 3.481

ATOM 966 6C96 GRA 1 24.242 31.888 3.481

ATOM 967 7C96 GRA 1 24.242 36.808 3.481

ATOM 968 8C96 GRA 1 24.242 41.728 3.481

ATOM 969 9C96 GRA 1 24.242 46.648 3.481

ATOM 970 0C97 GRA 1 24.242 51.568 3.481

ATOM 971 1C97 GRA 1 24.242 56.488 3.481

ATOM 972 2C97 GRA 1 24.242 61.408 3.481

ATOM 973 3C97 GRA 1 28.502 4.828 3.481

ATOM 974 4C97 GRA 1 28.502 9.748 3.481

ATOM 975 5C97 GRA 1 28.502 14.668 3.481

ATOM 976 6C97 GRA 1 28.502 19.588 3.481

ATOM 977 7C97 GRA 1 28.502 24.508 3.481

ATOM 978 8C97 GRA 1 28.502 29.428 3.481

ATOM 979 9C97 GRA 1 28.502 34.348 3.481

ATOM 980 0C98 GRA 1 28.502 39.268 3.481

ATOM 981 1C98 GRA 1 28.502 44.188 3.481

ATOM 982 2C98 GRA 1 28.502 49.108 3.481

ATOM 983 3C98 GRA 1 28.502 54.028 3.481

ATOM 984 4C98 GRA 1 28.502 58.948 3.481

ATOM 985 5C98 GRA 1 28.502 63.868 3.481

ATOM 986 6C98 GRA 1 32.763 7.288 3.481

ATOM 987 7C98 GRA 1 32.763 12.208 3.481

ATOM 988 8C98 GRA 1 32.763 17.128 3.481

ATOM 989 9C98 GRA 1 32.763 22.048 3.481

ATOM 990 0C99 GRA 1 32.763 26.968 3.481

ATOM 991 1C99 GRA 1 32.763 31.888 3.481

ATOM 992 2C99 GRA 1 32.763 36.808 3.481

ATOM 993 3C99 GRA 1 32.763 41.728 3.481

ATOM 994 4C99 GRA 1 32.763 46.648 3.481

ATOM 995 5C99 GRA 1 32.763 51.568 3.481

ATOM 996 6C99 GRA 1 32.763 56.488 3.481

ATOM 997 7C99 GRA 1 32.763 61.408 3.481

ATOM 998 8C99 GRA 1 32.763 66.328 3.481

ATOM 999 9C99 GRA 1 37.024 9.748 3.481

ATOM 1000 0C00 GRA 1 37.024 14.668 3.481

ATOM 1001 1C00 GRA 1 37.024 19.588 3.481

ATOM 1002 2C00 GRA 1 37.024 24.508 3.481

ATOM 1003 3C00 GRA 1 37.024 29.428 3.481

ATOM 1004 4C00 GRA 1 37.024 34.348 3.481

ATOM 1005 5C00 GRA 1 37.024 39.268 3.481

ATOM 1006 6C00 GRA 1 37.024 44.188 3.481

ATOM 1007 7C00 GRA 1 37.024 49.108 3.481

ATOM 1008 8C00 GRA 1 37.024 54.028 3.481

ATOM 1009 9C00 GRA 1 37.024 58.948 3.481

ATOM 1010 0C01 GRA 1 37.024 63.868 3.481

ATOM 1011 1C01 GRA 1 37.024 68.788 3.481

ATOM 1012 2C01 GRA 1 41.285 12.208 3.481

ATOM 1013 3C01 GRA 1 41.285 17.128 3.481

ATOM 1014 4C01 GRA 1 41.285 22.048 3.481

ATOM 1015 5C01 GRA 1 41.285 26.968 3.481

ATOM 1016 6C01 GRA 1 41.285 31.888 3.481

ATOM 1017 7C01 GRA 1 41.285 36.808 3.481

ATOM 1018 8C01 GRA 1 41.285 41.728 3.481

ATOM 1019 9C01 GRA 1 41.285 46.648 3.481

ATOM 1020 0C02 GRA 1 41.285 51.568 3.481

ATOM 1021 1C02 GRA 1 41.285 56.488 3.481

ATOM 1022 2C02 GRA 1 41.285 61.408 3.481

ATOM 1023 3C02 GRA 1 41.285 66.328 3.481

ATOM 1024 4C02 GRA 1 41.285 71.248 3.481

ATOM 1025 5C02 GRA 1 45.546 14.668 3.481

ATOM 1026 6C02 GRA 1 45.546 19.588 3.481

ATOM 1027 7C02 GRA 1 45.546 24.508 3.481

ATOM 1028 8C02 GRA 1 45.546 29.428 3.481

ATOM 1029 9C02 GRA 1 45.546 34.348 3.481

ATOM 1030 0C03 GRA 1 45.546 39.268 3.481

ATOM 1031 1C03 GRA 1 45.546 44.188 3.481

ATOM 1032 2C03 GRA 1 45.546 49.108 3.481

ATOM 1033 3C03 GRA 1 45.546 54.028 3.481

ATOM 1034 4C03 GRA 1 45.546 58.948 3.481

ATOM 1035 5C03 GRA 1 45.546 63.868 3.481

ATOM 1036 6C03 GRA 1 45.546 68.788 3.481

ATOM 1037 7C03 GRA 1 49.807 17.128 3.481

ATOM 1038 8C03 GRA 1 49.807 22.048 3.481

ATOM 1039 9C03 GRA 1 49.807 26.968 3.481

ATOM 1040 0C04 GRA 1 49.807 31.888 3.481

ATOM 1041 1C04 GRA 1 49.807 36.808 3.481

ATOM 1042 2C04 GRA 1 49.807 41.728 3.481

ATOM 1043 3C04 GRA 1 49.807 46.648 3.481

ATOM 1044 4C04 GRA 1 49.807 51.568 3.481

ATOM 1045 5C04 GRA 1 49.807 56.488 3.481

ATOM 1046 6C04 GRA 1 49.807 61.408 3.481

ATOM 1047 7C04 GRA 1 54.067 19.588 3.481

ATOM 1048 8C04 GRA 1 54.067 24.508 3.481

ATOM 1049 9C04 GRA 1 54.067 29.428 3.481

ATOM 1050 0C05 GRA 1 54.067 34.348 3.481

ATOM 1051 1C05 GRA 1 54.067 39.268 3.481

ATOM 1052 2C05 GRA 1 54.067 44.188 3.481

ATOM 1053 3C05 GRA 1 54.067 49.108 3.481

ATOM 1054 4C05 GRA 1 54.067 54.028 3.481

ATOM 1055 5C05 GRA 1 58.328 22.048 3.481

ATOM 1056 6C05 GRA 1 58.328 26.968 3.481

ATOM 1057 7C05 GRA 1 58.328 31.888 3.481

ATOM 1058 8C05 GRA 1 58.328 36.808 3.481

ATOM 1059 9C05 GRA 1 58.328 41.728 3.481

ATOM 1060 0C06 GRA 1 58.328 46.648 3.481

ATOM 1061 1C06 GRA 1 62.589 24.508 3.481

ATOM 1062 2C06 GRA 1 62.589 29.428 3.481

ATOM 1063 3C06 GRA 1 62.589 34.348 3.481

ATOM 1064 4C06 GRA 1 62.589 39.268 3.481

ATOM 1065 5C06 GRA 1 66.850 26.968 3.481

ATOM 1066 6C06 GRA 1 66.850 31.888 3.481

TER

END
