# Supplementary material for: Exploring the Influence of Carbon Nanoparticles on the Formation of β-Sheet-Rich Oligomers of IAPP22–28 Peptide by Molecular Dynamics Simulation
Source: PLoS One. 2013 Jun 5;8(6):e65579. doi: 10.1371/journal.pone.0065579 (PMC3674003; doi:10.1371/journal.pone.0065579)
Supplement: Text S3 — Coordinates of graphene interacting with 8 peptides. (DOC) [file pone.0065579.s005.doc]

**Text S3. Coordinates of graphene interacting with 8 peptides.**

ATOM 1 C1 GRA 1 10.318 68.947 10.391

ATOM 2 C2 GRA 1 10.634 60.474 9.538

ATOM 3 C3 GRA 1 12.933 64.792 10.063

ATOM 4 C4 GRA 1 15.232 69.110 10.587

ATOM 5 C5 GRA 1 10.949 52.001 8.685

ATOM 6 C6 GRA 1 13.247 56.319 9.210

ATOM 7 C7 GRA 1 15.546 60.637 9.735

ATOM 8 C8 GRA 1 17.845 64.956 10.259

ATOM 9 C9 GRA 1 20.144 69.274 10.784

ATOM 10 C10 GRA 1 11.264 43.528 7.833

ATOM 11 C11 GRA 1 13.563 47.846 8.357

ATOM 12 C12 GRA 1 15.862 52.164 8.882

ATOM 13 C13 GRA 1 18.161 56.482 9.406

ATOM 14 C14 GRA 1 20.460 60.801 9.931

ATOM 15 C15 GRA 1 22.759 65.119 10.455

ATOM 16 C16 GRA 1 25.058 69.437 10.980

ATOM 17 C17 GRA 1 11.580 35.055 6.980

ATOM 18 C18 GRA 1 13.879 39.373 7.505

ATOM 19 C19 GRA 1 16.178 43.691 8.029

ATOM 20 C20 GRA 1 18.477 48.009 8.554

ATOM 21 C21 GRA 1 20.776 52.327 9.078

ATOM 22 C22 GRA 1 23.074 56.646 9.603

ATOM 23 C23 GRA 1 25.373 60.964 10.127

ATOM 24 C24 GRA 1 27.672 65.282 10.652

ATOM 25 C25 GRA 1 29.971 69.600 11.176

ATOM 26 C26 GRA 1 11.896 26.582 6.127

ATOM 27 C27 GRA 1 14.195 30.900 6.652

ATOM 28 C28 GRA 1 16.493 35.218 7.176

ATOM 29 C29 GRA 1 18.792 39.536 7.701

ATOM 30 C30 GRA 1 21.091 43.854 8.225

ATOM 31 C31 GRA 1 23.390 48.172 8.750

ATOM 32 C32 GRA 1 25.689 52.491 9.274

ATOM 33 C33 GRA 1 27.988 56.809 9.799

ATOM 34 C34 GRA 1 30.287 61.127 10.324

ATOM 35 C35 GRA 1 32.586 65.445 10.848

ATOM 36 C36 GRA 1 34.885 69.763 11.373

ATOM 1 C37 GRA 1 12.211 18.109 5.275

ATOM 38 C38 GRA 1 14.510 22.427 5.799

ATOM 39 C39 GRA 1 16.809 26.745 6.324

ATOM 40 C40 GRA 1 19.108 31.063 6.848

ATOM 41 C41 GRA 1 21.407 35.381 7.373

ATOM 42 C42 GRA 1 23.706 39.699 7.897

ATOM 43 C43 GRA 1 26.005 44.017 8.422

ATOM 44 C44 GRA 1 28.304 48.336 8.946

ATOM 45 C45 GRA 1 30.603 52.654 9.471

ATOM 46 C46 GRA 1 32.901 56.972 9.995

ATOM 47 C47 GRA 1 35.200 61.290 10.520

ATOM 48 C48 GRA 1 37.499 65.608 11.044

ATOM 49 C49 GRA 1 39.798 69.926 11.569

ATOM 50 C50 GRA 1 17.125 18.272 5.471

ATOM 51 C51 GRA 1 19.424 22.590 5.996

ATOM 52 C52 GRA 1 21.723 26.908 6.520

ATOM 53 C53 GRA 1 24.022 31.226 7.045

ATOM 54 C54 GRA 1 26.320 35.544 7.569

ATOM 55 C55 GRA 1 28.619 39.863 8.094

ATOM 56 C56 GRA 1 30.918 44.181 8.618

ATOM 57 C57 GRA 1 33.217 48.499 9.143

ATOM 58 C58 GRA 1 35.516 52.817 9.667

ATOM 59 C59 GRA 1 37.815 57.135 10.192

ATOM 60 C60 GRA 1 40.114 61.453 10.716

ATOM 61 C61 GRA 1 42.413 65.771 11.241

ATOM 62 C62 GRA 1 44.712 70.089 11.765

ATOM 63 C63 GRA 1 22.038 18.435 5.667

ATOM 64 C64 GRA 1 24.336 22.754 6.192

ATOM 65 C65 GRA 1 26.635 27.072 6.716

ATOM 66 C66 GRA 1 28.934 31.390 7.241

ATOM 67 C67 GRA 1 31.233 35.708 7.765

ATOM 68 C68 GRA 1 33.532 40.026 8.290

ATOM 69 C69 GRA 1 35.831 44.344 8.814

ATOM 70 C70 GRA 1 38.130 48.662 9.339

ATOM 71 C71 GRA 1 40.429 52.981 9.864

ATOM 72 C72 GRA 1 42.728 57.299 10.388

ATOM 73 C73 GRA 1 45.026 61.617 10.913

ATOM 74 C74 GRA 1 47.325 65.935 11.437

ATOM 75 C75 GRA 1 49.624 70.253 11.962

ATOM 76 C76 GRA 1 26.951 18.599 5.864

ATOM 77 C77 GRA 1 29.250 22.917 6.388

ATOM 78 C78 GRA 1 31.549 27.235 6.913

ATOM 79 C79 GRA 1 33.848 31.553 7.437

ATOM 80 C80 GRA 1 36.147 35.871 7.962

ATOM 81 C81 GRA 1 38.446 40.189 8.486

ATOM 82 C82 GRA 1 40.744 44.507 9.011

ATOM 83 C83 GRA 1 43.043 48.826 9.535

ATOM 84 C84 GRA 1 45.342 53.144 10.060

ATOM 85 C85 GRA 1 47.641 57.462 10.584

ATOM 86 C86 GRA 1 49.940 61.780 11.109

ATOM 87 C87 GRA 1 52.239 66.098 11.633

ATOM 88 C88 GRA 1 54.538 70.416 12.158

ATOM 89 C89 GRA 1 31.865 18.762 6.060

ATOM 90 C90 GRA 1 34.163 23.080 6.585

ATOM 91 C91 GRA 1 36.462 27.398 7.109

ATOM 92 C92 GRA 1 38.761 31.716 7.634

ATOM 93 C93 GRA 1 41.060 36.034 8.158

ATOM 94 C94 GRA 1 43.359 40.352 8.683

ATOM 95 C95 GRA 1 45.658 44.671 9.207

ATOM 96 C96 GRA 1 47.957 48.989 9.732

ATOM 97 C97 GRA 1 50.256 53.307 10.256

ATOM 98 C98 GRA 1 52.555 57.625 10.781

ATOM 99 C99 GRA 1 54.854 61.943 11.305

ATOM 100 0C10 GRA 1 57.152 66.261 11.830

ATOM 101 1C10 GRA 1 59.451 70.579 12.354

ATOM 102 2C10 GRA 1 36.778 18.925 6.256

ATOM 103 3C10 GRA 1 39.077 23.243 6.781

ATOM 104 4C10 GRA 1 41.376 27.561 7.305

ATOM 105 5C10 GRA 1 43.675 31.879 7.830

ATOM 106 6C10 GRA 1 45.974 36.197 8.354

ATOM 107 7C10 GRA 1 48.273 40.516 8.879

ATOM 108 8C10 GRA 1 50.571 44.834 9.403

ATOM 109 9C10 GRA 1 52.870 49.152 9.928

ATOM 110 0C11 GRA 1 55.169 53.470 10.453

ATOM 111 1C11 GRA 1 57.468 57.788 10.977

ATOM 112 2C11 GRA 1 59.767 62.106 11.502

ATOM 113 3C11 GRA 1 62.066 66.424 12.026

ATOM 114 4C11 GRA 1 64.365 70.743 12.551

ATOM 115 5C11 GRA 1 41.692 19.088 6.453

ATOM 116 6C11 GRA 1 43.990 23.406 6.977

ATOM 117 7C11 GRA 1 46.289 27.724 7.502

ATOM 118 8C11 GRA 1 48.588 32.043 8.026

ATOM 119 9C11 GRA 1 50.887 36.361 8.551

ATOM 120 0C12 GRA 1 53.186 40.679 9.075

ATOM 121 1C12 GRA 1 55.485 44.997 9.600

ATOM 122 2C12 GRA 1 57.784 49.315 10.124

ATOM 123 3C12 GRA 1 60.083 53.633 10.649

ATOM 124 4C12 GRA 1 62.382 57.951 11.173

ATOM 125 5C12 GRA 1 64.681 62.269 11.698

ATOM 126 6C12 GRA 1 66.979 66.588 12.222

ATOM 127 7C12 GRA 1 69.278 70.906 12.747

ATOM 128 8C12 GRA 1 46.605 19.251 6.649

ATOM 129 9C12 GRA 1 48.904 23.569 7.174

ATOM 130 0C13 GRA 1 51.203 27.888 7.698

ATOM 131 1C13 GRA 1 53.502 32.206 8.223

ATOM 132 2C13 GRA 1 55.801 36.524 8.747

ATOM 133 3C13 GRA 1 58.100 40.842 9.272

ATOM 134 4C13 GRA 1 60.398 45.160 9.796

ATOM 135 5C13 GRA 1 62.697 49.478 10.321

ATOM 136 6C13 GRA 1 64.996 53.796 10.845

ATOM 137 7C13 GRA 1 67.295 58.115 11.370

ATOM 138 8C13 GRA 1 69.594 62.433 11.894

ATOM 139 9C13 GRA 1 71.893 66.751 12.419

ATOM 140 0C14 GRA 1 74.192 71.069 12.943

ATOM 141 1C14 GRA 1 51.519 19.414 6.845

ATOM 142 2C14 GRA 1 53.817 23.733 7.370

ATOM 143 3C14 GRA 1 56.116 28.051 7.894

ATOM 144 4C14 GRA 1 58.415 32.369 8.419

ATOM 145 5C14 GRA 1 60.714 36.687 8.943

ATOM 146 6C14 GRA 1 63.013 41.005 9.468

ATOM 147 7C14 GRA 1 65.312 45.323 9.992

ATOM 148 8C14 GRA 1 67.611 49.641 10.517

ATOM 149 9C14 GRA 1 69.910 53.960 11.042

ATOM 150 0C15 GRA 1 72.209 58.278 11.566

ATOM 151 1C15 GRA 1 74.508 62.596 12.091

ATOM 152 2C15 GRA 1 76.806 66.914 12.615

ATOM 153 3C15 GRA 1 79.105 71.232 13.140

ATOM 154 4C15 GRA 1 56.431 19.578 7.042

ATOM 155 5C15 GRA 1 58.730 23.896 7.566

ATOM 156 6C15 GRA 1 61.029 28.214 8.091

ATOM 157 7C15 GRA 1 63.328 32.532 8.615

ATOM 158 8C15 GRA 1 65.627 36.851 9.140

ATOM 159 9C15 GRA 1 67.926 41.169 9.664

ATOM 160 0C16 GRA 1 70.225 45.487 10.189

ATOM 161 1C16 GRA 1 72.523 49.805 10.713

ATOM 162 2C16 GRA 1 74.822 54.123 11.238

ATOM 163 3C16 GRA 1 77.121 58.441 11.762

ATOM 164 4C16 GRA 1 79.420 62.759 12.287

ATOM 165 5C16 GRA 1 81.719 67.078 12.811

ATOM 166 6C16 GRA 1 61.345 19.741 7.238

ATOM 167 7C16 GRA 1 63.644 24.059 7.763

ATOM 168 8C16 GRA 1 65.942 28.377 8.287

ATOM 169 9C16 GRA 1 68.241 32.696 8.812

ATOM 170 0C17 GRA 1 70.540 37.014 9.336

ATOM 171 1C17 GRA 1 72.839 41.332 9.861

ATOM 172 2C17 GRA 1 75.138 45.650 10.385

ATOM 173 3C17 GRA 1 77.437 49.968 10.910

ATOM 174 4C17 GRA 1 79.736 54.286 11.434

ATOM 175 5C17 GRA 1 82.035 58.604 11.959

ATOM 176 6C17 GRA 1 66.258 19.904 7.434

ATOM 177 7C17 GRA 1 68.557 24.223 7.959

ATOM 178 8C17 GRA 1 70.856 28.541 8.483

ATOM 179 9C17 GRA 1 73.155 32.859 9.008

ATOM 180 0C18 GRA 1 75.454 37.177 9.532

ATOM 181 1C18 GRA 1 77.753 41.495 10.057

ATOM 182 2C18 GRA 1 80.052 45.813 10.582

ATOM 183 3C18 GRA 1 82.350 50.131 11.106

ATOM 184 4C18 GRA 1 71.172 20.068 7.631

ATOM 185 5C18 GRA 1 73.471 24.386 8.155

ATOM 186 6C18 GRA 1 75.769 28.704 8.680

ATOM 187 7C18 GRA 1 78.068 33.022 9.204

ATOM 188 8C18 GRA 1 80.367 37.340 9.729

ATOM 189 9C18 GRA 1 82.666 41.658 10.253

ATOM 190 0C19 GRA 1 76.085 20.231 7.827

ATOM 191 1C19 GRA 1 78.384 24.549 8.352

ATOM 192 2C19 GRA 1 80.683 28.867 8.876

ATOM 193 3C19 GRA 1 82.982 33.185 9.401

ATOM 194 4C19 GRA 1 80.999 20.394 8.023

ATOM 195 5C19 GRA 1 83.298 24.712 8.548

ATOM 196 6C19 GRA 1 11.783 62.633 9.800

ATOM 197 7C19 GRA 1 14.082 66.951 10.325

ATOM 198 8C19 GRA 1 12.098 54.160 8.948

ATOM 199 9C19 GRA 1 14.397 58.478 9.472

ATOM 200 0C20 GRA 1 16.696 62.796 9.997

ATOM 201 1C20 GRA 1 18.995 67.115 10.521

ATOM 202 2C20 GRA 1 12.414 45.687 8.095

ATOM 203 3C20 GRA 1 14.713 50.005 8.620

ATOM 204 4C20 GRA 1 17.011 54.323 9.144

ATOM 205 5C20 GRA 1 19.310 58.641 9.669

ATOM 206 6C20 GRA 1 21.609 62.960 10.193

ATOM 207 7C20 GRA 1 23.908 67.278 10.718

ATOM 208 8C20 GRA 1 12.729 37.214 7.242

ATOM 209 9C20 GRA 1 15.028 41.532 7.767

ATOM 210 0C21 GRA 1 17.327 45.850 8.291

ATOM 211 1C21 GRA 1 19.626 50.168 8.816

ATOM 212 2C21 GRA 1 21.925 54.487 9.340

ATOM 213 3C21 GRA 1 24.224 58.805 9.865

ATOM 214 4C21 GRA 1 26.523 63.123 10.389

ATOM 215 5C21 GRA 1 28.822 67.441 10.914

ATOM 216 6C21 GRA 1 13.045 28.741 6.390

ATOM 217 7C21 GRA 1 15.344 33.059 6.914

ATOM 218 8C21 GRA 1 17.643 37.377 7.439

ATOM 219 9C21 GRA 1 19.942 41.695 7.963

ATOM 220 0C22 GRA 1 22.241 46.013 8.488

ATOM 221 1C22 GRA 1 24.540 50.332 9.012

ATOM 222 2C22 GRA 1 26.839 54.650 9.537

ATOM 223 3C22 GRA 1 29.137 58.968 10.061

ATOM 224 4C22 GRA 1 31.436 63.286 10.586

ATOM 225 5C22 GRA 1 33.735 67.604 11.110

ATOM 226 6C22 GRA 1 13.361 20.268 5.537

ATOM 227 7C22 GRA 1 15.660 24.586 6.061

ATOM 228 8C22 GRA 1 17.959 28.904 6.586

ATOM 229 9C22 GRA 1 20.258 33.222 7.111

ATOM 230 0C23 GRA 1 22.556 37.540 7.635

ATOM 231 1C23 GRA 1 24.855 41.858 8.160

ATOM 232 2C23 GRA 1 27.154 46.177 8.684

ATOM 233 3C23 GRA 1 29.453 50.495 9.209

ATOM 234 4C23 GRA 1 31.752 54.813 9.733

ATOM 235 5C23 GRA 1 34.051 59.131 10.258

ATOM 236 6C23 GRA 1 36.350 63.449 10.782

ATOM 237 7C23 GRA 1 38.649 67.767 11.307

ATOM 238 8C23 GRA 1 15.975 16.113 5.209

ATOM 239 9C23 GRA 1 18.274 20.431 5.733

ATOM 240 0C24 GRA 1 20.573 24.749 6.258

ATOM 241 1C24 GRA 1 22.872 29.067 6.782

ATOM 242 2C24 GRA 1 25.171 33.385 7.307

ATOM 243 3C24 GRA 1 27.470 37.703 7.831

ATOM 244 4C24 GRA 1 29.769 42.022 8.356

ATOM 245 5C24 GRA 1 32.068 46.340 8.880

ATOM 246 6C24 GRA 1 34.367 50.658 9.405

ATOM 247 7C24 GRA 1 36.666 54.976 9.929

ATOM 248 8C24 GRA 1 38.964 59.294 10.454

ATOM 249 9C24 GRA 1 41.263 63.612 10.978

ATOM 250 0C25 GRA 1 43.562 67.930 11.503

ATOM 251 1C25 GRA 1 20.888 16.276 5.405

ATOM 252 2C25 GRA 1 23.187 20.594 5.930

ATOM 253 3C25 GRA 1 25.486 24.913 6.454

ATOM 254 4C25 GRA 1 27.785 29.231 6.979

ATOM 255 5C25 GRA 1 30.084 33.549 7.503

ATOM 256 6C25 GRA 1 32.383 37.867 8.028

ATOM 257 7C25 GRA 1 34.681 42.185 8.552

ATOM 258 8C25 GRA 1 36.980 46.503 9.077

ATOM 259 9C25 GRA 1 39.279 50.821 9.601

ATOM 260 0C26 GRA 1 41.578 55.140 10.126

ATOM 261 1C26 GRA 1 43.877 59.458 10.650

ATOM 262 2C26 GRA 1 46.176 63.776 11.175

ATOM 263 3C26 GRA 1 48.475 68.094 11.699

ATOM 264 4C26 GRA 1 25.802 16.440 5.601

ATOM 265 5C26 GRA 1 28.100 20.758 6.126

ATOM 266 6C26 GRA 1 30.399 25.076 6.651

ATOM 267 7C26 GRA 1 32.698 29.394 7.175

ATOM 268 8C26 GRA 1 34.997 33.712 7.700

ATOM 269 9C26 GRA 1 37.296 38.030 8.224

ATOM 270 0C27 GRA 1 39.595 42.348 8.749

ATOM 271 1C27 GRA 1 41.894 46.667 9.273

ATOM 272 2C27 GRA 1 44.193 50.985 9.798

ATOM 273 3C27 GRA 1 46.492 55.303 10.322

ATOM 274 4C27 GRA 1 48.791 59.621 10.847

ATOM 275 5C27 GRA 1 51.089 63.939 11.371

ATOM 276 6C27 GRA 1 53.388 68.257 11.896

ATOM 277 7C27 GRA 1 30.715 16.603 5.798

ATOM 278 8C27 GRA 1 33.014 20.921 6.322

ATOM 279 9C27 GRA 1 35.313 25.239 6.847

ATOM 280 0C28 GRA 1 37.612 29.557 7.371

ATOM 281 1C28 GRA 1 39.911 33.875 7.896

ATOM 282 2C28 GRA 1 42.210 38.193 8.420

ATOM 283 3C28 GRA 1 44.508 42.512 8.945

ATOM 284 4C28 GRA 1 46.807 46.830 9.469

ATOM 285 5C28 GRA 1 49.106 51.148 9.994

ATOM 286 6C28 GRA 1 51.405 55.466 10.518

ATOM 287 7C28 GRA 1 53.704 59.784 11.043

ATOM 288 8C28 GRA 1 56.003 64.102 11.567

ATOM 289 9C28 GRA 1 58.302 68.420 12.092

ATOM 290 0C29 GRA 1 35.629 16.766 5.994

ATOM 291 1C29 GRA 1 37.927 21.084 6.519

ATOM 292 2C29 GRA 1 40.226 25.402 7.043

ATOM 293 3C29 GRA 1 42.525 29.720 7.568

ATOM 294 4C29 GRA 1 44.824 34.038 8.092

ATOM 295 5C29 GRA 1 47.123 38.357 8.617

ATOM 296 6C29 GRA 1 49.422 42.675 9.141

ATOM 297 7C29 GRA 1 51.721 46.993 9.666

ATOM 298 8C29 GRA 1 54.020 51.311 10.190

ATOM 299 9C29 GRA 1 56.319 55.629 10.715

ATOM 300 0C30 GRA 1 58.618 59.947 11.239

ATOM 301 1C30 GRA 1 60.916 64.265 11.764

ATOM 302 2C30 GRA 1 63.215 68.584 12.288

ATOM 303 3C30 GRA 1 40.542 16.929 6.191

ATOM 304 4C30 GRA 1 42.841 21.247 6.715

ATOM 305 5C30 GRA 1 45.140 25.565 7.240

ATOM 306 6C30 GRA 1 47.439 29.883 7.764

ATOM 307 7C30 GRA 1 49.738 34.202 8.289

ATOM 308 8C30 GRA 1 52.037 38.520 8.813

ATOM 309 9C30 GRA 1 54.335 42.838 9.338

ATOM 310 0C31 GRA 1 56.634 47.156 9.862

ATOM 311 1C31 GRA 1 58.933 51.474 10.387

ATOM 312 2C31 GRA 1 61.232 55.792 10.911

ATOM 313 3C31 GRA 1 63.531 60.110 11.436

ATOM 314 4C31 GRA 1 65.830 64.429 11.960

ATOM 315 5C31 GRA 1 68.129 68.747 12.485

ATOM 316 6C31 GRA 1 45.456 17.092 6.387

ATOM 317 7C31 GRA 1 47.754 21.410 6.911

ATOM 318 8C31 GRA 1 50.053 25.728 7.436

ATOM 319 9C31 GRA 1 52.352 30.047 7.960

ATOM 320 0C32 GRA 1 54.651 34.365 8.485

ATOM 321 1C32 GRA 1 56.950 38.683 9.009

ATOM 322 2C32 GRA 1 59.249 43.001 9.534

ATOM 323 3C32 GRA 1 61.548 47.319 10.058

ATOM 324 4C32 GRA 1 63.847 51.637 10.583

ATOM 325 5C32 GRA 1 66.146 55.955 11.107

ATOM 326 6C32 GRA 1 68.445 60.274 11.632

ATOM 327 7C32 GRA 1 70.743 64.592 12.156

ATOM 328 8C32 GRA 1 73.042 68.910 12.681

ATOM 329 9C32 GRA 1 50.369 17.255 6.583

ATOM 330 0C33 GRA 1 52.668 21.573 7.108

ATOM 331 1C33 GRA 1 54.967 25.892 7.632

ATOM 332 2C33 GRA 1 57.266 30.210 8.157

ATOM 333 3C33 GRA 1 59.565 34.528 8.681

ATOM 334 4C33 GRA 1 61.864 38.846 9.206

ATOM 335 5C33 GRA 1 64.162 43.164 9.730

ATOM 336 6C33 GRA 1 66.461 47.482 10.255

ATOM 337 7C33 GRA 1 68.760 51.800 10.779

ATOM 338 8C33 GRA 1 71.059 56.119 11.304

ATOM 339 9C33 GRA 1 73.358 60.437 11.828

ATOM 340 0C34 GRA 1 75.657 64.755 12.353

ATOM 341 1C34 GRA 1 77.956 69.073 12.877

ATOM 342 2C34 GRA 1 55.282 17.419 6.780

ATOM 343 3C34 GRA 1 57.581 21.737 7.304

ATOM 344 4C34 GRA 1 59.880 26.055 7.829

ATOM 345 5C34 GRA 1 62.178 30.373 8.353

ATOM 346 6C34 GRA 1 64.477 34.692 8.878

ATOM 347 7C34 GRA 1 66.776 39.010 9.402

ATOM 348 8C34 GRA 1 69.075 43.328 9.927

ATOM 349 9C34 GRA 1 71.374 47.646 10.451

ATOM 350 0C35 GRA 1 73.673 51.964 10.976

ATOM 351 1C35 GRA 1 75.972 56.282 11.500

ATOM 352 2C35 GRA 1 78.271 60.600 12.025

ATOM 353 3C35 GRA 1 80.570 64.919 12.549

ATOM 354 4C35 GRA 1 60.195 17.582 6.976

ATOM 355 5C35 GRA 1 62.494 21.900 7.500

ATOM 356 6C35 GRA 1 64.793 26.218 8.025

ATOM 357 7C35 GRA 1 67.092 30.537 8.549

ATOM 358 8C35 GRA 1 69.391 34.855 9.074

ATOM 359 9C35 GRA 1 71.690 39.173 9.598

ATOM 360 0C36 GRA 1 73.989 43.491 10.123

ATOM 361 1C36 GRA 1 76.288 47.809 10.647

ATOM 362 2C36 GRA 1 78.586 52.127 11.172

ATOM 363 3C36 GRA 1 80.885 56.445 11.696

ATOM 364 4C36 GRA 1 65.109 17.745 7.172

ATOM 365 5C36 GRA 1 67.408 22.063 7.697

ATOM 366 6C36 GRA 1 69.707 26.382 8.221

ATOM 367 7C36 GRA 1 72.005 30.700 8.746

ATOM 368 8C36 GRA 1 74.304 35.018 9.270

ATOM 369 9C36 GRA 1 76.603 39.336 9.795

ATOM 370 0C37 GRA 1 78.902 43.654 10.319

ATOM 371 1C37 GRA 1 81.201 47.972 10.844

ATOM 372 2C37 GRA 1 70.022 17.908 7.369

ATOM 373 3C37 GRA 1 72.321 22.227 7.893

ATOM 374 4C37 GRA 1 74.620 26.545 8.418

ATOM 375 5C37 GRA 1 76.919 30.863 8.942

ATOM 376 6C37 GRA 1 79.218 35.181 9.467

ATOM 377 7C37 GRA 1 81.517 39.499 9.991

ATOM 378 8C37 GRA 1 74.936 18.072 7.565

ATOM 379 9C37 GRA 1 77.235 22.390 8.089

ATOM 380 0C38 GRA 1 79.534 26.708 8.614

ATOM 381 1C38 GRA 1 81.832 31.026 9.138

ATOM 382 2C38 GRA 1 79.849 18.235 7.761

ATOM 383 3C38 GRA 1 82.148 22.553 8.286

ATOM 384 4C38 GRA 1 11.625 66.870 10.227

ATOM 385 5C38 GRA 1 11.941 58.397 9.374

ATOM 386 6C38 GRA 1 14.239 62.715 9.899

ATOM 387 7C38 GRA 1 16.538 67.033 10.423

ATOM 388 8C38 GRA 1 12.256 49.923 8.521

ATOM 389 9C38 GRA 1 14.555 54.242 9.046

ATOM 390 0C39 GRA 1 16.854 58.560 9.570

ATOM 391 1C39 GRA 1 19.153 62.878 10.095

ATOM 392 2C39 GRA 1 21.452 67.196 10.619

ATOM 393 3C39 GRA 1 12.572 41.450 7.669

ATOM 394 4C39 GRA 1 14.871 45.768 8.193

ATOM 395 5C39 GRA 1 17.170 50.087 8.718

ATOM 396 6C39 GRA 1 19.469 54.405 9.242

ATOM 397 7C39 GRA 1 21.768 58.723 9.767

ATOM 398 8C39 GRA 1 24.066 63.041 10.291

ATOM 399 9C39 GRA 1 26.365 67.359 10.816

ATOM 400 0C40 GRA 1 12.888 32.977 6.816

ATOM 401 1C40 GRA 1 15.187 37.295 7.341

ATOM 402 2C40 GRA 1 17.486 41.613 7.865

ATOM 403 3C40 GRA 1 19.784 45.932 8.390

ATOM 404 4C40 GRA 1 22.083 50.250 8.914

ATOM 405 5C40 GRA 1 24.382 54.568 9.439

ATOM 406 6C40 GRA 1 26.681 58.886 9.963

ATOM 407 7C40 GRA 1 28.980 63.204 10.488

ATOM 408 8C40 GRA 1 31.279 67.522 11.012

ATOM 409 9C40 GRA 1 13.203 24.504 5.963

ATOM 410 0C41 GRA 1 15.501 28.823 6.488

ATOM 411 1C41 GRA 1 17.800 33.141 7.012

ATOM 412 2C41 GRA 1 20.099 37.459 7.537

ATOM 413 3C41 GRA 1 22.398 41.777 8.061

ATOM 414 4C41 GRA 1 24.697 46.095 8.586

ATOM 415 5C41 GRA 1 26.996 50.413 9.110

ATOM 416 6C41 GRA 1 29.295 54.731 9.635

ATOM 417 7C41 GRA 1 31.594 59.050 10.159

ATOM 418 8C41 GRA 1 33.893 63.368 10.684

ATOM 419 9C41 GRA 1 36.192 67.686 11.208

ATOM 420 0C42 GRA 1 13.518 16.031 5.111

ATOM 421 1C42 GRA 1 15.817 20.350 5.635

ATOM 422 2C42 GRA 1 18.116 24.668 6.160

ATOM 423 3C42 GRA 1 20.415 28.986 6.684

ATOM 424 4C42 GRA 1 22.714 33.304 7.209

ATOM 425 5C42 GRA 1 25.013 37.622 7.733

ATOM 426 6C42 GRA 1 27.312 41.940 8.258

ATOM 427 7C42 GRA 1 29.611 46.258 8.782

ATOM 428 8C42 GRA 1 31.909 50.577 9.307

ATOM 429 9C42 GRA 1 34.208 54.895 9.831

ATOM 430 0C43 GRA 1 36.507 59.213 10.356

ATOM 431 1C43 GRA 1 38.806 63.531 10.880

ATOM 432 2C43 GRA 1 41.105 67.849 11.405

ATOM 433 3C43 GRA 1 18.432 16.195 5.307

ATOM 434 4C43 GRA 1 20.731 20.513 5.831

ATOM 435 5C43 GRA 1 23.030 24.831 6.356

ATOM 436 6C43 GRA 1 25.328 29.149 6.881

ATOM 437 7C43 GRA 1 27.627 33.467 7.405

ATOM 438 8C43 GRA 1 29.926 37.785 7.930

ATOM 439 9C43 GRA 1 32.225 42.103 8.454

ATOM 440 0C44 GRA 1 34.524 46.422 8.979

ATOM 441 1C44 GRA 1 36.823 50.740 9.503

ATOM 442 2C44 GRA 1 39.122 55.058 10.028

ATOM 443 3C44 GRA 1 41.421 59.376 10.552

ATOM 444 4C44 GRA 1 43.720 63.694 11.077

ATOM 445 5C44 GRA 1 46.019 68.012 11.601

ATOM 446 6C44 GRA 1 23.345 16.358 5.503

ATOM 447 7C44 GRA 1 25.644 20.676 6.028

ATOM 448 8C44 GRA 1 27.943 24.994 6.552

ATOM 449 9C44 GRA 1 30.242 29.312 7.077

ATOM 450 0C45 GRA 1 32.541 33.630 7.601

ATOM 451 1C45 GRA 1 34.840 37.948 8.126

ATOM 452 2C45 GRA 1 37.139 42.267 8.650

ATOM 453 3C45 GRA 1 39.438 46.585 9.175

ATOM 454 4C45 GRA 1 41.736 50.903 9.699

ATOM 455 5C45 GRA 1 44.035 55.221 10.224

ATOM 456 6C45 GRA 1 46.334 59.539 10.748

ATOM 457 7C45 GRA 1 48.633 63.857 11.273

ATOM 458 8C45 GRA 1 50.932 68.175 11.797

ATOM 459 9C45 GRA 1 28.259 16.521 5.700

ATOM 460 0C46 GRA 1 30.558 20.839 6.224

ATOM 461 1C46 GRA 1 32.857 25.157 6.749

ATOM 462 2C46 GRA 1 35.155 29.475 7.273

ATOM 463 3C46 GRA 1 37.454 33.793 7.798

ATOM 464 4C46 GRA 1 39.753 38.112 8.322

ATOM 465 5C46 GRA 1 42.052 42.430 8.847

ATOM 466 6C46 GRA 1 44.351 46.748 9.371

ATOM 467 7C46 GRA 1 46.650 51.066 9.896

ATOM 468 8C46 GRA 1 48.949 55.384 10.420

ATOM 469 9C46 GRA 1 51.248 59.702 10.945

ATOM 470 0C47 GRA 1 53.547 64.020 11.469

ATOM 471 1C47 GRA 1 55.846 68.339 11.994

ATOM 472 2C47 GRA 1 33.172 16.684 5.896

ATOM 473 3C47 GRA 1 35.471 21.002 6.421

ATOM 474 4C47 GRA 1 37.770 25.320 6.945

ATOM 475 5C47 GRA 1 40.069 29.638 7.470

ATOM 476 6C47 GRA 1 42.368 33.957 7.994

ATOM 477 7C47 GRA 1 44.667 38.275 8.519

ATOM 478 8C47 GRA 1 46.966 42.593 9.043

ATOM 479 9C47 GRA 1 49.265 46.911 9.568

ATOM 480 0C48 GRA 1 51.563 51.229 10.092

ATOM 481 1C48 GRA 1 53.862 55.547 10.617

ATOM 482 2C48 GRA 1 56.161 59.865 11.141

ATOM 483 3C48 GRA 1 58.460 64.184 11.666

ATOM 484 4C48 GRA 1 60.759 68.502 12.190

ATOM 485 5C48 GRA 1 38.085 16.848 6.092

ATOM 486 6C48 GRA 1 40.384 21.166 6.617

ATOM 487 7C48 GRA 1 42.683 25.484 7.141

ATOM 488 8C48 GRA 1 44.982 29.802 7.666

ATOM 489 9C48 GRA 1 47.280 34.120 8.190

ATOM 490 0C49 GRA 1 49.579 38.438 8.715

ATOM 491 1C49 GRA 1 51.878 42.757 9.239

ATOM 492 2C49 GRA 1 54.177 47.075 9.764

ATOM 493 3C49 GRA 1 56.476 51.393 10.288

ATOM 494 4C49 GRA 1 58.775 55.711 10.813

ATOM 495 5C49 GRA 1 61.074 60.029 11.337

ATOM 496 6C49 GRA 1 63.373 64.347 11.862

ATOM 497 7C49 GRA 1 65.672 68.665 12.386

ATOM 498 8C49 GRA 1 42.998 17.011 6.289

ATOM 499 9C49 GRA 1 45.297 21.329 6.813

ATOM 500 0C50 GRA 1 47.596 25.647 7.338

ATOM 501 1C50 GRA 1 49.895 29.965 7.862

ATOM 502 2C50 GRA 1 52.194 34.283 8.387

ATOM 503 3C50 GRA 1 54.493 38.602 8.911

ATOM 504 4C50 GRA 1 56.792 42.920 9.436

ATOM 505 5C50 GRA 1 59.091 47.238 9.960

ATOM 506 6C50 GRA 1 61.390 51.556 10.485

ATOM 507 7C50 GRA 1 63.688 55.874 11.009

ATOM 508 8C50 GRA 1 65.987 60.192 11.534

ATOM 509 9C50 GRA 1 68.286 64.510 12.058

ATOM 510 0C51 GRA 1 70.585 68.829 12.583

ATOM 511 1C51 GRA 1 47.912 17.174 6.485

ATOM 512 2C51 GRA 1 50.211 21.492 7.010

ATOM 513 3C51 GRA 1 52.510 25.810 7.534

ATOM 514 4C51 GRA 1 54.809 30.128 8.059

ATOM 515 5C51 GRA 1 57.108 34.447 8.583

ATOM 516 6C51 GRA 1 59.406 38.765 9.108

ATOM 517 7C51 GRA 1 61.705 43.083 9.632

ATOM 518 8C51 GRA 1 64.004 47.401 10.157

ATOM 519 9C51 GRA 1 66.303 51.719 10.681

ATOM 520 0C52 GRA 1 68.602 56.037 11.206

ATOM 521 1C52 GRA 1 70.901 60.355 11.730

ATOM 522 2C52 GRA 1 73.200 64.674 12.255

ATOM 523 3C52 GRA 1 75.499 68.992 12.779

ATOM 524 4C52 GRA 1 52.825 17.337 6.681

ATOM 525 5C52 GRA 1 55.124 21.655 7.206

ATOM 526 6C52 GRA 1 57.423 25.973 7.730

ATOM 527 7C52 GRA 1 59.722 30.292 8.255

ATOM 528 8C52 GRA 1 62.021 34.610 8.779

ATOM 529 9C52 GRA 1 64.320 38.928 9.304

ATOM 530 0C53 GRA 1 66.619 43.246 9.828

ATOM 531 1C53 GRA 1 68.918 47.564 10.353

ATOM 532 2C53 GRA 1 71.217 51.882 10.877

ATOM 533 3C53 GRA 1 73.516 56.200 11.402

ATOM 534 4C53 GRA 1 75.814 60.519 11.926

ATOM 535 5C53 GRA 1 78.113 64.837 12.451

ATOM 536 6C53 GRA 1 80.412 69.155 12.975

ATOM 537 7C53 GRA 1 57.739 17.500 6.878

ATOM 538 8C53 GRA 1 60.038 21.818 7.402

ATOM 539 9C53 GRA 1 62.337 26.137 7.927

ATOM 540 0C54 GRA 1 64.636 30.455 8.451

ATOM 541 1C54 GRA 1 66.935 34.773 8.976

ATOM 542 2C54 GRA 1 69.233 39.091 9.500

ATOM 543 3C54 GRA 1 71.532 43.409 10.025

ATOM 544 4C54 GRA 1 73.831 47.727 10.549

ATOM 545 5C54 GRA 1 76.130 52.045 11.074

ATOM 546 6C54 GRA 1 78.429 56.364 11.598

ATOM 547 7C54 GRA 1 80.728 60.682 12.123

ATOM 548 8C54 GRA 1 62.652 17.663 7.074

ATOM 549 9C54 GRA 1 64.951 21.982 7.599

ATOM 550 0C55 GRA 1 67.250 26.300 8.123

ATOM 551 1C55 GRA 1 69.549 30.618 8.648

ATOM 552 2C55 GRA 1 71.848 34.936 9.172

ATOM 553 3C55 GRA 1 74.147 39.254 9.697

ATOM 554 4C55 GRA 1 76.446 43.572 10.221

ATOM 555 5C55 GRA 1 78.745 47.890 10.746

ATOM 556 6C55 GRA 1 81.044 52.209 11.270

ATOM 557 7C55 GRA 1 67.565 17.827 7.270

ATOM 558 8C55 GRA 1 69.864 22.145 7.795

ATOM 559 9C55 GRA 1 72.163 26.463 8.319

ATOM 560 0C56 GRA 1 74.462 30.782 8.844

ATOM 561 1C56 GRA 1 76.761 35.100 9.368

ATOM 562 2C56 GRA 1 79.060 39.418 9.893

ATOM 563 3C56 GRA 1 81.358 43.736 10.417

ATOM 564 4C56 GRA 1 72.479 17.990 7.467

ATOM 565 5C56 GRA 1 74.777 22.308 7.991

ATOM 566 6C56 GRA 1 77.076 26.627 8.516

ATOM 567 7C56 GRA 1 79.375 30.945 9.040

ATOM 568 8C56 GRA 1 81.674 35.263 9.565

ATOM 569 9C56 GRA 1 77.392 18.153 7.663

ATOM 570 0C57 GRA 1 79.691 22.472 8.188

ATOM 571 1C57 GRA 1 81.990 26.790 8.712

ATOM 572 2C57 GRA 1 82.306 18.317 7.859

ATOM 573 3C57 GRA 1 10.475 64.711 9.965

ATOM 574 4C57 GRA 1 12.774 69.029 10.489

ATOM 575 5C57 GRA 1 10.791 56.237 9.112

ATOM 576 6C57 GRA 1 13.090 60.556 9.636

ATOM 577 7C57 GRA 1 15.389 64.874 10.161

ATOM 578 8C57 GRA 1 17.688 69.192 10.685

ATOM 579 9C57 GRA 1 11.107 47.764 8.259

ATOM 580 0C58 GRA 1 13.406 52.082 8.784

ATOM 581 1C58 GRA 1 15.705 56.401 9.308

ATOM 582 2C58 GRA 1 18.004 60.719 9.833

ATOM 583 3C58 GRA 1 20.302 65.037 10.357

ATOM 584 4C58 GRA 1 22.601 69.355 10.882

ATOM 585 5C58 GRA 1 11.423 39.291 7.406

ATOM 586 6C58 GRA 1 13.721 43.609 7.931

ATOM 587 7C58 GRA 1 16.020 47.927 8.455

ATOM 588 8C58 GRA 1 18.319 52.246 8.980

ATOM 589 9C58 GRA 1 20.618 56.564 9.504

ATOM 590 0C59 GRA 1 22.917 60.882 10.029

ATOM 591 1C59 GRA 1 25.216 65.200 10.554

ATOM 592 2C59 GRA 1 27.515 69.518 11.078

ATOM 593 3C59 GRA 1 11.738 30.818 6.554

ATOM 594 4C59 GRA 1 14.037 35.136 7.078

ATOM 595 5C59 GRA 1 16.336 39.454 7.603

ATOM 596 6C59 GRA 1 18.635 43.773 8.127

ATOM 597 7C59 GRA 1 20.934 48.091 8.652

ATOM 598 8C59 GRA 1 23.233 52.409 9.176

ATOM 599 9C59 GRA 1 25.532 56.727 9.701

ATOM 600 0C60 GRA 1 27.831 61.045 10.225

ATOM 601 1C60 GRA 1 30.129 65.363 10.750

ATOM 602 2C60 GRA 1 32.428 69.681 11.274

ATOM 603 3C60 GRA 1 12.053 22.345 5.701

ATOM 604 4C60 GRA 1 14.352 26.664 6.226

ATOM 605 5C60 GRA 1 16.651 30.982 6.750

ATOM 606 6C60 GRA 1 18.950 35.300 7.275

ATOM 607 7C60 GRA 1 21.249 39.618 7.799

ATOM 608 8C60 GRA 1 23.548 43.936 8.324

ATOM 609 9C60 GRA 1 25.846 48.254 8.848

ATOM 610 0C61 GRA 1 28.145 52.572 9.373

ATOM 611 1C61 GRA 1 30.444 56.891 9.897

ATOM 612 2C61 GRA 1 32.743 61.209 10.422

ATOM 613 3C61 GRA 1 35.042 65.527 10.946

ATOM 614 4C61 GRA 1 37.341 69.845 11.471

ATOM 615 5C61 GRA 1 14.668 18.190 5.373

ATOM 616 6C61 GRA 1 16.967 22.509 5.897

ATOM 617 7C61 GRA 1 19.265 26.827 6.422

ATOM 618 8C61 GRA 1 21.564 31.145 6.946

ATOM 619 9C61 GRA 1 23.863 35.463 7.471

ATOM 620 0C62 GRA 1 26.162 39.781 7.995

ATOM 621 1C62 GRA 1 28.461 44.099 8.520

ATOM 622 2C62 GRA 1 30.760 48.417 9.044

ATOM 623 3C62 GRA 1 33.059 52.736 9.569

ATOM 624 4C62 GRA 1 35.358 57.054 10.094

ATOM 625 5C62 GRA 1 37.657 61.372 10.618

ATOM 626 6C62 GRA 1 39.956 65.690 11.143

ATOM 627 7C62 GRA 1 42.254 70.008 11.667

ATOM 628 8C62 GRA 1 19.581 18.354 5.569

ATOM 629 9C62 GRA 1 21.880 22.672 6.094

ATOM 630 0C63 GRA 1 24.179 26.990 6.618

ATOM 631 1C63 GRA 1 26.478 31.308 7.143

ATOM 632 2C63 GRA 1 28.777 35.626 7.667

ATOM 633 3C63 GRA 1 31.076 39.944 8.192

ATOM 634 4C63 GRA 1 33.375 44.262 8.716

ATOM 635 5C63 GRA 1 35.673 48.581 9.241

ATOM 636 6C63 GRA 1 37.972 52.899 9.765

ATOM 637 7C63 GRA 1 40.271 57.217 10.290

ATOM 638 8C63 GRA 1 42.570 61.535 10.814

ATOM 639 9C63 GRA 1 44.869 65.853 11.339

ATOM 640 0C64 GRA 1 47.168 70.171 11.863

ATOM 641 1C64 GRA 1 24.495 18.517 5.766

ATOM 642 2C64 GRA 1 26.794 22.835 6.290

ATOM 643 3C64 GRA 1 29.092 27.153 6.815

ATOM 644 4C64 GRA 1 31.391 31.471 7.339

ATOM 645 5C64 GRA 1 33.690 35.789 7.864

ATOM 646 6C64 GRA 1 35.989 40.107 8.388

ATOM 647 7C64 GRA 1 38.288 44.426 8.913

ATOM 648 8C64 GRA 1 40.587 48.744 9.437

ATOM 649 9C64 GRA 1 42.886 53.062 9.962

ATOM 650 0C65 GRA 1 45.185 57.380 10.486

ATOM 651 1C65 GRA 1 47.484 61.698 11.011

ATOM 652 2C65 GRA 1 49.783 66.016 11.535

ATOM 653 3C65 GRA 1 52.081 70.334 12.060

ATOM 654 4C65 GRA 1 29.408 18.680 5.962

ATOM 655 5C65 GRA 1 31.707 22.998 6.486

ATOM 656 6C65 GRA 1 34.006 27.316 7.011

ATOM 657 7C65 GRA 1 36.305 31.634 7.535

ATOM 658 8C65 GRA 1 38.604 35.953 8.060

ATOM 659 9C65 GRA 1 40.903 40.271 8.584

ATOM 660 0C66 GRA 1 43.202 44.589 9.109

ATOM 661 1C66 GRA 1 45.501 48.907 9.633

ATOM 662 2C66 GRA 1 47.799 53.225 10.158

ATOM 663 3C66 GRA 1 50.098 57.543 10.683

ATOM 664 4C66 GRA 1 52.397 61.861 11.207

ATOM 665 5C66 GRA 1 54.696 66.179 11.732

ATOM 666 6C66 GRA 1 56.995 70.498 12.256

ATOM 667 7C66 GRA 1 34.322 18.843 6.158

ATOM 668 8C66 GRA 1 36.621 23.161 6.683

ATOM 669 9C66 GRA 1 38.920 27.479 7.207

ATOM 670 0C67 GRA 1 41.218 31.798 7.732

ATOM 671 1C67 GRA 1 43.517 36.116 8.256

ATOM 672 2C67 GRA 1 45.816 40.434 8.781

ATOM 673 3C67 GRA 1 48.115 44.752 9.305

ATOM 674 4C67 GRA 1 50.414 49.070 9.830

ATOM 675 5C67 GRA 1 52.713 53.388 10.354

ATOM 676 6C67 GRA 1 55.012 57.706 10.879

ATOM 677 7C67 GRA 1 57.311 62.025 11.403

ATOM 678 8C67 GRA 1 59.610 66.343 11.928

ATOM 679 9C67 GRA 1 61.909 70.661 12.452

ATOM 680 0C68 GRA 1 39.234 19.007 6.355

ATOM 681 1C68 GRA 1 41.533 23.325 6.879

ATOM 682 2C68 GRA 1 43.832 27.643 7.404

ATOM 683 3C68 GRA 1 46.131 31.961 7.928

ATOM 684 4C68 GRA 1 48.430 36.279 8.453

ATOM 685 5C68 GRA 1 50.729 40.597 8.977

ATOM 686 6C68 GRA 1 53.028 44.916 9.502

ATOM 687 7C68 GRA 1 55.327 49.234 10.026

ATOM 688 8C68 GRA 1 57.626 53.552 10.551

ATOM 689 9C68 GRA 1 59.924 57.870 11.075

ATOM 690 0C69 GRA 1 62.223 62.188 11.600

ATOM 691 1C69 GRA 1 64.522 66.506 12.124

ATOM 692 2C69 GRA 1 66.821 70.824 12.649

ATOM 693 3C69 GRA 1 44.148 19.170 6.551

ATOM 694 4C69 GRA 1 46.447 23.488 7.075

ATOM 695 5C69 GRA 1 48.746 27.806 7.600

ATOM 696 6C69 GRA 1 51.045 32.124 8.124

ATOM 697 7C69 GRA 1 53.343 36.442 8.649

ATOM 698 8C69 GRA 1 55.642 40.761 9.173

ATOM 699 9C69 GRA 1 57.941 45.079 9.698

ATOM 700 0C70 GRA 1 60.240 49.397 10.223

ATOM 701 1C70 GRA 1 62.539 53.715 10.747

ATOM 702 2C70 GRA 1 64.838 58.033 11.272

ATOM 703 3C70 GRA 1 67.137 62.351 11.796

ATOM 704 4C70 GRA 1 69.436 66.669 12.321

ATOM 705 5C70 GRA 1 71.735 70.988 12.845

ATOM 706 6C70 GRA 1 49.061 19.333 6.747

ATOM 707 7C70 GRA 1 51.360 23.651 7.272

ATOM 708 8C70 GRA 1 53.659 27.969 7.796

ATOM 709 9C70 GRA 1 55.958 32.287 8.321

ATOM 710 0C71 GRA 1 58.257 36.606 8.845

ATOM 711 1C71 GRA 1 60.556 40.924 9.370

ATOM 712 2C71 GRA 1 62.855 45.242 9.894

ATOM 713 3C71 GRA 1 65.154 49.560 10.419

ATOM 714 4C71 GRA 1 67.453 53.878 10.943

ATOM 715 5C71 GRA 1 69.751 58.196 11.468

ATOM 716 6C71 GRA 1 72.050 62.514 11.992

ATOM 717 7C71 GRA 1 74.349 66.833 12.517

ATOM 718 8C71 GRA 1 76.648 71.151 13.041

ATOM 719 9C71 GRA 1 53.975 19.496 6.944

ATOM 720 0C72 GRA 1 56.274 23.814 7.468

ATOM 721 1C72 GRA 1 58.573 28.133 7.993

ATOM 722 2C72 GRA 1 60.872 32.451 8.517

ATOM 723 3C72 GRA 1 63.170 36.769 9.042

ATOM 724 4C72 GRA 1 65.469 41.087 9.566

ATOM 725 5C72 GRA 1 67.768 45.405 10.091

ATOM 726 6C72 GRA 1 70.067 49.723 10.615

ATOM 727 7C72 GRA 1 72.366 54.041 11.140

ATOM 728 8C72 GRA 1 74.665 58.359 11.664

ATOM 729 9C72 GRA 1 76.964 62.678 12.189

ATOM 730 0C73 GRA 1 79.263 66.996 12.713

ATOM 731 1C73 GRA 1 81.562 71.314 13.238

ATOM 732 2C73 GRA 1 58.888 19.659 7.140

ATOM 733 3C73 GRA 1 61.187 23.978 7.664

ATOM 734 4C73 GRA 1 63.486 28.296 8.189

ATOM 735 5C73 GRA 1 65.785 32.614 8.713

ATOM 736 6C73 GRA 1 68.084 36.932 9.238

ATOM 737 7C73 GRA 1 70.383 41.250 9.762

ATOM 738 8C73 GRA 1 72.682 45.568 10.287

ATOM 739 9C73 GRA 1 74.981 49.886 10.812

ATOM 740 0C74 GRA 1 77.280 54.205 11.336

ATOM 741 1C74 GRA 1 79.578 58.523 11.861

ATOM 742 2C74 GRA 1 81.877 62.841 12.385

ATOM 743 3C74 GRA 1 63.802 19.823 7.336

ATOM 744 4C74 GRA 1 66.101 24.141 7.861

ATOM 745 5C74 GRA 1 68.400 28.459 8.385

ATOM 746 6C74 GRA 1 70.699 32.777 8.910

ATOM 747 7C74 GRA 1 72.997 37.095 9.434

ATOM 748 8C74 GRA 1 75.296 41.413 9.959

ATOM 749 9C74 GRA 1 77.595 45.731 10.483

ATOM 750 0C75 GRA 1 79.894 50.050 11.008

ATOM 751 1C75 GRA 1 82.193 54.368 11.532

ATOM 752 2C75 GRA 1 68.714 19.986 7.533

ATOM 753 3C75 GRA 1 71.013 24.304 8.057

ATOM 754 4C75 GRA 1 73.312 28.622 8.582

ATOM 755 5C75 GRA 1 75.611 32.941 9.106

ATOM 756 6C75 GRA 1 77.910 37.259 9.631

ATOM 757 7C75 GRA 1 80.209 41.577 10.155

ATOM 758 8C75 GRA 1 82.508 45.895 10.680

ATOM 759 9C75 GRA 1 73.628 20.149 7.729

ATOM 760 0C76 GRA 1 75.927 24.467 8.253

ATOM 761 1C76 GRA 1 78.226 28.786 8.778

ATOM 762 2C76 GRA 1 80.525 33.104 9.302

ATOM 763 3C76 GRA 1 82.824 37.422 9.827

ATOM 764 4C76 GRA 1 78.542 20.313 7.925

ATOM 765 5C76 GRA 1 80.840 24.631 8.450

ATOM 766 6C76 GRA 1 83.139 28.949 8.974

ATOM 767 7C76 GRA 1 83.455 20.476 8.122

ATOM 768 8C76 GRA 1 10.370 67.535 10.249

ATOM 769 9C76 GRA 1 10.686 59.062 9.396

ATOM 770 0C77 GRA 1 12.985 63.380 9.921

ATOM 771 1C77 GRA 1 15.284 67.698 10.445

ATOM 772 2C77 GRA 1 11.002 50.589 8.543

ATOM 773 3C77 GRA 1 13.301 54.907 9.068

ATOM 774 4C77 GRA 1 15.600 59.225 9.592

ATOM 775 5C77 GRA 1 17.899 63.543 10.117

ATOM 776 6C77 GRA 1 20.197 67.861 10.641

ATOM 777 7C77 GRA 1 11.317 42.116 7.691

ATOM 778 8C77 GRA 1 13.616 46.434 8.215

ATOM 779 9C77 GRA 1 15.915 50.752 8.740

ATOM 780 0C78 GRA 1 18.213 55.070 9.264

ATOM 781 1C78 GRA 1 20.512 59.388 9.789

ATOM 782 2C78 GRA 1 22.811 63.707 10.313

ATOM 783 3C78 GRA 1 25.110 68.025 10.838

ATOM 784 4C78 GRA 1 11.632 33.643 6.838

ATOM 785 5C78 GRA 1 13.931 37.961 7.363

ATOM 786 6C78 GRA 1 16.230 42.279 7.887

ATOM 787 7C78 GRA 1 18.529 46.597 8.412

ATOM 788 8C78 GRA 1 20.828 50.915 8.936

ATOM 789 9C78 GRA 1 23.127 55.233 9.461

ATOM 790 0C79 GRA 1 25.426 59.552 9.985

ATOM 791 1C79 GRA 1 27.725 63.870 10.510

ATOM 792 2C79 GRA 1 30.024 68.188 11.034

ATOM 793 3C79 GRA 1 11.948 25.170 5.985

ATOM 794 4C79 GRA 1 14.247 29.488 6.510

ATOM 795 5C79 GRA 1 16.546 33.806 7.034

ATOM 796 6C79 GRA 1 18.845 38.124 7.559

ATOM 797 7C79 GRA 1 21.144 42.442 8.083

ATOM 798 8C79 GRA 1 23.443 46.760 8.608

ATOM 799 9C79 GRA 1 25.742 51.078 9.132

ATOM 800 0C80 GRA 1 28.040 55.397 9.657

ATOM 801 1C80 GRA 1 30.339 59.715 10.181

ATOM 802 2C80 GRA 1 32.638 64.033 10.706

ATOM 803 3C80 GRA 1 34.937 68.351 11.230

ATOM 804 4C80 GRA 1 12.264 16.697 5.133

ATOM 805 5C80 GRA 1 14.563 21.015 5.657

ATOM 806 6C80 GRA 1 16.862 25.333 6.182

ATOM 807 7C80 GRA 1 19.161 29.651 6.706

ATOM 808 8C80 GRA 1 21.459 33.969 7.231

ATOM 809 9C80 GRA 1 23.758 38.287 7.755

ATOM 810 0C81 GRA 1 26.057 42.605 8.280

ATOM 811 1C81 GRA 1 28.356 46.924 8.804

ATOM 812 2C81 GRA 1 30.655 51.242 9.329

ATOM 813 3C81 GRA 1 32.954 55.560 9.853

ATOM 814 4C81 GRA 1 35.253 59.878 10.378

ATOM 815 5C81 GRA 1 37.552 64.196 10.902

ATOM 816 6C81 GRA 1 39.851 68.514 11.427

ATOM 817 7C81 GRA 1 17.177 16.860 5.329

ATOM 818 8C81 GRA 1 19.476 21.178 5.853

ATOM 819 9C81 GRA 1 21.775 25.496 6.378

ATOM 820 0C82 GRA 1 24.074 29.814 6.902

ATOM 821 1C82 GRA 1 26.373 34.132 7.427

ATOM 822 2C82 GRA 1 28.672 38.450 7.952

ATOM 823 3C82 GRA 1 30.971 42.769 8.476

ATOM 824 4C82 GRA 1 33.270 47.087 9.001

ATOM 825 5C82 GRA 1 35.569 51.405 9.525

ATOM 826 6C82 GRA 1 37.867 55.723 10.050

ATOM 827 7C82 GRA 1 40.166 60.041 10.574

ATOM 828 8C82 GRA 1 42.465 64.359 11.099

ATOM 829 9C82 GRA 1 44.764 68.677 11.623

ATOM 830 0C83 GRA 1 22.091 17.023 5.525

ATOM 831 1C83 GRA 1 24.390 21.341 6.050

ATOM 832 2C83 GRA 1 26.689 25.659 6.574

ATOM 833 3C83 GRA 1 28.988 29.977 7.099

ATOM 834 4C83 GRA 1 31.286 34.295 7.623

ATOM 835 5C83 GRA 1 33.585 38.614 8.148

ATOM 836 6C83 GRA 1 35.884 42.932 8.672

ATOM 837 7C83 GRA 1 38.183 47.250 9.197

ATOM 838 8C83 GRA 1 40.482 51.568 9.721

ATOM 839 9C83 GRA 1 42.781 55.886 10.246

ATOM 840 0C84 GRA 1 45.080 60.204 10.770

ATOM 841 1C84 GRA 1 47.379 64.522 11.295

ATOM 842 2C84 GRA 1 49.678 68.841 11.819

ATOM 843 3C84 GRA 1 27.003 17.186 5.722

ATOM 844 4C84 GRA 1 29.302 21.505 6.246

ATOM 845 5C84 GRA 1 31.601 25.823 6.771

ATOM 846 6C84 GRA 1 33.900 30.141 7.295

ATOM 847 7C84 GRA 1 36.199 34.459 7.820

ATOM 848 8C84 GRA 1 38.498 38.777 8.344

ATOM 849 9C84 GRA 1 40.797 43.095 8.869

ATOM 850 0C85 GRA 1 43.096 47.413 9.393

ATOM 851 1C85 GRA 1 45.395 51.732 9.918

ATOM 852 2C85 GRA 1 47.694 56.050 10.442

ATOM 853 3C85 GRA 1 49.992 60.368 10.967

ATOM 854 4C85 GRA 1 52.291 64.686 11.491

ATOM 855 5C85 GRA 1 54.590 69.004 12.016

ATOM 856 6C85 GRA 1 31.917 17.350 5.918

ATOM 857 7C85 GRA 1 34.216 21.668 6.442

ATOM 858 8C85 GRA 1 36.515 25.986 6.967

ATOM 859 9C85 GRA 1 38.814 30.304 7.492

ATOM 860 0C86 GRA 1 41.113 34.622 8.016

ATOM 861 1C86 GRA 1 43.411 38.940 8.541

ATOM 862 2C86 GRA 1 45.710 43.258 9.065

ATOM 863 3C86 GRA 1 48.009 47.577 9.590

ATOM 864 4C86 GRA 1 50.308 51.895 10.114

ATOM 865 5C86 GRA 1 52.607 56.213 10.639

ATOM 866 6C86 GRA 1 54.906 60.531 11.163

ATOM 867 7C86 GRA 1 57.205 64.849 11.688

ATOM 868 8C86 GRA 1 59.504 69.167 12.212

ATOM 869 9C86 GRA 1 36.830 17.513 6.114

ATOM 870 0C87 GRA 1 39.129 21.831 6.639

ATOM 871 1C87 GRA 1 41.428 26.149 7.163

ATOM 872 2C87 GRA 1 43.727 30.467 7.688

ATOM 873 3C87 GRA 1 46.026 34.785 8.212

ATOM 874 4C87 GRA 1 48.325 39.104 8.737

ATOM 875 5C87 GRA 1 50.624 43.422 9.261

ATOM 876 6C87 GRA 1 52.923 47.740 9.786

ATOM 877 7C87 GRA 1 55.222 52.058 10.310

ATOM 878 8C87 GRA 1 57.521 56.376 10.835

ATOM 879 9C87 GRA 1 59.819 60.694 11.359

ATOM 880 0C88 GRA 1 62.118 65.012 11.884

ATOM 881 1C88 GRA 1 64.417 69.331 12.408

ATOM 882 2C88 GRA 1 41.744 17.676 6.311

ATOM 883 3C88 GRA 1 44.043 21.994 6.835

ATOM 884 4C88 GRA 1 46.342 26.312 7.360

ATOM 885 5C88 GRA 1 48.641 30.630 7.884

ATOM 886 6C88 GRA 1 50.940 34.949 8.409

ATOM 887 7C88 GRA 1 53.239 39.267 8.933

ATOM 888 8C88 GRA 1 55.537 43.585 9.458

ATOM 889 9C88 GRA 1 57.836 47.903 9.982

ATOM 890 0C89 GRA 1 60.135 52.221 10.507

ATOM 891 1C89 GRA 1 62.434 56.539 11.031

ATOM 892 2C89 GRA 1 64.733 60.857 11.556

ATOM 893 3C89 GRA 1 67.032 65.176 12.080

ATOM 894 4C89 GRA 1 69.331 69.494 12.605

ATOM 895 5C89 GRA 1 46.658 17.839 6.507

ATOM 896 6C89 GRA 1 48.956 22.157 7.031

ATOM 897 7C89 GRA 1 51.255 26.475 7.556

ATOM 898 8C89 GRA 1 53.554 30.794 8.081

ATOM 899 9C89 GRA 1 55.853 35.112 8.605

ATOM 900 0C90 GRA 1 58.152 39.430 9.130

ATOM 901 1C90 GRA 1 60.451 43.748 9.654

ATOM 902 2C90 GRA 1 62.750 48.066 10.179

ATOM 903 3C90 GRA 1 65.049 52.384 10.703

ATOM 904 4C90 GRA 1 67.348 56.702 11.228

ATOM 905 5C90 GRA 1 69.647 61.021 11.752

ATOM 906 6C90 GRA 1 71.945 65.339 12.277

ATOM 907 7C90 GRA 1 74.244 69.657 12.801

ATOM 908 8C90 GRA 1 51.571 18.002 6.703

ATOM 909 9C90 GRA 1 53.870 22.320 7.228

ATOM 910 0C91 GRA 1 56.169 26.639 7.752

ATOM 911 1C91 GRA 1 58.468 30.957 8.277

ATOM 912 2C91 GRA 1 60.767 35.275 8.801

ATOM 913 3C91 GRA 1 63.066 39.593 9.326

ATOM 914 4C91 GRA 1 65.364 43.911 9.850

ATOM 915 5C91 GRA 1 67.663 48.229 10.375

ATOM 916 6C91 GRA 1 69.962 52.547 10.899

ATOM 917 7C91 GRA 1 72.261 56.866 11.424

ATOM 918 8C91 GRA 1 74.560 61.184 11.948

ATOM 919 9C91 GRA 1 76.859 65.502 12.473

ATOM 920 0C92 GRA 1 79.158 69.820 12.997

ATOM 921 1C92 GRA 1 56.485 18.165 6.900

ATOM 922 2C92 GRA 1 58.783 22.484 7.424

ATOM 923 3C92 GRA 1 61.082 26.802 7.949

ATOM 924 4C92 GRA 1 63.381 31.120 8.473

ATOM 925 5C92 GRA 1 65.680 35.438 8.998

ATOM 926 6C92 GRA 1 67.979 39.756 9.522

ATOM 927 7C92 GRA 1 70.278 44.074 10.047

ATOM 928 8C92 GRA 1 72.577 48.392 10.571

ATOM 929 9C92 GRA 1 74.876 52.711 11.096

ATOM 930 0C93 GRA 1 77.175 57.029 11.620

ATOM 931 1C93 GRA 1 79.474 61.347 12.145

ATOM 932 2C93 GRA 1 81.772 65.665 12.669

ATOM 933 3C93 GRA 1 61.397 18.329 7.096

ATOM 934 4C93 GRA 1 63.696 22.647 7.621

ATOM 935 5C93 GRA 1 65.995 26.965 8.145

ATOM 936 6C93 GRA 1 68.294 31.284 8.670

ATOM 937 7C93 GRA 1 70.593 35.602 9.194

ATOM 938 8C93 GRA 1 72.892 39.920 9.719

ATOM 939 9C93 GRA 1 75.191 44.238 10.243

ATOM 940 0C94 GRA 1 77.489 48.556 10.768

ATOM 941 1C94 GRA 1 79.788 52.874 11.292

ATOM 942 2C94 GRA 1 82.087 57.192 11.817

ATOM 943 3C94 GRA 1 66.311 18.492 7.292

ATOM 944 4C94 GRA 1 68.610 22.810 7.817

ATOM 945 5C94 GRA 1 70.908 27.129 8.341

ATOM 946 6C94 GRA 1 73.207 31.447 8.866

ATOM 947 7C94 GRA 1 75.506 35.765 9.390

ATOM 948 8C94 GRA 1 77.805 40.083 9.915

ATOM 949 9C94 GRA 1 80.104 44.401 10.439

ATOM 950 0C95 GRA 1 82.403 48.719 10.964

ATOM 951 1C95 GRA 1 71.224 18.655 7.489

ATOM 952 2C95 GRA 1 73.523 22.974 8.013

ATOM 953 3C95 GRA 1 75.822 27.292 8.538

ATOM 954 4C95 GRA 1 78.121 31.610 9.062

ATOM 955 5C95 GRA 1 80.420 35.928 9.587

ATOM 956 6C95 GRA 1 82.719 40.246 10.111

ATOM 957 7C95 GRA 1 76.138 18.819 7.685

ATOM 958 8C95 GRA 1 78.437 23.137 8.210

ATOM 959 9C95 GRA 1 80.735 27.455 8.734

ATOM 960 0C96 GRA 1 83.034 31.773 9.259

ATOM 961 1C96 GRA 1 81.051 18.982 7.881

ATOM 962 2C96 GRA 1 83.350 23.300 8.406

ATOM 963 3C96 GRA 1 11.520 69.694 10.511

ATOM 964 4C96 GRA 1 11.836 61.221 9.658

ATOM 965 5C96 GRA 1 14.135 65.539 10.183

ATOM 966 6C96 GRA 1 16.433 69.857 10.707

ATOM 967 7C96 GRA 1 12.151 52.748 8.806

ATOM 968 8C96 GRA 1 14.450 57.066 9.330

ATOM 969 9C96 GRA 1 16.749 61.384 9.855

ATOM 970 0C97 GRA 1 19.048 65.702 10.379

ATOM 971 1C97 GRA 1 21.347 70.020 10.904

ATOM 972 2C97 GRA 1 12.466 44.275 7.953

ATOM 973 3C97 GRA 1 14.765 48.593 8.477

ATOM 974 4C97 GRA 1 17.064 52.911 9.002

ATOM 975 5C97 GRA 1 19.363 57.229 9.526

ATOM 976 6C97 GRA 1 21.662 61.548 10.051

ATOM 977 7C97 GRA 1 23.961 65.866 10.575

ATOM 978 8C97 GRA 1 26.260 70.184 11.100

ATOM 979 9C97 GRA 1 12.782 35.802 7.100

ATOM 980 0C98 GRA 1 15.081 40.120 7.625

ATOM 981 1C98 GRA 1 17.380 44.438 8.149

ATOM 982 2C98 GRA 1 19.679 48.756 8.674

ATOM 983 3C98 GRA 1 21.977 53.074 9.198

ATOM 984 4C98 GRA 1 24.276 57.393 9.723

ATOM 985 5C98 GRA 1 26.575 61.711 10.247

ATOM 986 6C98 GRA 1 28.874 66.029 10.772

ATOM 987 7C98 GRA 1 31.173 70.347 11.296

ATOM 988 8C98 GRA 1 13.098 27.329 6.248

ATOM 989 9C98 GRA 1 15.396 31.647 6.772

ATOM 990 0C99 GRA 1 17.695 35.965 7.297

ATOM 991 1C99 GRA 1 19.994 40.283 7.821

ATOM 992 2C99 GRA 1 22.293 44.601 8.346

ATOM 993 3C99 GRA 1 24.592 48.919 8.870

ATOM 994 4C99 GRA 1 26.891 53.238 9.395

ATOM 995 5C99 GRA 1 29.190 57.556 9.919

ATOM 996 6C99 GRA 1 31.489 61.874 10.444

ATOM 997 7C99 GRA 1 33.788 66.192 10.968

ATOM 998 8C99 GRA 1 36.087 70.510 11.493

ATOM 999 9C99 GRA 1 13.413 18.856 5.395

ATOM 1000 0C00 GRA 1 15.712 23.174 5.919

ATOM 1001 1C00 GRA 1 18.011 27.492 6.444

ATOM 1002 2C00 GRA 1 20.310 31.810 6.968

ATOM 1003 3C00 GRA 1 22.609 36.128 7.493

ATOM 1004 4C00 GRA 1 24.908 40.446 8.017

ATOM 1005 5C00 GRA 1 27.207 44.764 8.542

ATOM 1006 6C00 GRA 1 29.506 49.083 9.066

ATOM 1007 7C00 GRA 1 31.804 53.401 9.591

ATOM 1008 8C00 GRA 1 34.103 57.719 10.115

ATOM 1009 9C00 GRA 1 36.402 62.037 10.640

ATOM 1010 0C01 GRA 1 38.701 66.355 11.165

ATOM 1011 1C01 GRA 1 41.000 70.673 11.689

ATOM 1012 2C01 GRA 1 18.327 19.019 5.591

ATOM 1013 3C01 GRA 1 20.626 23.337 6.116

ATOM 1014 4C01 GRA 1 22.925 27.655 6.640

ATOM 1015 5C01 GRA 1 25.223 31.973 7.165

ATOM 1016 6C01 GRA 1 27.522 36.291 7.689

ATOM 1017 7C01 GRA 1 29.821 40.609 8.214

ATOM 1018 8C01 GRA 1 32.120 44.928 8.738

ATOM 1019 9C01 GRA 1 34.419 49.246 9.263

ATOM 1020 0C02 GRA 1 36.718 53.564 9.787

ATOM 1021 1C02 GRA 1 39.017 57.882 10.312

ATOM 1022 2C02 GRA 1 41.316 62.200 10.836

ATOM 1023 3C02 GRA 1 43.615 66.518 11.361

ATOM 1024 4C02 GRA 1 45.914 70.836 11.885

ATOM 1025 5C02 GRA 1 23.240 19.182 5.788

ATOM 1026 6C02 GRA 1 25.539 23.500 6.312

ATOM 1027 7C02 GRA 1 27.838 27.818 6.837

ATOM 1028 8C02 GRA 1 30.137 32.136 7.361

ATOM 1029 9C02 GRA 1 32.436 36.454 7.886

ATOM 1030 0C03 GRA 1 34.735 40.773 8.410

ATOM 1031 1C03 GRA 1 37.034 45.091 8.935

ATOM 1032 2C03 GRA 1 39.333 49.409 9.459

ATOM 1033 3C03 GRA 1 41.632 53.727 9.984

ATOM 1034 4C03 GRA 1 43.930 58.045 10.508

ATOM 1035 5C03 GRA 1 46.229 62.363 11.033

ATOM 1036 6C03 GRA 1 48.528 66.681 11.557

ATOM 1037 7C03 GRA 1 50.827 71.000 12.082

ATOM 1038 8C03 GRA 1 28.153 19.346 5.984

ATOM 1039 9C03 GRA 1 30.452 23.664 6.508

ATOM 1040 0C04 GRA 1 32.751 27.982 7.033

ATOM 1041 1C04 GRA 1 35.050 32.300 7.557

ATOM 1042 2C04 GRA 1 37.349 36.618 8.082

ATOM 1043 3C04 GRA 1 39.647 40.936 8.606

ATOM 1044 4C04 GRA 1 41.946 45.254 9.131

ATOM 1045 5C04 GRA 1 44.245 49.573 9.655

ATOM 1046 6C04 GRA 1 46.544 53.891 10.180

ATOM 1047 7C04 GRA 1 48.843 58.209 10.704

ATOM 1048 8C04 GRA 1 51.142 62.527 11.229

ATOM 1049 9C04 GRA 1 53.441 66.845 11.754

ATOM 1050 0C05 GRA 1 55.740 71.163 12.278

ATOM 1051 1C05 GRA 1 33.066 19.509 6.180

ATOM 1052 2C05 GRA 1 35.365 23.827 6.705

ATOM 1053 3C05 GRA 1 37.664 28.145 7.229

ATOM 1054 4C05 GRA 1 39.963 32.463 7.754

ATOM 1055 5C05 GRA 1 42.262 36.781 8.278

ATOM 1056 6C05 GRA 1 44.561 41.099 8.803

ATOM 1057 7C05 GRA 1 46.860 45.418 9.327

ATOM 1058 8C05 GRA 1 49.159 49.736 9.852

ATOM 1059 9C05 GRA 1 51.458 54.054 10.376

ATOM 1060 0C06 GRA 1 53.757 58.372 10.901

ATOM 1061 1C06 GRA 1 56.055 62.690 11.425

ATOM 1062 2C06 GRA 1 58.354 67.008 11.950

ATOM 1063 3C06 GRA 1 60.653 71.326 12.474

ATOM 1064 4C06 GRA 1 37.980 19.672 6.377

ATOM 1065 5C06 GRA 1 40.279 23.990 6.901

ATOM 1066 6C06 GRA 1 42.578 28.308 7.426

ATOM 1067 7C06 GRA 1 44.877 32.626 7.950

ATOM 1068 8C06 GRA 1 47.176 36.944 8.475

ATOM 1069 9C06 GRA 1 49.474 41.263 8.999

ATOM 1070 0C07 GRA 1 51.773 45.581 9.524

ATOM 1071 1C07 GRA 1 54.072 49.899 10.048

ATOM 1072 2C07 GRA 1 56.371 54.217 10.573

ATOM 1073 3C07 GRA 1 58.670 58.535 11.097

ATOM 1074 4C07 GRA 1 60.969 62.853 11.622

ATOM 1075 5C07 GRA 1 63.268 67.171 12.146

ATOM 1076 6C07 GRA 1 65.567 71.490 12.671

ATOM 1077 7C07 GRA 1 42.893 19.835 6.573

ATOM 1078 8C07 GRA 1 45.192 24.153 7.097

ATOM 1079 9C07 GRA 1 47.491 28.471 7.622

ATOM 1080 0C08 GRA 1 49.790 32.789 8.146

ATOM 1081 1C08 GRA 1 52.089 37.108 8.671

ATOM 1082 2C08 GRA 1 54.388 41.426 9.195

ATOM 1083 3C08 GRA 1 56.687 45.744 9.720

ATOM 1084 4C08 GRA 1 58.986 50.062 10.244

ATOM 1085 5C08 GRA 1 61.285 54.380 10.769

ATOM 1086 6C08 GRA 1 63.584 58.698 11.294

ATOM 1087 7C08 GRA 1 65.882 63.016 11.818

ATOM 1088 8C08 GRA 1 68.181 67.335 12.343

ATOM 1089 9C08 GRA 1 70.480 71.653 12.867

ATOM 1090 0C09 GRA 1 47.807 19.998 6.769

ATOM 1091 1C09 GRA 1 50.106 24.316 7.294

ATOM 1092 2C09 GRA 1 52.405 28.634 7.818

ATOM 1093 3C09 GRA 1 54.704 32.953 8.343

ATOM 1094 4C09 GRA 1 57.003 37.271 8.867

ATOM 1095 5C09 GRA 1 59.301 41.589 9.392

ATOM 1096 6C09 GRA 1 61.600 45.907 9.916

ATOM 1097 7C09 GRA 1 63.899 50.225 10.441

ATOM 1098 8C09 GRA 1 66.198 54.543 10.965

ATOM 1099 9C09 GRA 1 68.497 58.861 11.490

ATOM 1100 01C0 GRA 1 70.796 63.180 12.014

ATOM 1101 11C0 GRA 1 73.095 67.498 12.539

ATOM 1102 21C0 GRA 1 75.394 71.816 13.063

ATOM 1103 31C0 GRA 1 52.720 20.161 6.966

ATOM 1104 41C0 GRA 1 55.019 24.480 7.490

ATOM 1105 51C0 GRA 1 57.318 28.798 8.015

ATOM 1106 61C0 GRA 1 59.617 33.116 8.539

ATOM 1107 71C0 GRA 1 61.916 37.434 9.064

ATOM 1108 81C0 GRA 1 64.215 41.752 9.588

ATOM 1109 91C0 GRA 1 66.514 46.070 10.113

ATOM 1110 01C1 GRA 1 68.813 50.388 10.637

ATOM 1111 11C1 GRA 1 71.112 54.707 11.162

ATOM 1112 21C1 GRA 1 73.411 59.025 11.686

ATOM 1113 31C1 GRA 1 75.709 63.343 12.211

ATOM 1114 41C1 GRA 1 78.008 67.661 12.735

ATOM 1115 51C1 GRA 1 80.307 71.979 13.260

ATOM 1116 61C1 GRA 1 57.634 20.325 7.162

ATOM 1117 71C1 GRA 1 59.933 24.643 7.686

ATOM 1118 81C1 GRA 1 62.232 28.961 8.211

ATOM 1119 91C1 GRA 1 64.531 33.279 8.735

ATOM 1120 01C2 GRA 1 66.830 37.597 9.260

ATOM 1121 11C2 GRA 1 69.128 41.915 9.784

ATOM 1122 21C2 GRA 1 71.427 46.233 10.309

ATOM 1123 31C2 GRA 1 73.726 50.552 10.833

ATOM 1124 41C2 GRA 1 76.025 54.870 11.358

ATOM 1125 51C2 GRA 1 78.324 59.188 11.883

ATOM 1126 61C2 GRA 1 80.623 63.506 12.407

ATOM 1127 71C2 GRA 1 62.547 20.488 7.358

ATOM 1128 81C2 GRA 1 64.846 24.806 7.883

ATOM 1129 91C2 GRA 1 67.144 29.124 8.407

ATOM 1130 01C3 GRA 1 69.443 33.443 8.932

ATOM 1131 11C3 GRA 1 71.742 37.761 9.456

ATOM 1132 21C3 GRA 1 74.041 42.079 9.981

ATOM 1133 31C3 GRA 1 76.340 46.397 10.505

ATOM 1134 41C3 GRA 1 78.639 50.715 11.030

ATOM 1135 51C3 GRA 1 80.938 55.033 11.554

ATOM 1136 61C3 GRA 1 67.460 20.651 7.555

ATOM 1137 71C3 GRA 1 69.759 24.969 8.079

ATOM 1138 81C3 GRA 1 72.058 29.288 8.604

ATOM 1139 91C3 GRA 1 74.357 33.606 9.128

ATOM 1140 01C4 GRA 1 76.656 37.924 9.653

ATOM 1141 11C4 GRA 1 78.955 42.242 10.177

ATOM 1142 21C4 GRA 1 81.253 46.560 10.702

ATOM 1143 31C4 GRA 1 72.374 20.814 7.751

ATOM 1144 41C4 GRA 1 74.673 25.133 8.275

ATOM 1145 51C4 GRA 1 76.971 29.451 8.800

ATOM 1146 61C4 GRA 1 79.270 33.769 9.324

ATOM 1147 71C4 GRA 1 81.569 38.087 9.849

ATOM 1148 81C4 GRA 1 77.287 20.978 7.947

ATOM 1149 91C4 GRA 1 79.586 25.296 8.472

ATOM 1150 01C5 GRA 1 81.885 29.614 8.996

ATOM 1151 11C5 GRA 1 82.201 21.141 8.144

ATOM 1152 21C5 GRA 1 11.677 65.458 10.085

ATOM 1153 31C5 GRA 1 13.976 69.776 10.609

ATOM 1154 41C5 GRA 1 11.993 56.984 9.232

ATOM 1155 51C5 GRA 1 14.292 61.303 9.756

ATOM 1156 61C5 GRA 1 16.591 65.621 10.281

ATOM 1157 71C5 GRA 1 18.890 69.939 10.805

ATOM 1158 81C5 GRA 1 12.309 48.511 8.379

ATOM 1159 91C5 GRA 1 14.608 52.829 8.904

ATOM 1160 01C6 GRA 1 16.907 57.148 9.428

ATOM 1161 11C6 GRA 1 19.205 61.466 9.953

ATOM 1162 21C6 GRA 1 21.504 65.784 10.477

ATOM 1163 31C6 GRA 1 23.803 70.102 11.002

ATOM 1164 41C6 GRA 1 12.624 40.038 7.527

ATOM 1165 51C6 GRA 1 14.923 44.356 8.051

ATOM 1166 61C6 GRA 1 17.222 48.674 8.576

ATOM 1167 71C6 GRA 1 19.521 52.993 9.100

ATOM 1168 81C6 GRA 1 21.820 57.311 9.625

ATOM 1169 91C6 GRA 1 24.119 61.629 10.149

ATOM 1170 01C7 GRA 1 26.418 65.947 10.674

ATOM 1171 11C7 GRA 1 28.717 70.265 11.198

ATOM 1172 21C7 GRA 1 12.940 31.565 6.674

ATOM 1173 31C7 GRA 1 15.239 35.883 7.198

ATOM 1174 41C7 GRA 1 17.538 40.201 7.723

ATOM 1175 51C7 GRA 1 19.837 44.519 8.247

ATOM 1176 61C7 GRA 1 22.136 48.838 8.772

ATOM 1177 71C7 GRA 1 24.435 53.156 9.296

ATOM 1178 81C7 GRA 1 26.734 57.474 9.821

ATOM 1179 91C7 GRA 1 29.032 61.792 10.345

ATOM 1180 01C8 GRA 1 31.331 66.110 10.870

ATOM 1181 11C8 GRA 1 33.630 70.428 11.395

ATOM 1182 21C8 GRA 1 13.256 23.092 5.821

ATOM 1183 31C8 GRA 1 15.555 27.410 6.346

ATOM 1184 41C8 GRA 1 17.854 31.728 6.870

ATOM 1185 51C8 GRA 1 20.153 36.046 7.395

ATOM 1186 61C8 GRA 1 22.451 40.364 7.919

ATOM 1187 71C8 GRA 1 24.750 44.683 8.444

ATOM 1188 81C8 GRA 1 27.049 49.001 8.968

ATOM 1189 91C8 GRA 1 29.348 53.319 9.493

ATOM 1190 01C9 GRA 1 31.647 57.637 10.017

ATOM 1191 11C9 GRA 1 33.946 61.955 10.542

ATOM 1192 21C9 GRA 1 36.245 66.273 11.066

ATOM 1193 31C9 GRA 1 38.544 70.591 11.591

ATOM 1194 41C9 GRA 1 15.870 18.937 5.493

ATOM 1195 51C9 GRA 1 18.169 23.256 6.018

ATOM 1196 61C9 GRA 1 20.467 27.574 6.542

ATOM 1197 71C9 GRA 1 22.766 31.892 7.067

ATOM 1198 81C9 GRA 1 25.065 36.210 7.591

ATOM 1199 91C9 GRA 1 27.364 40.528 8.116

ATOM 1200 02C0 GRA 1 29.663 44.846 8.640

ATOM 1201 12C0 GRA 1 31.962 49.164 9.165

ATOM 1202 22C0 GRA 1 34.261 53.483 9.689

ATOM 1203 32C0 GRA 1 36.560 57.801 10.214

ATOM 1204 42C0 GRA 1 38.859 62.119 10.738

ATOM 1205 52C0 GRA 1 41.157 66.437 11.263

ATOM 1206 62C0 GRA 1 43.456 70.755 11.787

ATOM 1207 72C0 GRA 1 20.783 19.101 5.689

ATOM 1208 82C0 GRA 1 23.082 23.419 6.214

ATOM 1209 92C0 GRA 1 25.381 27.737 6.738

ATOM 1210 02C1 GRA 1 27.680 32.055 7.263

ATOM 1211 12C1 GRA 1 29.979 36.373 7.787

ATOM 1212 22C1 GRA 1 32.278 40.691 8.312

ATOM 1213 32C1 GRA 1 34.577 45.009 8.836

ATOM 1214 42C1 GRA 1 36.875 49.328 9.361

ATOM 1215 52C1 GRA 1 39.174 53.646 9.885

ATOM 1216 62C1 GRA 1 41.473 57.964 10.410

ATOM 1217 72C1 GRA 1 43.772 62.282 10.935

ATOM 1218 82C1 GRA 1 46.071 66.600 11.459

ATOM 1219 92C1 GRA 1 48.370 70.918 11.984

ATOM 1220 02C2 GRA 1 25.697 19.264 5.886

ATOM 1221 12C2 GRA 1 27.996 23.582 6.410

ATOM 1222 22C2 GRA 1 30.294 27.900 6.935

ATOM 1223 32C2 GRA 1 32.593 32.218 7.459

ATOM 1224 42C2 GRA 1 34.892 36.536 7.984

ATOM 1225 52C2 GRA 1 37.191 40.854 8.508

ATOM 1226 62C2 GRA 1 39.490 45.173 9.033

ATOM 1227 72C2 GRA 1 41.789 49.491 9.557

ATOM 1228 82C2 GRA 1 44.088 53.809 10.082

ATOM 1229 92C2 GRA 1 46.387 58.127 10.606

ATOM 1230 02C3 GRA 1 48.686 62.445 11.131

ATOM 1231 12C3 GRA 1 50.985 66.763 11.655

ATOM 1232 22C3 GRA 1 53.283 71.081 12.180

ATOM 1233 32C3 GRA 1 30.610 19.427 6.082

ATOM 1234 42C3 GRA 1 32.909 23.745 6.607

ATOM 1235 52C3 GRA 1 35.208 28.063 7.131

ATOM 1236 62C3 GRA 1 37.507 32.381 7.656

ATOM 1237 72C3 GRA 1 39.806 36.699 8.180

ATOM 1238 82C3 GRA 1 42.105 41.018 8.705

ATOM 1239 92C3 GRA 1 44.404 45.336 9.229

ATOM 1240 02C4 GRA 1 46.702 49.654 9.754

ATOM 1241 12C4 GRA 1 49.001 53.972 10.278

ATOM 1242 22C4 GRA 1 51.300 58.290 10.803

ATOM 1243 32C4 GRA 1 53.599 62.608 11.327

ATOM 1244 42C4 GRA 1 55.898 66.926 11.852

ATOM 1245 52C4 GRA 1 58.197 71.245 12.376

ATOM 1246 62C4 GRA 1 35.524 19.590 6.278

ATOM 1247 72C4 GRA 1 37.823 23.908 6.803

ATOM 1248 82C4 GRA 1 40.121 28.226 7.327

ATOM 1249 92C4 GRA 1 42.420 32.544 7.852

ATOM 1250 02C5 GRA 1 44.719 36.863 8.376

ATOM 1251 12C5 GRA 1 47.018 41.181 8.901

ATOM 1252 22C5 GRA 1 49.317 45.499 9.425

ATOM 1253 32C5 GRA 1 51.616 49.817 9.950

ATOM 1254 42C5 GRA 1 53.915 54.135 10.474

ATOM 1255 52C5 GRA 1 56.214 58.453 10.999

ATOM 1256 62C5 GRA 1 58.513 62.771 11.524

ATOM 1257 72C5 GRA 1 60.812 67.090 12.048

ATOM 1258 82C5 GRA 1 63.110 71.408 12.573

ATOM 1259 92C5 GRA 1 40.437 19.753 6.475

ATOM 1260 02C6 GRA 1 42.736 24.071 6.999

ATOM 1261 12C6 GRA 1 45.035 28.390 7.524

ATOM 1262 22C6 GRA 1 47.334 32.708 8.048

ATOM 1263 32C6 GRA 1 49.633 37.026 8.573

ATOM 1264 42C6 GRA 1 51.932 41.344 9.097

ATOM 1265 52C6 GRA 1 54.231 45.662 9.622

ATOM 1266 62C6 GRA 1 56.529 49.980 10.146

ATOM 1267 72C6 GRA 1 58.828 54.298 10.671

ATOM 1268 82C6 GRA 1 61.127 58.617 11.195

ATOM 1269 92C6 GRA 1 63.426 62.935 11.720

ATOM 1270 02C7 GRA 1 65.725 67.253 12.244

ATOM 1271 12C7 GRA 1 68.024 71.571 12.769

ATOM 1272 22C7 GRA 1 45.350 19.917 6.671

ATOM 1273 32C7 GRA 1 47.649 24.235 7.196

ATOM 1274 42C7 GRA 1 49.948 28.553 7.720

ATOM 1275 52C7 GRA 1 52.246 32.871 8.245

ATOM 1276 62C7 GRA 1 54.545 37.189 8.769

ATOM 1277 72C7 GRA 1 56.844 41.508 9.294

ATOM 1278 82C7 GRA 1 59.143 45.826 9.818

ATOM 1279 92C7 GRA 1 61.442 50.144 10.343

ATOM 1280 02C8 GRA 1 63.741 54.462 10.867

ATOM 1281 12C8 GRA 1 66.040 58.780 11.392

ATOM 1282 22C8 GRA 1 68.339 63.098 11.916

ATOM 1283 32C8 GRA 1 70.638 67.416 12.441

ATOM 1284 42C8 GRA 1 72.937 71.735 12.965

ATOM 1285 52C8 GRA 1 50.263 20.080 6.867

ATOM 1286 62C8 GRA 1 52.562 24.398 7.392

ATOM 1287 72C8 GRA 1 54.861 28.716 7.916

ATOM 1288 82C8 GRA 1 57.160 33.034 8.441

ATOM 1289 92C8 GRA 1 59.459 37.353 8.965

ATOM 1290 02C9 GRA 1 61.758 41.671 9.490

ATOM 1291 12C9 GRA 1 64.057 45.989 10.014

ATOM 1292 22C9 GRA 1 66.356 50.307 10.539

ATOM 1293 32C9 GRA 1 68.654 54.625 11.064

ATOM 1294 42C9 GRA 1 70.953 58.943 11.588

ATOM 1295 52C9 GRA 1 73.252 63.261 12.113

ATOM 1296 62C9 GRA 1 75.551 67.580 12.637

ATOM 1297 72C9 GRA 1 77.850 71.898 13.162

ATOM 1298 82C9 GRA 1 55.177 20.243 7.064

ATOM 1299 92C9 GRA 1 57.476 24.561 7.588

ATOM 1300 03C0 GRA 1 59.775 28.879 8.113

ATOM 1301 13C0 GRA 1 62.073 33.198 8.637

ATOM 1302 23C0 GRA 1 64.372 37.516 9.162

ATOM 1303 33C0 GRA 1 66.671 41.834 9.686

ATOM 1304 43C0 GRA 1 68.970 46.152 10.211

ATOM 1305 53C0 GRA 1 71.269 50.470 10.735

ATOM 1306 63C0 GRA 1 73.568 54.788 11.260

ATOM 1307 73C0 GRA 1 75.867 59.106 11.784

ATOM 1308 83C0 GRA 1 78.166 63.425 12.309

ATOM 1309 93C0 GRA 1 80.465 67.743 12.833

ATOM 1310 03C1 GRA 1 60.090 20.406 7.260

ATOM 1311 13C1 GRA 1 62.389 24.724 7.785

ATOM 1312 23C1 GRA 1 64.688 29.043 8.309

ATOM 1313 33C1 GRA 1 66.987 33.361 8.834

ATOM 1314 43C1 GRA 1 69.286 37.679 9.358

ATOM 1315 53C1 GRA 1 71.585 41.997 9.883

ATOM 1316 63C1 GRA 1 73.884 46.315 10.407

ATOM 1317 73C1 GRA 1 76.183 50.633 10.932

ATOM 1318 83C1 GRA 1 78.481 54.951 11.456

ATOM 1319 93C1 GRA 1 80.780 59.270 11.981

ATOM 1320 03C2 GRA 1 65.004 20.570 7.456

ATOM 1321 13C2 GRA 1 67.303 24.888 7.981

ATOM 1322 23C2 GRA 1 69.602 29.206 8.505

ATOM 1323 33C2 GRA 1 71.900 33.524 9.030

ATOM 1324 43C2 GRA 1 74.199 37.842 9.554

ATOM 1325 53C2 GRA 1 76.498 42.160 10.079

ATOM 1326 63C2 GRA 1 78.797 46.478 10.603

ATOM 1327 73C2 GRA 1 81.096 50.797 11.128

ATOM 1328 83C2 GRA 1 69.917 20.733 7.653

ATOM 1329 93C2 GRA 1 72.216 25.051 8.177

ATOM 1330 03C3 GRA 1 74.515 29.369 8.702

ATOM 1331 13C3 GRA 1 76.814 33.687 9.226

ATOM 1332 23C3 GRA 1 79.113 38.005 9.751

ATOM 1333 33C3 GRA 1 81.412 42.323 10.275

ATOM 1334 43C3 GRA 1 74.830 20.896 7.849

ATOM 1335 53C3 GRA 1 77.129 25.214 8.374

ATOM 1336 63C3 GRA 1 79.428 29.533 8.898

ATOM 1337 73C3 GRA 1 81.727 33.851 9.423

ATOM 1338 83C3 GRA 1 79.743 21.059 8.045

ATOM 1339 93C3 GRA 1 82.042 25.378 8.570

ATOM 1340 03C4 GRA 1 10.528 63.298 9.822

ATOM 1341 13C4 GRA 1 12.827 67.617 10.347

ATOM 1342 23C4 GRA 1 10.844 54.825 8.970

ATOM 1343 33C4 GRA 1 13.142 59.143 9.494

ATOM 1344 43C4 GRA 1 15.441 63.462 10.019

ATOM 1345 53C4 GRA 1 17.740 67.780 10.543

ATOM 1346 63C4 GRA 1 11.159 46.352 8.117

ATOM 1347 73C4 GRA 1 13.458 50.670 8.642

ATOM 1348 83C4 GRA 1 15.757 54.988 9.166

ATOM 1349 93C4 GRA 1 18.056 59.307 9.691

ATOM 1350 03C5 GRA 1 20.355 63.625 10.215

ATOM 1351 13C5 GRA 1 22.654 67.943 10.740

ATOM 1352 23C5 GRA 1 11.475 37.879 7.264

ATOM 1353 33C5 GRA 1 13.774 42.197 7.789

ATOM 1354 43C5 GRA 1 16.073 46.515 8.313

ATOM 1355 53C5 GRA 1 18.372 50.834 8.838

ATOM 1356 63C5 GRA 1 20.671 55.152 9.362

ATOM 1357 73C5 GRA 1 22.970 59.470 9.887

ATOM 1358 83C5 GRA 1 25.268 63.788 10.411

ATOM 1359 93C5 GRA 1 27.567 68.106 10.936

ATOM 1360 03C6 GRA 1 11.791 29.406 6.412

ATOM 1361 13C6 GRA 1 14.090 33.724 6.936

ATOM 1362 23C6 GRA 1 16.389 38.042 7.461

ATOM 1363 33C6 GRA 1 18.687 42.360 7.985

ATOM 1364 43C6 GRA 1 20.986 46.679 8.510

ATOM 1365 53C6 GRA 1 23.285 50.997 9.034

ATOM 1366 63C6 GRA 1 25.584 55.315 9.559

ATOM 1367 73C6 GRA 1 27.883 59.633 10.083

ATOM 1368 83C6 GRA 1 30.182 63.951 10.608

ATOM 1369 93C6 GRA 1 32.481 68.269 11.132

ATOM 1370 03C7 GRA 1 12.106 20.933 5.559

ATOM 1371 13C7 GRA 1 14.405 25.251 6.083

ATOM 1372 23C7 GRA 1 16.704 29.569 6.608

ATOM 1373 33C7 GRA 1 19.003 33.887 7.132

ATOM 1374 43C7 GRA 1 21.302 38.205 7.657

ATOM 1375 53C7 GRA 1 23.601 42.524 8.182

ATOM 1376 63C7 GRA 1 25.900 46.842 8.706

ATOM 1377 73C7 GRA 1 28.199 51.160 9.231

ATOM 1378 83C7 GRA 1 30.498 55.478 9.755

ATOM 1379 93C7 GRA 1 32.797 59.796 10.280

ATOM 1380 03C8 GRA 1 35.095 64.114 10.804

ATOM 1381 13C8 GRA 1 37.394 68.432 11.329

ATOM 1382 23C8 GRA 1 14.720 16.778 5.231

ATOM 1383 33C8 GRA 1 17.019 21.096 5.755

ATOM 1384 43C8 GRA 1 19.318 25.415 6.280

ATOM 1385 53C8 GRA 1 21.617 29.733 6.804

ATOM 1386 63C8 GRA 1 23.916 34.051 7.329

ATOM 1387 73C8 GRA 1 26.215 38.369 7.853

ATOM 1388 83C8 GRA 1 28.514 42.687 8.378

ATOM 1389 93C8 GRA 1 30.812 47.005 8.902

ATOM 1390 03C9 GRA 1 33.111 51.323 9.427

ATOM 1391 13C9 GRA 1 35.410 55.642 9.951

ATOM 1392 23C9 GRA 1 37.709 59.960 10.476

ATOM 1393 33C9 GRA 1 40.008 64.278 11.000

ATOM 1394 43C9 GRA 1 42.307 68.596 11.525

ATOM 1395 53C9 GRA 1 19.634 16.942 5.427

ATOM 1396 63C9 GRA 1 21.933 21.260 5.952

ATOM 1397 73C9 GRA 1 24.231 25.578 6.476

ATOM 1398 83C9 GRA 1 26.530 29.896 7.001

ATOM 1399 93C9 GRA 1 28.829 34.214 7.525

ATOM 1400 04C0 GRA 1 31.128 38.532 8.050

ATOM 1401 14C0 GRA 1 33.427 42.850 8.574

ATOM 1402 24C0 GRA 1 35.726 47.168 9.099

ATOM 1403 34C0 GRA 1 38.025 51.487 9.623

ATOM 1404 44C0 GRA 1 40.324 55.805 10.148

ATOM 1405 54C0 GRA 1 42.623 60.123 10.672

ATOM 1406 64C0 GRA 1 44.922 64.441 11.197

ATOM 1407 74C0 GRA 1 47.220 68.759 11.721

ATOM 1408 84C0 GRA 1 24.547 17.105 5.623

ATOM 1409 94C0 GRA 1 26.846 21.423 6.148

ATOM 1410 04C1 GRA 1 29.145 25.741 6.672

ATOM 1411 14C1 GRA 1 31.444 30.059 7.197

ATOM 1412 24C1 GRA 1 33.743 34.377 7.722

ATOM 1413 34C1 GRA 1 36.042 38.695 8.246

ATOM 1414 44C1 GRA 1 38.341 43.014 8.771

ATOM 1415 54C1 GRA 1 40.639 47.332 9.295

ATOM 1416 64C1 GRA 1 42.938 51.650 9.820

ATOM 1417 74C1 GRA 1 45.237 55.968 10.344

ATOM 1418 84C1 GRA 1 47.536 60.286 10.869

ATOM 1419 94C1 GRA 1 49.835 64.604 11.393

ATOM 1420 04C2 GRA 1 52.134 68.922 11.918

ATOM 1421 14C2 GRA 1 29.461 17.268 5.820

ATOM 1422 24C2 GRA 1 31.760 21.586 6.344

ATOM 1423 34C2 GRA 1 34.058 25.904 6.869

ATOM 1424 44C2 GRA 1 36.357 30.222 7.393

ATOM 1425 54C2 GRA 1 38.656 34.540 7.918

ATOM 1426 64C2 GRA 1 40.955 38.859 8.442

ATOM 1427 74C2 GRA 1 43.254 43.177 8.967

ATOM 1428 84C2 GRA 1 45.553 47.495 9.491

ATOM 1429 94C2 GRA 1 47.852 51.813 10.016

ATOM 1430 04C3 GRA 1 50.151 56.131 10.540

ATOM 1431 14C3 GRA 1 52.450 60.449 11.065

ATOM 1432 24C3 GRA 1 54.749 64.767 11.589

ATOM 1433 34C3 GRA 1 57.047 69.086 12.114

ATOM 1434 44C3 GRA 1 34.374 17.431 6.016

ATOM 1435 54C3 GRA 1 36.673 21.749 6.541

ATOM 1436 64C3 GRA 1 38.972 26.067 7.065

ATOM 1437 74C3 GRA 1 41.271 30.385 7.590

ATOM 1438 84C3 GRA 1 43.570 34.704 8.114

ATOM 1439 94C3 GRA 1 45.869 39.022 8.639

ATOM 1440 04C4 GRA 1 48.168 43.340 9.163

ATOM 1441 14C4 GRA 1 50.466 47.658 9.688

ATOM 1442 24C4 GRA 1 52.765 51.976 10.212

ATOM 1443 34C4 GRA 1 55.064 56.294 10.737

ATOM 1444 44C4 GRA 1 57.363 60.612 11.261

ATOM 1445 54C4 GRA 1 59.662 64.931 11.786

ATOM 1446 64C4 GRA 1 61.961 69.249 12.310

ATOM 1447 74C4 GRA 1 39.288 17.594 6.212

ATOM 1448 84C4 GRA 1 41.587 21.912 6.737

ATOM 1449 94C4 GRA 1 43.885 26.230 7.261

ATOM 1450 04C5 GRA 1 46.184 30.549 7.786

ATOM 1451 14C5 GRA 1 48.483 34.867 8.311

ATOM 1452 24C5 GRA 1 50.782 39.185 8.835

ATOM 1453 34C5 GRA 1 53.081 43.503 9.360

ATOM 1454 44C5 GRA 1 55.380 47.821 9.884

ATOM 1455 54C5 GRA 1 57.679 52.139 10.409

ATOM 1456 64C5 GRA 1 59.978 56.457 10.933

ATOM 1457 74C5 GRA 1 62.277 60.776 11.458

ATOM 1458 84C5 GRA 1 64.576 65.094 11.982

ATOM 1459 94C5 GRA 1 66.874 69.412 12.507

ATOM 1460 04C6 GRA 1 44.200 17.758 6.409

ATOM 1461 14C6 GRA 1 46.499 22.076 6.933

ATOM 1462 24C6 GRA 1 48.798 26.394 7.458

ATOM 1463 34C6 GRA 1 51.097 30.712 7.982

ATOM 1464 44C6 GRA 1 53.396 35.030 8.507

ATOM 1465 54C6 GRA 1 55.695 39.348 9.031

ATOM 1466 64C6 GRA 1 57.994 43.667 9.556

ATOM 1467 74C6 GRA 1 60.293 47.985 10.080

ATOM 1468 84C6 GRA 1 62.592 52.303 10.605

ATOM 1469 94C6 GRA 1 64.890 56.621 11.129

ATOM 1470 04C7 GRA 1 67.189 60.939 11.654

ATOM 1471 14C7 GRA 1 69.488 65.257 12.178

ATOM 1472 24C7 GRA 1 71.787 69.575 12.703

ATOM 1473 34C7 GRA 1 49.114 17.921 6.605

ATOM 1474 44C7 GRA 1 51.413 22.239 7.130

ATOM 1475 54C7 GRA 1 53.712 26.557 7.654

ATOM 1476 64C7 GRA 1 56.011 30.875 8.179

ATOM 1477 74C7 GRA 1 58.309 35.194 8.703

ATOM 1478 84C7 GRA 1 60.608 39.512 9.228

ATOM 1479 94C7 GRA 1 62.907 43.830 9.752

ATOM 1480 04C8 GRA 1 65.206 48.148 10.277

ATOM 1481 14C8 GRA 1 67.505 52.466 10.801

ATOM 1482 24C8 GRA 1 69.804 56.784 11.326

ATOM 1483 34C8 GRA 1 72.103 61.102 11.850

ATOM 1484 44C8 GRA 1 74.402 65.421 12.375

ATOM 1485 54C8 GRA 1 76.701 69.739 12.899

ATOM 1486 64C8 GRA 1 54.027 18.084 6.801

ATOM 1487 74C8 GRA 1 56.326 22.402 7.326

ATOM 1488 84C8 GRA 1 58.625 26.720 7.851

ATOM 1489 94C8 GRA 1 60.924 31.039 8.375

ATOM 1490 04C9 GRA 1 63.223 35.357 8.900

ATOM 1491 14C9 GRA 1 65.522 39.675 9.424

ATOM 1492 24C9 GRA 1 67.821 43.993 9.949

ATOM 1493 34C9 GRA 1 70.120 48.311 10.473

ATOM 1494 44C9 GRA 1 72.419 52.629 10.998

ATOM 1495 54C9 GRA 1 74.717 56.947 11.522

ATOM 1496 64C9 GRA 1 77.016 61.266 12.047

ATOM 1497 74C9 GRA 1 79.315 65.584 12.571

ATOM 1498 84C9 GRA 1 81.614 69.902 13.096

ATOM 1499 94C9 GRA 1 58.941 18.247 6.998

ATOM 1500 05C0 GRA 1 61.240 22.565 7.522

ATOM 1501 15C0 GRA 1 63.539 26.884 8.047

ATOM 1502 25C0 GRA 1 65.838 31.202 8.571

ATOM 1503 35C0 GRA 1 68.136 35.520 9.096

ATOM 1504 45C0 GRA 1 70.435 39.838 9.620

ATOM 1505 55C0 GRA 1 72.734 44.156 10.145

ATOM 1506 65C0 GRA 1 75.033 48.474 10.669

ATOM 1507 75C0 GRA 1 77.332 52.792 11.194

ATOM 1508 85C0 GRA 1 79.631 57.111 11.718

ATOM 1509 95C0 GRA 1 81.930 61.429 12.243

ATOM 1510 05C1 GRA 1 63.854 18.410 7.194

ATOM 1511 15C1 GRA 1 66.153 22.729 7.719

ATOM 1512 25C1 GRA 1 68.452 27.047 8.243

ATOM 1513 35C1 GRA 1 70.751 31.365 8.768

ATOM 1514 45C1 GRA 1 73.050 35.683 9.292

ATOM 1515 55C1 GRA 1 75.349 40.001 9.817

ATOM 1516 65C1 GRA 1 77.648 44.319 10.341

ATOM 1517 75C1 GRA 1 79.947 48.637 10.866

ATOM 1518 85C1 GRA 1 82.246 52.956 11.390

ATOM 1519 95C1 GRA 1 68.768 18.574 7.390

ATOM 1520 05C2 GRA 1 71.067 22.892 7.915

ATOM 1521 15C2 GRA 1 73.366 27.210 8.440

ATOM 1522 25C2 GRA 1 75.665 31.528 8.964

ATOM 1523 35C2 GRA 1 77.963 35.846 9.489

ATOM 1524 45C2 GRA 1 80.262 40.164 10.013

ATOM 1525 55C2 GRA 1 82.561 44.482 10.538

ATOM 1526 65C2 GRA 1 73.680 18.737 7.587

ATOM 1527 75C2 GRA 1 75.979 23.055 8.111

ATOM 1528 85C2 GRA 1 78.278 27.374 8.636

ATOM 1529 95C2 GRA 1 80.577 31.692 9.160

ATOM 1530 05C3 GRA 1 82.876 36.010 9.685

ATOM 1531 15C3 GRA 1 78.594 18.900 7.783

ATOM 1532 25C3 GRA 1 80.893 23.219 8.308

ATOM 1533 35C3 GRA 1 83.192 27.537 8.832

ATOM 1534 45C3 GRA 1 83.507 19.064 7.980

TER

END
