# Supplementary material for: Exploring the Influence of Carbon Nanoparticles on the Formation of β-Sheet-Rich Oligomers of IAPP22–28 Peptide by Molecular Dynamics Simulation
Source: PLoS One. 2013 Jun 5;8(6):e65579. doi: 10.1371/journal.pone.0065579 (PMC3674003; doi:10.1371/journal.pone.0065579)
Supplement: Text S4 — Coordinates of SWCNT. (DOC) [file pone.0065579.s006.doc]

**Text S4. Coordinates of SWCNT.**

ATOM 449 C1 CNT 1 39.360 21.068 34.908

ATOM 450 C2 CNT 1 40.075 22.170 30.203

ATOM 451 C3 CNT 1 39.761 22.571 31.517

ATOM 452 C4 CNT 1 39.717 21.619 32.556

ATOM 453 C5 CNT 1 38.727 23.232 34.051

ATOM 454 C6 CNT 1 39.404 22.020 33.870

ATOM 455 C7 CNT 1 38.037 23.439 35.262

ATOM 456 C8 CNT 1 40.790 23.271 25.499

ATOM 457 C9 CNT 1 40.476 23.673 26.813

ATOM 458 C10 CNT 1 40.432 22.721 27.851

ATOM 459 C11 CNT 1 39.442 24.334 29.346

ATOM 460 C12 CNT 1 40.119 23.122 29.165

ATOM 461 C13 CNT 1 38.752 24.541 30.557

ATOM 462 C14 CNT 1 39.084 23.783 31.698

ATOM 463 C15 CNT 1 37.137 24.603 32.862

ATOM 464 C16 CNT 1 38.394 23.990 32.910

ATOM 465 C17 CNT 1 36.267 24.432 33.958

ATOM 466 C18 CNT 1 36.780 24.052 35.215

ATOM 467 C19 CNT 1 41.505 24.373 20.794

ATOM 468 C20 CNT 1 41.191 24.774 22.108

ATOM 469 C21 CNT 1 41.147 23.822 23.147

ATOM 470 C22 CNT 1 40.156 25.436 24.642

ATOM 471 C23 CNT 1 40.833 24.223 24.461

ATOM 472 C24 CNT 1 39.466 25.643 25.853

ATOM 473 C25 CNT 1 39.799 24.885 26.994

ATOM 474 C26 CNT 1 37.852 25.704 28.157

ATOM 475 C27 CNT 1 39.109 25.092 28.205

ATOM 476 C28 CNT 1 36.982 25.534 29.253

ATOM 477 C29 CNT 1 37.495 25.154 30.509

ATOM 478 C30 CNT 1 35.267 24.763 31.347

ATOM 479 C31 CNT 1 36.625 24.983 31.605

ATOM 480 C32 CNT 1 34.480 24.173 32.358

ATOM 481 C33 CNT 1 34.909 24.212 33.700

ATOM 482 C34 CNT 1 33.183 22.654 34.341

ATOM 483 C35 CNT 1 34.123 23.622 34.710

ATOM 484 C36 CNT 1 42.220 25.475 16.089

ATOM 485 C37 CNT 1 41.906 25.876 17.403

ATOM 486 C38 CNT 1 41.862 24.924 18.441

ATOM 487 C39 CNT 1 40.871 26.538 19.936

ATOM 488 C40 CNT 1 41.548 25.325 19.755

ATOM 489 C41 CNT 1 40.181 26.744 21.149

ATOM 490 C42 CNT 1 40.514 25.987 22.289

ATOM 491 C43 CNT 1 38.567 26.806 23.453

ATOM 492 C44 CNT 1 39.824 26.194 23.501

ATOM 493 C45 CNT 1 37.697 26.635 24.549

ATOM 494 C46 CNT 1 38.209 26.255 25.805

ATOM 495 C47 CNT 1 35.982 25.864 26.643

ATOM 496 C48 CNT 1 37.340 26.085 26.901

ATOM 497 C49 CNT 1 35.195 25.275 27.653

ATOM 498 C50 CNT 1 35.624 25.313 28.995

ATOM 499 C51 CNT 1 33.898 23.756 29.636

ATOM 500 C52 CNT 1 34.838 24.724 30.005

ATOM 501 C53 CNT 1 33.428 22.867 30.624

ATOM 502 C54 CNT 1 33.540 23.205 31.989

ATOM 503 C55 CNT 1 32.907 20.969 32.636

ATOM 504 C56 CNT 1 33.070 22.316 32.977

ATOM 505 C57 CNT 1 32.863 20.017 33.674

ATOM 506 C58 CNT 1 41.586 27.639 15.232

ATOM 507 C59 CNT 1 42.263 26.427 15.051

ATOM 508 C60 CNT 1 40.896 27.846 16.444

ATOM 509 C61 CNT 1 41.229 27.088 17.584

ATOM 510 C62 CNT 1 39.282 27.908 18.748

ATOM 511 C63 CNT 1 40.539 27.295 18.796

ATOM 512 C64 CNT 1 38.412 27.737 19.844

ATOM 513 C65 CNT 1 38.924 27.357 21.101

ATOM 514 C66 CNT 1 36.697 26.966 21.939

ATOM 515 C67 CNT 1 38.055 27.186 22.197

ATOM 516 C68 CNT 1 35.910 26.376 22.949

ATOM 517 C69 CNT 1 36.339 26.415 24.291

ATOM 518 C70 CNT 1 34.613 24.857 24.931

ATOM 519 C71 CNT 1 35.553 25.826 25.301

ATOM 520 C72 CNT 1 34.142 23.969 25.920

ATOM 521 C73 CNT 1 34.255 24.307 27.283

ATOM 522 C74 CNT 1 33.622 22.071 27.931

ATOM 523 C75 CNT 1 33.785 23.418 28.272

ATOM 524 C76 CNT 1 33.578 21.118 28.969

ATOM 525 C77 CNT 1 33.265 21.520 30.283

ATOM 526 C78 CNT 1 33.898 19.355 31.141

ATOM 527 C79 CNT 1 33.221 20.567 31.322

ATOM 528 C80 CNT 1 34.230 18.597 32.282

ATOM 529 C81 CNT 1 33.540 18.804 33.493

ATOM 530 C82 CNT 1 39.127 28.839 15.140

ATOM 531 C83 CNT 1 39.639 28.459 16.396

ATOM 532 C84 CNT 1 37.412 28.068 17.234

ATOM 533 C85 CNT 1 38.770 28.288 17.492

ATOM 534 C86 CNT 1 36.625 27.478 18.244

ATOM 535 C87 CNT 1 37.054 27.517 19.586

ATOM 536 C88 CNT 1 35.328 25.959 20.227

ATOM 537 C89 CNT 1 36.268 26.927 20.597

ATOM 538 C90 CNT 1 34.857 25.071 21.215

ATOM 539 C91 CNT 1 34.970 25.408 22.579

ATOM 540 C92 CNT 1 34.337 23.172 23.227

ATOM 541 C93 CNT 1 34.500 24.520 23.568

ATOM 542 C94 CNT 1 34.293 22.220 24.265

ATOM 543 C95 CNT 1 33.979 22.621 25.579

ATOM 544 C96 CNT 1 34.613 20.457 26.436

ATOM 545 C97 CNT 1 33.936 21.669 26.617

ATOM 546 C98 CNT 1 34.945 19.699 27.577

ATOM 547 C99 CNT 1 34.255 19.906 28.788

ATOM 548 0C10 CNT 1 35.845 18.536 29.977

ATOM 549 1C10 CNT 1 34.588 19.148 29.929

ATOM 550 2C10 CNT 1 36.357 18.155 31.234

ATOM 551 3C10 CNT 1 35.487 17.985 32.330

ATOM 552 4C10 CNT 1 37.358 17.825 33.844

ATOM 553 5C10 CNT 1 36.000 17.605 33.586

ATOM 554 6C10 CNT 1 37.769 28.618 14.882

ATOM 555 7C10 CNT 1 36.043 27.061 15.522

ATOM 556 8C10 CNT 1 36.983 28.029 15.892

ATOM 557 9C10 CNT 1 35.572 26.172 16.510

ATOM 558 0C11 CNT 1 35.685 26.510 17.874

ATOM 559 1C11 CNT 1 35.052 24.274 18.522

ATOM 560 2C11 CNT 1 35.215 25.622 18.862

ATOM 561 3C11 CNT 1 35.008 23.322 19.561

ATOM 562 4C11 CNT 1 34.694 23.723 20.875

ATOM 563 5C11 CNT 1 35.328 21.558 21.732

ATOM 564 6C11 CNT 1 34.651 22.771 21.913

ATOM 565 7C11 CNT 1 35.660 20.801 22.873

ATOM 566 8C11 CNT 1 34.970 21.008 24.084

ATOM 567 9C11 CNT 1 36.560 19.637 25.273

ATOM 568 0C12 CNT 1 35.303 20.250 25.225

ATOM 569 1C12 CNT 1 37.072 19.257 26.529

ATOM 570 2C12 CNT 1 36.202 19.087 27.625

ATOM 571 3C12 CNT 1 38.073 18.927 29.139

ATOM 572 4C12 CNT 1 36.715 18.706 28.881

ATOM 573 5C12 CNT 1 38.502 18.965 30.481

ATOM 574 6C12 CNT 1 37.715 18.376 31.492

ATOM 575 7C12 CNT 1 39.084 19.383 33.204

ATOM 576 8C12 CNT 1 38.144 18.414 32.834

ATOM 577 9C12 CNT 1 39.197 19.720 34.568

ATOM 578 0C13 CNT 1 35.767 25.376 13.818

ATOM 579 1C13 CNT 1 35.930 26.723 14.158

ATOM 580 2C13 CNT 1 35.723 24.423 14.856

ATOM 581 3C13 CNT 1 35.409 24.825 16.170

ATOM 582 4C13 CNT 1 36.043 22.660 17.027

ATOM 583 5C13 CNT 1 35.366 23.873 17.208

ATOM 584 6C13 CNT 1 36.375 21.902 18.168

ATOM 585 7C13 CNT 1 35.685 22.109 19.380

ATOM 586 8C13 CNT 1 37.275 20.739 20.568

ATOM 587 9C13 CNT 1 36.018 21.351 20.521

ATOM 588 0C14 CNT 1 37.787 20.359 21.825

ATOM 589 1C14 CNT 1 36.917 20.188 22.920

ATOM 590 2C14 CNT 1 38.788 20.028 24.435

ATOM 591 3C14 CNT 1 37.430 19.808 24.177

ATOM 592 4C14 CNT 1 39.216 20.067 25.777

ATOM 593 5C14 CNT 1 38.430 19.478 26.787

ATOM 594 6C14 CNT 1 39.799 20.484 28.499

ATOM 595 7C14 CNT 1 38.859 19.516 28.129

ATOM 596 8C14 CNT 1 39.912 20.822 29.862

ATOM 597 9C14 CNT 1 39.442 19.934 30.851

ATOM 598 0C15 CNT 1 39.554 20.271 32.215

ATOM 599 1C15 CNT 1 37.090 23.004 13.464

ATOM 600 2C15 CNT 1 36.400 23.211 14.675

ATOM 601 3C15 CNT 1 37.990 21.841 15.863

ATOM 602 4C15 CNT 1 36.733 22.453 15.816

ATOM 603 5C15 CNT 1 38.502 21.461 17.120

ATOM 604 6C15 CNT 1 37.632 21.290 18.215

ATOM 605 7C15 CNT 1 39.502 21.130 19.731

ATOM 606 8C15 CNT 1 38.144 20.910 19.473

ATOM 607 9C15 CNT 1 39.931 21.169 21.073

ATOM 608 0C16 CNT 1 39.145 20.579 22.083

ATOM 609 1C16 CNT 1 40.514 21.586 23.794

ATOM 610 2C16 CNT 1 39.574 20.618 23.425

ATOM 611 3C16 CNT 1 40.627 21.924 25.158

ATOM 612 4C16 CNT 1 40.156 21.035 26.146

ATOM 613 5C16 CNT 1 40.269 21.373 27.510

ATOM 614 6C16 CNT 1 38.347 22.392 13.511

ATOM 615 7C16 CNT 1 40.217 22.232 15.026

ATOM 616 8C16 CNT 1 38.859 22.011 14.768

ATOM 617 9C16 CNT 1 40.646 22.270 16.368

ATOM 618 0C17 CNT 1 39.860 21.681 17.378

ATOM 619 1C17 CNT 1 41.229 22.688 19.089

ATOM 620 2C17 CNT 1 40.289 21.720 18.720

ATOM 621 3C17 CNT 1 41.342 23.025 20.454

ATOM 622 4C17 CNT 1 40.871 22.137 21.442

ATOM 623 5C17 CNT 1 40.984 22.474 22.806

ATOM 624 6C17 CNT 1 41.944 23.789 14.385

ATOM 625 7C17 CNT 1 41.004 22.821 14.016

ATOM 626 8C17 CNT 1 42.057 24.127 15.749

ATOM 627 9C17 CNT 1 41.586 23.239 16.737

ATOM 628 0C18 CNT 1 41.699 23.576 18.101

ATOM 629 1C18 CNT 1 36.797 23.735 12.322

ATOM 630 2C18 CNT 1 36.095 24.943 12.499

ATOM 631 3C18 CNT 1 39.278 22.575 12.428

ATOM 632 4C18 CNT 1 40.640 22.821 12.692

ATOM 633 5C18 CNT 1 42.445 24.750 13.419

ATOM 634 6C18 CNT 1 42.585 26.109 13.758

ATOM 635 7C18 CNT 1 41.220 28.461 14.101

ATOM 636 8C18 CNT 1 39.945 29.056 14.047

ATOM 637 9C18 CNT 1 37.295 28.580 13.533

ATOM 638 0C19 CNT 1 36.368 27.588 13.160

ATOM 639 1C19 CNT 1 37.480 23.518 11.199

ATOM 640 2C19 CNT 1 38.755 22.923 11.254

ATOM 641 3C19 CNT 1 41.404 23.399 11.768

ATOM 642 4C19 CNT 1 42.332 24.390 12.141

ATOM 643 5C19 CNT 1 42.604 27.037 12.802

ATOM 644 6C19 CNT 1 41.903 28.245 12.978

ATOM 645 7C19 CNT 1 38.060 29.159 12.608

ATOM 646 8C19 CNT 1 39.422 29.404 12.873

ATOM 647 9C19 CNT 1 36.115 25.869 11.542

ATOM 648 0C20 CNT 1 36.255 27.230 11.882

ATOM 649 1C20 CNT 1 39.563 23.535 10.275

ATOM 650 2C20 CNT 1 40.851 23.766 10.525

ATOM 651 3C20 CNT 1 36.836 25.642 10.354

ATOM 652 4C20 CNT 1 37.500 24.498 10.187

ATOM 653 5C20 CNT 1 37.063 27.842 10.904

ATOM 654 6C20 CNT 1 37.940 28.779 11.256

ATOM 655 7C20 CNT 1 40.144 29.175 11.684

ATOM 656 8C20 CNT 1 41.350 28.612 11.735

ATOM 657 9C20 CNT 1 42.353 25.371 11.129

ATOM 658 0C21 CNT 1 42.485 26.657 11.450

ATOM 659 1C21 CNT 1 38.788 24.509 9.617

ATOM 660 2C21 CNT 1 37.423 26.859 9.960

ATOM 661 3C21 CNT 1 39.228 28.790 10.686

ATOM 662 4C21 CNT 1 41.709 27.631 10.792

ATOM 663 5C21 CNT 1 41.437 24.985 10.131

ATOM 664 6C21 CNT 1 39.568 27.861 9.793

ATOM 665 7C21 CNT 1 40.843 27.266 9.847

ATOM 666 8C21 CNT 1 40.703 25.906 9.507

ATOM 667 9C21 CNT 1 39.341 25.661 9.243

ATOM 668 0C22 CNT 1 38.640 26.869 9.419

ATOM 669 1C22 CNT 1 35.136 17.470 34.592

ATOM 670 2C22 CNT 1 33.870 18.086 34.545

ATOM 671 3C22 CNT 1 37.794 17.899 35.097

ATOM 672 4C22 CNT 1 38.741 18.874 35.469

ATOM 673 5C22 CNT 1 39.059 21.513 36.135

ATOM 674 6C22 CNT 1 38.379 22.733 36.318

ATOM 675 7C22 CNT 1 35.917 23.934 36.225

ATOM 676 8C22 CNT 1 34.550 23.713 35.965

ATOM 677 9C22 CNT 1 32.710 21.815 35.241

ATOM 678 0C23 CNT 1 32.547 20.460 34.899

ATOM 679 1C23 CNT 1 35.650 17.100 35.807

ATOM 680 2C23 CNT 1 37.017 17.321 36.066

ATOM 681 3C23 CNT 1 38.858 19.218 36.791

ATOM 682 4C23 CNT 1 39.022 20.575 37.133

ATOM 683 5C23 CNT 1 37.698 22.947 37.487

ATOM 684 6C23 CNT 1 36.432 23.565 37.440

ATOM 685 7C23 CNT 1 32.828 22.160 36.563

ATOM 686 8C23 CNT 1 33.774 23.134 36.935

ATOM 687 9C23 CNT 1 33.189 18.300 35.714

ATOM 688 0C24 CNT 1 32.509 19.520 35.897

ATOM 689 1C24 CNT 1 37.168 17.726 37.285

ATOM 690 2C24 CNT 1 38.063 18.648 37.637

ATOM 691 3C24 CNT 1 33.762 17.952 36.820

ATOM 692 4C24 CNT 1 34.958 17.368 36.865

ATOM 693 5C24 CNT 1 32.661 19.925 37.115

ATOM 694 6C24 CNT 1 32.816 21.208 37.439

ATOM 695 7C24 CNT 1 34.346 22.786 38.041

ATOM 696 8C24 CNT 1 35.638 22.995 38.286

ATOM 697 9C24 CNT 1 38.329 20.843 38.191

ATOM 698 0C25 CNT 1 37.685 21.996 38.363

ATOM 699 1C25 CNT 1 35.883 17.736 37.860

ATOM 700 2C25 CNT 1 33.422 18.937 37.767

ATOM 701 3C25 CNT 1 33.741 21.577 38.434

ATOM 702 4C25 CNT 1 36.400 22.006 38.939

ATOM 703 5C25 CNT 1 37.724 19.633 38.584

ATOM 704 6C25 CNT 1 34.462 20.641 39.051

ATOM 705 7C25 CNT 1 35.828 20.863 39.311

ATOM 706 8C25 CNT 1 36.509 19.643 39.129

ATOM 707 9C25 CNT 1 35.563 18.669 38.757

ATOM 708 0C26 CNT 1 34.297 19.285 38.709

TER

END
